# Synthesis and biological evaluation of novel quinoline-piperidine scaffolds as antiplasmodium agents

**DOI:** 10.1016/j.ejmech.2020.112330

**Published:** 2020-07-15

**Authors:** Tim Van de Walle, Maya Boone, Julie Van Puyvelde, Jill Combrinck, Peter J. Smith, Kelly Chibale, Sven Mangelinckx, Matthias D’hooghe

**Affiliations:** a SynBioC Research Group, Department of Green Chemistry and Technology, Faculty of Bioscience Engineering, Ghent University, Coupure Links 653, B-9000, Ghent, Belgium; b Division of Clinical Pharmacology, Department of Medicine, Medical School, University of Cape Town, K45, OMB, Groote Schuur Hospital, Observatory, 7925, South Africa; c Wellcome Centre for Infectious Diseases Research in Africa, Institute of Infectious Disease and Molecular Medicine, South Africa; d South African Medical Research Council Drug Discovery and Development Research Unit, Department of Chemistry and Institute of Infectious Disease & Molecular Medicine, University of Cape Town, Rondebosch, 7701, South Africa

**Keywords:** Quinolines, Piperidines, Malaria, Chloroquine, Plasmodium

## Abstract

The parasitic disease malaria places almost half of the world’s population at risk of infection and is responsible for more than 400,000 deaths each year. The first-line treatment, artemisinin combination therapies (ACT) regimen, is under threat due to emerging resistance of *Plasmodium falciparum* strains in e.g. the Mekong delta. Therefore, the development of new antimalarial agents is crucial in order to circumvent the growing resistance. Chloroquine, the long-established antimalarial drug, still serves as model compound for the design of new quinoline analogues, resulting in numerous new active derivatives against chloroquine-resistant *P. falciparum* strains over the past twenty years. In this work, a set of functionalized quinoline analogues, decorated with a modified piperidine-containing side chain, was synthesized. Both amino- and (aminomethyl)quinolines were prepared, resulting in a total of 18 novel quinoline-piperidine conjugates representing four different chemical series. Evaluation of their *in vitro* antiplasmodium activity against a CQ-sensitive (NF54) and a CQ-resistant (K1) strain of *P. falciparum* unveiled highly potent activities in the nanomolar range against both strains for five 4-aminoquinoline derivatives. Moreover, no cytotoxicity was observed for all active compounds at the maximum concentration tested. These five new aminoquinoline hit structures are therefore of considerable value for antimalarial research and have the potency to be transformed into novel antimalarial agents upon further hit-to-lead optimization studies.

## Highlights

• Four different series of new quinoline analogues decorated with a piperidine-containing side chain were synthesized.
• Antiplasmodium assessments in NF54 (CQ-sensitive) and K1 (CQ-resistant) strains of *P. falciparum* were performed.
• Five 4-aminoquinoline-piperidine compounds displayed nanomolar activities against both NF54 and K1 and no CHO toxicity.

## Graphical abstract

**Figure:**
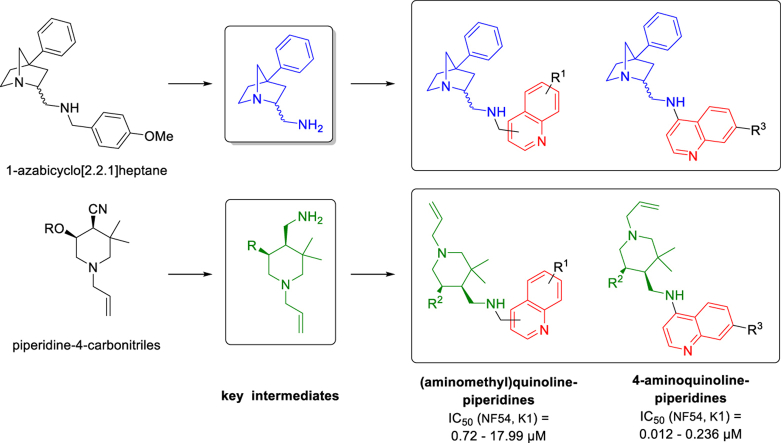

## 1 Introduction

Malaria refers to a fatal infection of red blood cells, caused by a protozoan parasite of the genus *Plasmodium* and transmitted to humans by *Anopheles* mosquitoes. Out of the five *Plasmodium* species that can cause malaria, *Plasmodium falciparum* and *Plasmodium vivax* are the most prevalent, with *P. falciparum* being the most lethal [1]. Despite numerous endeavours to reduce malaria incidence over the last decades, this tropical disease remains far from being vanquished. According to the latest WHO report, malaria gave rise to 228 million new cases and 405,000 deaths worldwide in 2018, mostly in the African region (93% of all new cases and 94% of all deaths) and in the age group of children under five years (67% of all deaths) [2]. These numbers confirm that malaria remains the most lethal parasitic infection worldwide. Current malaria control relies on three pillars: mosquito vector control with insecticides, treatment of infected people using antimalarial agents and, most recently, vaccination [3]. Although the employment of insecticides and vaccination are important prevention tools, the cure of infected people completely depends on antimalarial medicines. In the past, all antimalarial agents that have been deployed for the treatment of malaria, e.g. quinine, chloroquine, amodiaquine, sulfadoxine-pyrimethamine, mefloquine, piperaquine and halofantrine, shared the same fate [1,[4], [5], [6]]. *Plasmodium* parasites developed clinical resistance to these antiplasmodials in one area and then spread to other parts of the world [6,7]. Currently, artemisinin derivatives (artesunate, artemether and dihydroartemisinin (DHA)) are the first-line drugs to cure malaria, on the one hand as monotherapy for severe malaria cases, but mostly as part of the artemisinin combination therapies (ACT) regimen for uncomplicated malaria, whereby artemisinin derivatives are co-administered with a longer half-life antimalarial drug such as amodiaquine, piperaquine, mefloquine or sulfadoxine-pyrimethamine [1,6,8]. However, a reduced *in vivo* susceptibility of *P. falciparum* to artemisinins was already noticed more than ten years ago in the Cambodia-Thailand border area, which were the first signs of resistance development [9]. At present, an emerging multidrug resistance and growing ability to withstand ACT is the sobering situation [3,6]. A spread of this ACT-resistant *P. falciparum* from the Cambodia-Thailand area to other parts of the world, which has occurred in the past for both chloroquine and sulfadoxine-pyrimethamine, would result in a public health disaster, certainly when there is no plan B in terms of the availability of new potent antimalarial agents [1,3,6,10].

**Fig. 1:**
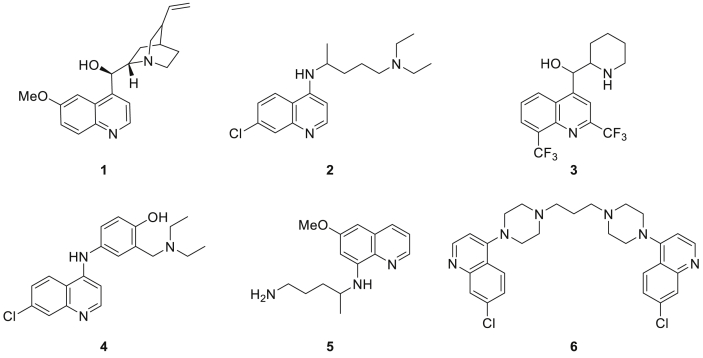
Examples of clinically used antimalarials containing a quinoline scaffold: quinine **1**, chloroquine **2**, mefloquine **3**, amodiaquine **4**, primaquine **5** and piperaquine **6**.

Throughout the history of antimalarial chemotherapy, the quinoline scaffold has always played a leading role, as can be seen from the numerous quinoline-containing antimalarial drugs such as quinine **1**, chloroquine **2**, mefloquine **3**, amodiaquine **4**, primaquine **5** and piperaquine **6** (Fig. 1). Although the *Plasmodium* parasite (especially *P. falciparum*) has developed resistance against all these quinolines, they are still of value as antimalarial medicines against more sensitive *Plasmodium* parasites or in combination with artemisinins [1,8,11]. Quinolines, in particular 4-aminoquinolines, based on the reference antimalarial chloroquine **2**, are still of high interest in the quest for novel antiplasmodium compounds, resulting in various highly potent quinolines over the last twenty years [3,4,[12], [13], [14]]. It is generally believed that quinine **1**, chloroquine **2**, mefloquine **3**, amodiaquine **4** (and by extension all 4-substituted quinolines) rely on the same mode of action against *Plasmodium* [15]. Human hemoglobin is digested in the digestive vacuole of the parasite to produce amino acids, which are needed for its growth. Heme, a toxic side product formed during this degradation, is normally bio-crystallized by the *Plasmodium* parasite into hemozoin, a non-toxic crystal form [16]. When administered to infected people, chloroquine **2** and other quinolines enter the acidic digestive vacuole (pH 5) of the *Plasmodium* parasite through diffusion, where they are protonated. These protonated forms are unable to diffuse back across the membrane and are thereby accumulated in high concentrations in this digestive vacuole [[17], [18], [19]]. They subsequently inhibit the crystallization of heme into hemozoin by forming a heme-quinoline complex, resulting in a build-up of toxic heme molecules and consequently death of the protozoans [4,13,20]. The *Plasmodium* parasite can gain resistance against established antimalarial drugs via two different mechanisms: by means of mutations in the drug target or via mutations in drug transporters [21,22]. For chloroquine **2** and mefloquine **3**, and presumably also other quinolines having the same mode of action, the latter mechanism is responsible for the developed resistance [6]. Although not fully understood, there is much evidence that multiple point mutations in the PfCRT (*P. falciparum* chloroquine resistance transporter) gene in case of chloroquine resistance and an increased copy-number of the pfmdr1 (*P. falciparum* multidrug resistance) gene in case of mefloquine resistance are involved [4,6,[23], [24], [25]]. Either way, these mutated transporters, located in the membrane of the parasite’s digestive vacuole, impair the accumulation of chloroquine **2** or mefloquine **3** inside the digestive vacuole, probably via an increased efflux mechanism. In that way, they prevent the inhibitory activity of these antimalarial drugs, simply because they cannot reach their target [6,17,21,[25], [26], [27]]. However, the target activity itself, i.e. binding to heme units, is not altered in these resistant *Plasmodium* strains and therefore remains very interesting to tackle, as this is a biological process that is unique to the malarial parasite, resulting in limited host toxicity [28]. Examining the potent chloroquine derivatives that have been designed over the past twenty years, a variety of side chain modifications, including the introduction of different heterocyclic moieties, has been applied and well tolerated without being detrimental to biological activity, while the pharmacophoric quinoline moiety is always maintained. Authors frequently refer to the term *hybrid structures*, i.e. the implementation of a second pharmacophore group as side chain onto the quinoline nucleus, with the rationale of obtaining a compound with a dual mode of action [3,4,12,14]. It is believed that these side chain modifications can also hinder binding and/or transport by mutated PfCRT in chloroquine-resistant (CQ-resistant) strains, due to steric hindrance [17,29]. Even though many of these so called *hybrid structures* possess very good antiplasmodium properties against CQ-resistant *P. falciparum*, there is not always experimental proof that this improved activity can truly be attributed to a dual mode of action, or solely to an excellent chloroquine-type mode of action that has surpassed resistance by circumventing the mutated transporter mechanisms. Regardless of which mechanism is responsible for their superior activity, the abundance of potent examples proves that the development of new chloroquine analogues with a functionalized side chain still constitutes a promising strategy in the quest for new antimalarial agents.

Within that framework, and inspired by the structural properties of quinine **1** and mefloquine **3**, we aimed to synthesize functionalized quinoline derivatives bearing a piperidine moiety in their side chain. In our opinion, the introduction of piperidine as heterocyclic structure in antimalarial quinoline analogues is underinvestigated compared to other heterocycles such as triazine, triazole and pyrimidine [3,4,12]. Furthermore, the introduction of a piperidine moiety will deliver a weakly basic side chain, which is presumed to be essential for uptake and accumulation of the antimalarial drug via pH trapping in the acidic digestive vacuole [17,30,31]. In analogy with quinine **1** and mefloquine **3**, we selected one bridged and one monocyclic functionalized piperidine scaffold, suitable for linking with different quinoline cores. This paper presents the synthesis of four different series of novel quinoline derivatives modified with a piperidine side chain and the assessment of their antiplasmodium activity against a CQ-sensitive strain (NF54) of *P. falciparum*. The most promising analogues were further evaluated for their potency in a CQ-resistant strain (K1) of *P. falciparum*, and for their cytotoxicity in a mammalian Chinese hamster ovary (CHO) cell line.

## 2 Results and discussion

### 2.1 Synthesis

In order to have convenient access to more decorated piperidine side chains, we decided to construct our contemplated quinoline-piperidine analogues from appropriate piperidine scaffolds, instead of commencing with the modification of a quinoline nucleus, as is most commonly done in the literature. To that end, two different piperidine compounds were selected, both bearing a functional group (an *N*-4-methoxybenzyl and a nitrile moiety, respectively) that can be converted into a free amino group, since this was the preferred route towards reaction with various quinolines. As a first heterocyclic scaffold, a bridged piperidine, i.e. 2-[*N*-(4-methoxybenzyl)aminomethyl]-4-phenyl-1-azabicyclo[2.2.1]heptane **7** was chosen on the basis of its similarity to the side chain present in quinine **1** and based on our in-house knowledge pertaining to the synthesis of this class of compounds. A series of 1-azabicyclo[2.2.1]heptanes had previously been synthesized in our group and evaluated for *in vitro* antiplasmodium activity [32]. Based on *in silico* docking studies of the most active compound, the moderate antiplasmodium activity observed was hypothesized to (partially) be attributed to inhibition of plasmepsin II, an enzyme essential for hemoglobin digestion in the parasite [32]. Therefore, it seemed reasonable to explore whether or not the observed moderate antiplasmodium activity could contribute to a potential dual mode of action in our target analogues.

**Scheme 1:**
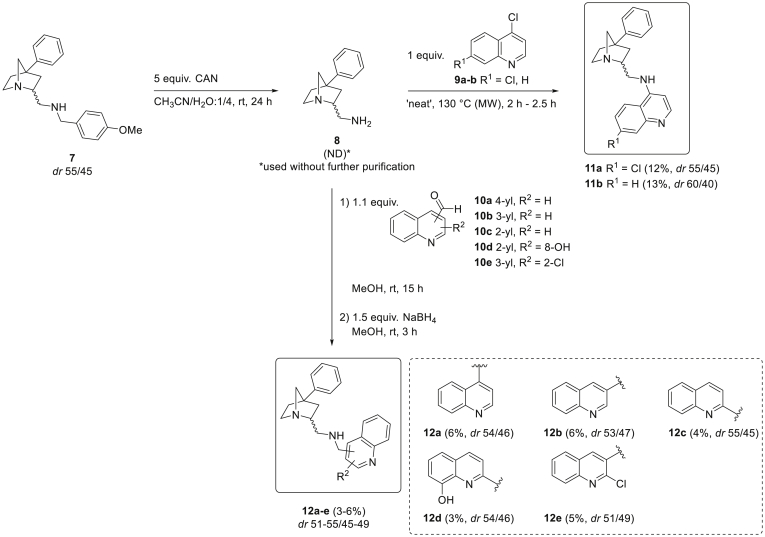
Synthetic route towards 4-aminoquinolines **11a-b** and (aminomethyl)quinolines **12a-e** functionalized with a 1-azabicyclo[2.2.1]heptane scaffold.

For the synthesis of the contemplated bridged piperidine-quinoline analogues, 1-azabicyclo[2.2.1]heptane **7**, bearing a *p*-methoxybenzyl (PMB) protecting group, was used as a starting point because of the inability to transform the other known 1-azabicyclo[2.2.1]heptanes (with *N*-benzyl, *N*-(4-chlorobenzyl) and *N*-(4-methylbenzyl)) [32] into the corresponding primary amine via common debenzylation methods. As described in the literature, bridged piperidine **7** was prepared by treatment of the corresponding 2-(bromomethyl)aziridine [[33], [34], [35]] with α-lithiated phenylacetonitrile in THF, followed by deprotonation with LDA and subsequent quenching with 1-bromo-2-chloroethane [36], and finally a one-step LiAlH_4_-mediated ring closure to afford the constrained piperidine (Synthesis and Experimental Data, see Supporting Information) [32]. It should be noted that the separation of both diastereomers of 1-azabicyclo[2.2.1]heptane **7** is possible via selective crystallization in ethanol [36], but for practical reasons in view of the reaction scale, constrained piperidine **7** was used as mixture of *endo*- and *exo*-isomers (*dr* 55/45) for further synthetic elaboration. Treatment of 1-azabicyclo[2.2.1]heptane **7** with cerium ammonium nitrate (CAN) in an acetonitrile/water (1/4) mixture resulted in primary amine **8** (Scheme 1). Purification using both normal phase or reversed phase column chromatography led to product degradation. For this reason, after acid-base work-up to remove the 4-methoxybenzaldehyde byproduct, the crude 2-aminomethyl-4-phenyl-1-azabicyclo[2.2.1]heptane **8** was used as such in the next step. To chemically link this constrained piperidine scaffold with a quinoline nucleus, two different coupling reactions were deployed, resulting in two different sets of quinoline-piperidines (Scheme 1). First, reaction with 4,7-dichloroquinoline **9a** and 4-chloroquinoline **9b** under microwave irradiation, without the addition of solvent (‘neat’), produced 4-aminoquinolines **11a** and **11b**. Remarkably, performing the synthesis in the high-boiling solvent phenol, commonly applied for this type of amine-halide exchange reactions [37,38], resulted in a considerable side reaction of 4-chloroquinolines **9** with phenol, which rendered this procedure not useful. Purification by means of automated reversed phase column chromatography yielded 4-aminoquinolines **11a-b** in pure form (purity >95%, based on ^1^H NMR and LC-MS analysis), as a mixture of diastereomers. As can be seen in Scheme 1, the *endo* and *exo* forms eventually displayed the same reactivity, since the diastereomeric ratio did not change significantly after reaction (*dr* determined before purification). In order to expand our library of quinoline-piperidine analogues, a second series of new chloroquine analogues, i.e. (aminomethyl)quinolines (inspired by quinine **1** and mefloquine **3**), having an extra methylene linker between the quinoline core and the amino group, was developed, as this part of the antimalarial drug space is mostly untouched. Moreover, a recently reported 3-(aminomethyl)quinoline with promising activity against both a CQ-sensitive (IC_50_ = 4.73 μM) and CQ-resistant (IC_50_ = 15.25 μM) strain of *P. falciparum* [39], prompted us to combine our 1-azabicyclo[2.2.1]heptane with quinolines in this fashion. To that end, free amine **8** was subjected to reductive amination conditions using different commercially available quinoline carboxaldehydes **10a-e** and NaBH_4_ in methanol. After purification using automated reversed phase column chromatography, five novel (aminomethyl)quinolines **12a-e**, bearing a bridged piperidine side chain at either the 2-, 3-, or 4-position of the quinoline core (Scheme 1), were obtained in pure form (purity >95%, based on ^1^H NMR and LC-MS analysis). Also here, the reaction conditions applied did not affect the diastereomeric ratio (*dr* determined before purification). It must be emphasized that the reported yields in Scheme 1 for both 4-aminoquinolines **11** and (aminomethyl)quinolines **12** are calculated over two steps, starting from the *N*-protected 1-azabicylo[2.2.1]heptane **7**. The low yields are mainly attributed to the elaborate work-up procedure after CAN deprotection, which, after intensive extraction procedures, only afforded roughly 50% of crude primary amine **8**. The extended purification *en route* to analytically pure samples for bio-evaluation also contributed to the low yields.

**Scheme 2:**
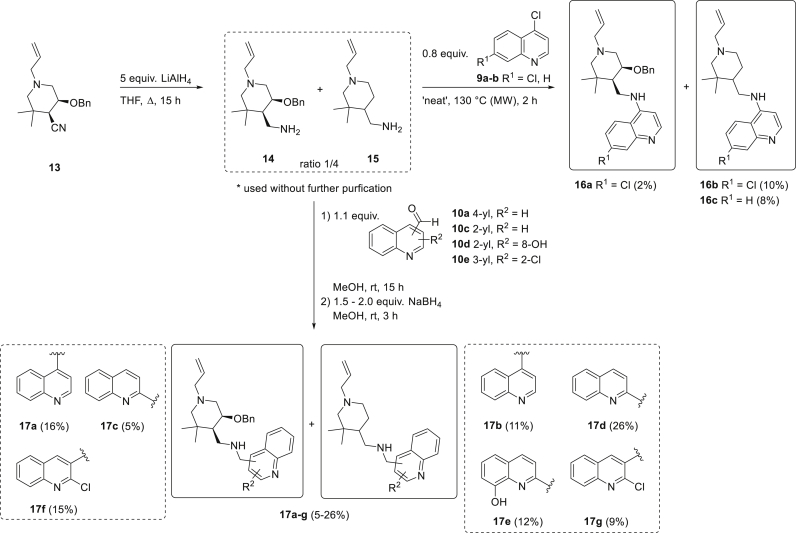
Synthetic route towards 4-aminoquinolines **16a-c** and (aminomethyl)quinolines **17a-g** functionalized with a monocyclic piperidine side chain.

**Fig. 2:**
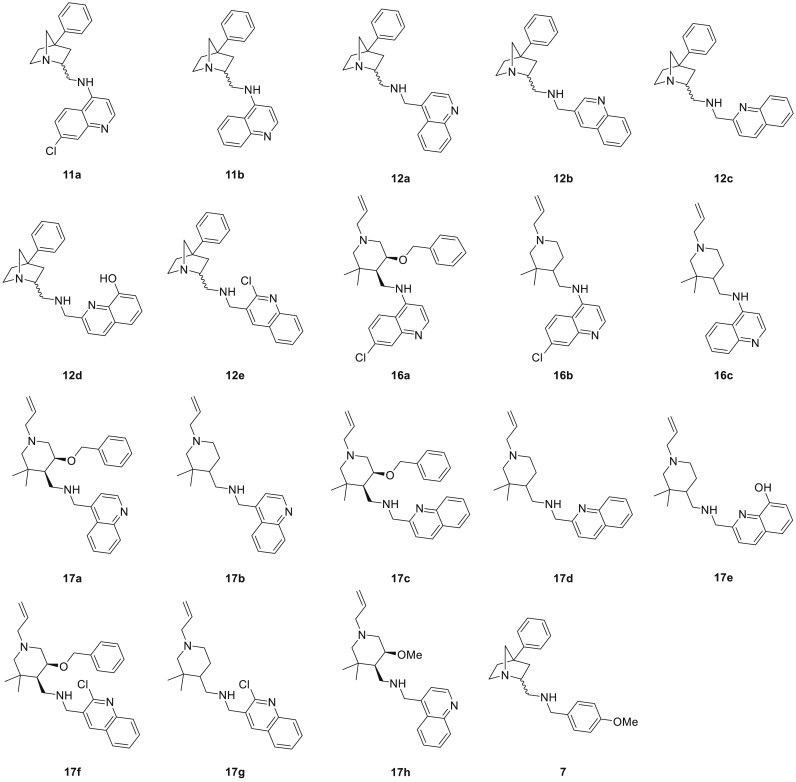
Overview of novel 4-aminoquinoline-piperidines **11** and **16**, novel (aminomethyl)quinoline-piperidines **12** and **17**, and 1-azabicyclo[2.2.1]heptane **7**, which were assayed on their antiplasmodium activity.

As a second heterocyclic structure, we proposed the introduction of a monocyclic piperidine ring in our desired set of quinoline-piperidine analogues, based on the structure of mefloquine **3**. Since our synthetic strategy required the presence of a functional group that could be converted into a primary amine, the *cis*-1-allyl-5-benzyloxy-3,3-dimethylpiperidine-4-carbonitrile **13** available in-house was selected as suitable and easily accessible monocyclic piperidine (Scheme 2). This class of piperidine-4-carbonitriles has not been previously screened for antiplasmodium activity, which allows for an interesting comparison with the 1-azabicyclo[2.2.1]heptane-quinolines **11** and **12**. The synthesis of piperidine-4-carbonitrile **13** is based on a 5-step literature procedure, involving the preparation of 4-(2-bromoalkyl)-β-lactams via the Staudinger reaction [40], their subsequent reduction to the corresponding azetidines and finally a ring transformation with potassium cyanide to obtain the desired piperidine-4-carbonitrile scaffold (Synthesis, see Supporting Information) [41]. Based on ^1^H NMR analysis of the intermediate *cis*-β-lactams, it was confirmed that piperidine-4-carbonitrile **13** also displayed the *cis*-configuration [41]. The introduction of an *N*-allyl group was envisaged to provide a handle for further synthetic modification later on, if desired, although other substituents (e.g. benzyl or *t*Bu) can also be easily incorporated. Reduction of the nitrile functionality with LiAlH_4_ yielded the corresponding 4-(aminomethyl)piperidine **14**, but also gave rise to a substantial amount of side product **15**, in which the benzyloxy moiety was cleaved off (Scheme 2). Although the removal of a methoxy group from a piperidine ring with LiAlH_4_ is known in the literature [42,43], this is the first reported example of a simultaneous nitrile reduction and benzyloxy group removal through the use of LiAlH_4_. Modification of the reaction parameters (adaptation of numbers of equivalents of LiAlH_4_, temperature and time) always resulted in a mixture with at least 50% of 5-unsubstituted piperidine **15**. Separation of the primary amines by means of reversed phase column chromatography only succeeded partially due to their tailing behaviour and was also accompanied by some product degradation. Therefore, we decided to use the mixture of primary amines **14** and **15** as such to react with an appropriate quinoline scaffold in the next step. To calculate the correct amount of reagents, an average molecular weight of the reaction mixture was used according to the ratio of both primary amines, which was approximately 1/4 (**14**/**15**) based on ^1^H NMR analysis. In order to compare the antiplasmodium activity of these monocyclic piperidine-quinoline series with the previously synthesized 1-azabicyclo[2.2.1]heptane-quinolines, the monocyclic piperidines were reacted with the same quinolines as in Scheme 1. Thus, 4-chloroquinolines **9a-b** were reacted with the mixture of primary amines **14** and **15** under microwave irradiation (‘neat’), which afforded a mixture of the corresponding 4-aminoquinolines **16** (Scheme 2). Only 0.8 equivalents of 4,7-dichloroquinoline **9a** or 4-chloroquinoline **9b** were used, since upon addition of an equimolar quantity, a significant amount of 4-chloroquinolines **9a-b** remained unreacted. By means of purification using automated reversed phase column chromatography, both piperidine-quinoline analogues **16a** and **16b** could be isolated in high purity in the case of R^1^ = Cl, whereas for R^1^ = H, only the analogue without the benzyloxy group (**16c**) was separated from the reaction mixture in high purity (purity >95%, based on ^1^H NMR and LC-MS analysis). Next to 4-aminoquinolines, a series of (aminomethyl)quinolines decorated with a monocyclic piperidine side chain was synthesized as well via reductive amination using four different quinoline carboxaldehydes **10a,c-e** (Scheme 2). In case no complete conversion was achieved after addition of 1.5 equivalents of NaBH_4_, 0.5 extra equivalents were added to reach a conversion of 100%. Table 1 gives an overview of (aminomethyl)quinolines **17a-g** that were isolated in a purity of >95% (based on ^1^H NMR and LC-MS analysis) after automated reversed phase column chromatography. Only derivative **17c** had a purity of >85% (based on ^1^H NMR and LC-MS analysis), but could still be characterized. In addition to these seven monocyclic piperidine-quinoline analogues **17a-g**, (aminomethyl)quinoline **17h** (see Fig. 2), containing a 5-methoxy-substituted piperidine structure, was synthesized in a similar fashion in a yield of 6%, by reaction of quinoline-4-carboxaldehyde **10a** and the mixture of *cis*-1-allyl-4-aminomethyl-5-methoxy-3,3-dimethylpiperidine and 1-allyl-4-aminomethyl-3,3-dimethylpiperidine **15**. Since the intermediate mixture of primary amines **14** and **15** was not purified, the yields for all novel 4-aminoquinolines **16a-c** and aminomethylquinolines **17a-h**, functionalized with a monocyclic piperidine side chain, were calculated over two steps, starting from the corresponding piperidine-4-carbonitrile **13**. Also, novel analogues bearing a methoxy or benzyloxy group (**16a**, **17a**, **17c**, **17f**, **17h**) featured the *cis*-configuration, since no diastereomerisation was observed via ^1^H NMR analysis during all reactions.

**Table 1:** Specific substitution pattern and yields of all piperidine-(aminomethyl)quinolines **17a-g**, functionalized with a monocyclic piperidine side chain, that were obtained in pure form.

Compound	OBn?	Aldehyde **10**	Equiv. NaBH_4_	Yield (%)
**17a**	Yes	**10a**	1.5	16
**17b**	No	**10a**	1.5 + 0.5	11
**17c**	Yes	**10c**	1.5 + 0.5	5^a^
**17d**	No	**10c**	1.5 + 0.5	26
**17e**	No	**10d**	1.5	12
**17f**	Yes	**10e**	1.5 + 0.5	15
**17g**	No	**10e**	1.5 + 0.5	9

a Purity >85%, based on ^1^H NMR and LC-MS analysis.

### 2.2 Antiplasmodium activity assessments

An antiplasmodium assay was performed on this set of 18 novel quinoline-piperidine analogues to establish their potential as antimalarial agents. Fig. 2 shows an overview of the different compounds subjected to biological screening. All compounds were screened for *in vitro* antiplasmodium activity against a CQ-sensitive strain of *P. falciparum* (NF54). Subsequently, compounds showing promising antiplasmodium activity were also tested against a CQ-resistant strain of *P. falciparum* (K1) and analysed for *in vitro* cytotoxicity against a mammalian cell line, Chinese hamster ovarian (CHO) cells, using the 3-(4,5-dimethylthiazol-2-yl)-2,5-diphenyltetrazoliumbromide (MTT)-assay. Continuous *in vitro* cultures of asexual erythrocyte stages of *P. falciparum* were maintained using a modified method of Trager and Jensen [44], and quantitative assessment of antiplasmodium activity *in vitro* was determined via the parasite lactate dehydrogenase assay using a modified method described by Makler [45]. The test samples were tested in triplicate on one occasion. The MTT-assay was used as a colorimetric assay for cellular growth and survival, and compares well with other available assays [46,47]. The tetrazolium salt MTT was used to measure all growth and chemosensitivity. Again, samples were tested in triplicate on one occasion. Chloroquine was used as reference compound in both antiplasmodium screenings, while emetine was used as known antiprotozoal agent in the MTT-assay.

Table 2 summarizes the results of the biological tests. The IC_50_ values against NF54, K1 and CHO, accompanied by, if applicable, selectivity (SI) and resistance indices (RI) are shown. Since the compounds were not analysed all together but in three separate subsets, three IC_50_ values for chloroquine and emetine are reported.

**Table 2:** IC_50_ values of 4-aminoquinolines (**11** and **16**), (aminomethyl)quinolines (**12** and **17**) and 1-azabicyclo[2.2.1]heptane **7** tested for their *in vitro* antiplasmodium activity against a CQ-sensitive (NF54) and a CQ-resistant (K1) *P. falciparum* strain and their cytotoxicity. Data are expressed as mean ± SD values of three independent experiments.

Compound	NF54 IC_50_ (μM)	K1 IC_50_ (μM)	CHO IC_50_ (μM)	SI^a^	RI^b^
**11a**^d^	0.021 ± 0.0050	0.033 ± 0.0062	26.0 ± 2.9	1266	1.6
**11b**^e^	0.045 ± 0.007	0.069 ± 0.0113	11.2 ± 2.1	250	1.5
**12a**^c^	6.77 ± 0.25	–	–	–	–
**12b**^c^	4.07 ± 0.74	–	–	–	–
**12c**^c^	1.27 ± 0.04	–	–	–	–
**12d**^c^	0.72 ± 0.03	4.64 ± 0.13	102.65 ± 19.67	142	6.4
**12e**^c^	3.60 ± 0.31	–	–	–	–
**16a**^d^	0.012 ± 0.0010	0.026 ± 0.0057	121.8 ± 41.4	10,149	2.2
**16b**^d^	0.015 ± 0.0017	0.025 ± 0.0089	29.9 ± 1.2	1991	1.7
**16c**^e^	0.236 ± 0.032	0.0685 ± 0.0078	27.0 ± 3.2	114	0.3
**17a**^d^	2.37 ± 1.05	3.66 ± 0.43	139.4 ± 14.1	59	1.5
**17b**^d^	4.94 ± 1.18	–	–	–	
**17c**^d^	1.57 ± 0.17	2.82 ± 0.14	210.6 ± 31.4	134	1.8
**17d**^d^	3.66 ± 0.82	–	–	–	–
**17e**^d^	2.89 ± 1.06	–	–	–	–
**17f**^d^	1.28 ± 0.33	1.95 ± 0.078	199.0 ± 23.3	156	1.5
**17g**^d^	7.75 ± 1.44	–	–	–	–
**17h**^d^	17.99 ± 2.44	–	–	–	–
**7**^c^	1.55 ± 0.13	–	–	–	–
**Chloroquine**	0.011 ± 0.001^c^ 0.010 ± 0.0004^d^ 0.020 ± 0.001^e^	0.167 ± 0.028^c^ 0.169 ± 0.047^d^ 0.169 ± 0.047^e^	–	–	14.7 17.8 8.5
**Emetine**	–	–	0.04 ± 0.01^c^ 0.14 ± 0.04^d^ 0.03 ± 0.01^e^	–	–

-: not tested.

a SI (selectivity index) = IC_50_ CHO/IC_50_ NF54.

b RI (resistance index) = IC_50_ K1/IC_50_ NF54.

c Reference values: CQ: IC_50_ (NF54) 11.3 ± 1.0 nM, IC_50_ (K1) 166.5 ± 27.9 nM; emetine: IC_50_ 0.04 ± 0.01 μM.

d Reference values: CQ: IC_50_ (NF54) 9.5 ± 0.4 nM, IC_50_ (K1) 169.0 ± 46.7 nM; emetine: IC_50_ 0.14 ± 0.04 μM.

e Reference values: CQ: IC_50_ (NF54) 20.0 ± 1.4 nM, IC_50_ (K1) 169.0 ± 46.7 nM; emetine: IC_50_ 0.03 ± 0.01 μM.

The data obtained reveals that all piperidine-(aminomethyl)quinoline analogues **12** and **17** possess moderate activity against the CQ-sensitive strain of *P. falciparum* (NF54) with IC_50_ values below 8 μM, except for **17h**. However, the 4-aminoquinoline-piperidines **11** and **16** clearly stand out, exhibiting antiplasmodium activities in the low nanomolar range, comparable to chloroquine itself, with the exception of **16c**. Results in the CQ-resistant *P. falciparum* strain (K1) are in line with those observed in NF54. All 4-aminoquinoline-piperidines **11** and **16** demonstrate excellent inhibitory activity with nanomolar IC_50_ values in K1 (25–69 nM), 2.5- to 7-fold more potent than chloroquine. Their RI values are approximately 10 times lower compared to chloroquine. Peculiarly, 4-aminoquinoline-piperidine **16c** has a RI value below 1 (RI = 0.3). Although not unheard of, RI values lower than 1 are quite unusual, and cannot be explained straightforwardly. Nevertheless, these five 4-aminoquinoline-piperidines can be considered very promising potential novel antimalarial hit structures, showing the same intrinsic inhibitory activity as chloroquine (except for **16c**) in NF54 data, but able to circumvent the chloroquine resistance mechanisms. Additionally, data from the MTT-assay indicate no cytotoxicity issues for all 4-aminoquinoline-piperidines **11** and **16**, with SI values above 100.

A selective screening of the best performing (aminomethyl)quinolines (**12d**, **17a**, **17c** and **17f**) shows moderate antiplasmodium activity (IC_50_ 1.95–4.64 μM) in K1, comparable with that in NF54. These IC_50_ values in K1 are still higher than those of chloroquine, suggesting that this class of novel (aminomethyl)quinolines **12** and **17** exert their medium activity either much less effectively than chloroquine, or via another mechanism that does not significantly contribute to the antiplasmodium effect. Nevertheless, it is clear from these results that insertion of an extra methylene unit between the quinoline moiety and the amino functionality, as in (aminomethyl)quinoline-piperidines **12** and **17**, is detrimental to the antiplasmodium activity. Analysing the structures of the best performing compounds **11** and **16**, it seems that the presence of a chlorine atom on the 7-position of the quinoline nucleus delivers the best activity (analogues **11a** and **16a-b**). Conversely, omitting this chlorine atom (analogues **11b** and **16c**) lowers the activity only 2 to 3 times in the CQ-resistant strain, while its influence on activity in the CQ-sensitive strain varies, but still leads to nanomolar IC_50_ values in its absence. Thus, it appears that, with respect to the 4-amino-7-chloroquinoline nucleus present in chloroquine **2**, the 4-aminoquinoline moiety is the principal pharmacophore required for good activity, while the 7-chloro substitution only provides an additional effect. Furthermore, no substantial difference in antiplasmodium activity can be observed between bridged piperidine-quinolines **11** and monocyclic piperidine-quinolines **16**. In contrast, 1-azabicyclo[2.2.1]heptane **7** possesses moderate antiplasmodium activity, comparable to previous reported activities for this class [32], while their monocyclic counterparts were also tested and, as expected, displayed no antiplasmodium activity at all (data not shown). Thus, the antiplasmodium activity of the introduced piperidine side chain itself seems to exert little influence on the final activity of the 4-aminoquinoline-piperidines. From these findings, it seems unlikely that 1-azabicyclo[2.2.1]heptane-piperidines **11** are *hybrid structures* with a dual mode of action. Therefore, we assume that our novel 4-aminoquinoline-piperidines **11** and **16** bypass the chloroquine resistance mechanism and are not recognized by the mutated chloroquine transporters, or at least to a much lesser extent, and therefore can still exert their antiplasmodium activity by inhibition of heme crystallization. However, elaborate biological follow-up studies are necessary to confirm this hypothesis.

## 3 Conclusion

In this work, a library of 18 novel quinoline-piperidine conjugates was synthesized with the aim to investigate their antimalarial potential. To that end, two different piperidine moieties, a bicyclic 1-azabicyclo[2.2.1]heptane scaffold and a 4-substituted monocyclic piperidine, were selected and coupled with different quinoline cores either via an addition-elimination reaction or a reductive amination, affording in total four different series of quinoline-piperidines. Antiplasmodium evaluation against a CQ-sensitive (NF54) and a CQ-resistant (K1) strain of *P. falciparum* revealed a promising activity for almost all compounds. Amongst them, five novel analogues belonging to the class of 4-aminoquinoline-piperidines (**11a-b** and **16a-c**) demonstrated excellent *in vitro* antiplasmodium activity, with nanomolar IC_50_ values against both NF54 (12–236 nM) and K1 (25–69 nM) parasite strains. Furthermore, biological assessment in mammalian CHO cells did not indicate any cytotoxicity issues. Given the urgent need for novel antimalarial agents in the battle against this lethal parasitic disease and the emerging resistance to artemisinins, we propose our small library of five novel 4-aminoquinoline-piperidines **11a-b** and **16a-c** as promising hit structures for further hit-to-lead optimization towards new potent antimalaria medicines.

## 4 Experimental section

### 4.1 Synthesis and characterization

#### 4.1.1 General

All reagents were purchased from commercial suppliers and were used as received without any further purification. Dry solvents (THF, toluene, dichloromethane and diethyl ether) were obtained using the MBraun SPS-800 solvent purification system. Dry methanol was purchased from Acros Organics. Other solvents were purchased at the highest quality possible and used as supplied. Thin layer chromatography (TLC) analysis of crude reaction mixtures or pure samples was performed using glass-backed 0.25 mm Merck silica gel 60 F_254_ TLC plates, and visualised under UV light (254 nm) or by using a KMnO_4_ or ninhydrin stain. Purification by means of column chromatography was executed on chromatographic silica gel (particle size 35–70 μM, pore diameter 6 nm). Automated flash chromatography was carried out on a Grace Reveleris® X1 flash chromatography system (reversed phase) or Büchi Reveleris® X2 flash chromatography system (normal phase), using prepacked Reveleris® silica or Reveleris® C18 cartridges. ^1^H and ^13^C NMR spectra were recorded at 400 MHz and 100.6 MHz, respectively, on a Bruker Avance III, equipped with ^1^H/BB z-gradient probe (BBO, 5 mm). CDCl_3_ was used as solvent, and TMS was used as an internal chemical shift standard. All spectra were processed using TOPSPIN 3.6.2 and were acquired through the standard sequences available in the Bruker pulse program library. 2D spectra (COSY, HSQC, HMBC) were recorded as additive spectra for complete structure elucidation. IR spectra were obtained from samples in neat form with a Quest ATR (Attenuated Total Reflectance) accessory with diamond crystal puck using a Shimadzu IRAFFINITY-1S Fourier Transform Infrared Spectrophotometer (FTIR). Melting points of solid compounds were determined using a Kofler Bench, type WME Heizbank of Wagner & Munz. For HPLC analyses, an Agilent 1200 Series HPLC equipped with a Supelco Ascentic Express C18 column (3 cm × 4.6 mm, 2.7 μm fused-core particles, 90 Å), a Phenomenex Guard column (SecurityGuard Standard) and a UV-DAD detector was used. The HPLC is coupled to an Agilent 1100 Series MS with electrospray ionisation (70 eV) with a single quadrupole detector for HPLC-MS analyses. The latter one was also used for low resolution mass spectra (LRMS). High resolution electron spray (ES-TOF) mass spectra were obtained with an Agilent Technologies 6210 Series Time of Flight. Microwave reactions were performed in a CEM FocusedTM Microwave Synthesis System, Model Discover in 10 ml vials.

#### 4.1.2 Procedure for the synthesis of (4-phenyl-1-azabicyclo[2.2.1]heptan-2-yl)methanamine **8**

To a mixture of 10 ml acetonitrile and 40 ml water, 2-[*N*-(4-methoxybenzyl)aminomethyl]-4-phenyl-1-azabicyclo[2.2.1]heptane **7** [32] (322 mg, 1 mmol) and CAN (2.74 g, 5 mmol) were added to achieve a concentration of 0.1 M CAN in an acetonitrile/water mixture with a ratio of 1/4. The reaction mixture was stirred for 24 h at room temperature and checked afterwards for having reached acidic conditions (litmus paper). It was poured in 50 ml water and extracted with 50 ml dichloromethane. The dichloromethane phase was separated, while the emulsion-like aqueous phase was extracted in a second separation funnel containing 50 ml dichloromethane. After separation of the dichloromethane phase, the emulsion-like aqueous phase was extracted a last time in a third separation funnel with 50 ml dichloromethane, in order to remove as much as possible of the 4-methoxybenzaldehyde byproduct in the dichloromethane phase. Subsequently, the aqueous phase was basified with 1 N NaOH (litmus paper), and filtrated over Celite to remove the solids present. After filtration, the aqueous phase was extracted with dichloromethane in three different separation funnels (3 × 50 ml), like previously described. The combined organic phases were washed with 50 ml brine, dried over MgSO_4_, filtrated and evaporated under reduced pressure. The obtained crude (4-phenyl-1-azabicyclo[2.2.1]heptan-2-yl)methanamine **8** (94.2 mg) was used as such without purification in the next reactions.

#### 4.1.3 Procedure for the synthesis of a mixture of *cis*-1-allyl-4-aminomethyl-5-benzyloxy-3,3-dimethylpiperidine **14** and 1-allyl-4-aminomethyl-3,3-dimethylpiperidine **15**

A solution of *cis*-1-allyl-5-benzyloxy-3,3-dimethylpiperidine-4-carbonitrile **13** [41] (284 mg, 1 mmol) in 15 ml dry THF was cooled to 0 °C. A 1.0 M LiAlH_4_ solution in THF (5 ml, 5 mmol) was carefully added at 0 °C under argon atmosphere. Subsequently, the reaction was heated to reflux temperature and stirred for 15 h. Afterwards, the reaction mixture was cooled down to 0 °C again, after which the excess of LiAlH_4_ was quenched by carefully adding dropwise 5 ml water, followed by 5 ml 2 N NaOH. The salts in the mixture were removed via filtration, after which the solvent was evaporated. Then, 10 ml water was added and it was extracted with 3 × 20 ml dichloromethane, whereafter the combined organic phases were dried over MgSO_4_ and filtrated. Removal of the solvent under reduced pressure afforded a mixture of *cis*-1-allyl-4-aminomethyl-5-benzyloxy-3,3-dimethylpiperidine **14** and 1-allyl-4-aminomethyl-3,3-dimethylpiperidine **15** (228.6 mg), which was used without further purification in the next steps.

#### 4.1.4 Representative procedure for the synthesis of *N*-[(4-phenyl-1-azabicyclo[2.2.1]heptan-2-yl)methyl]quinolin-4-amines **11a-b**

For ease of calculations, it was assumed that the amount of crude starting material equals 1 equivalent. Primary amine (4-phenyl-1-azabicyclo[2.2.1]heptan-2-yl)methanamine **8** (94.2 mg, 0.47 mmol) and 4,7-dichloroquinoline **9a** (93 mg, 0.47 mmol) were put into a microwave vessel of 10 ml. The mixture was stirred under microwave irradiation for 2 h at 130 °C. After confirmation of full conversion via LC-MS analysis, the reaction product was dissolved in 50 ml dichloromethane, and if necessary 5 ml methanol was used to dissolve all precipitates. 5 ml 2 N NaOH and 10 ml water were added, followed by an extraction with dichloromethane (2 × 20 ml). The combined organic phases were washed with water (10 ml), dried over MgSO_4_, filtrated and evaporated under reduced pressure. Purification by means of automated column chromatography (C18, gradient MeOH/H_2_O 50/50 to 100/0) resulted in 45.2 mg 7-chloro-*N*-[(4-phenyl-1-azabicyclo[2.2.1]heptan-2-yl)methyl]quinolin-4-amine **11a** (0.124 mmol) in a purity of >95%, corresponding with a yield of 12% over two steps. The purification conditions for derivative **11b** are specified in the characterization.

##### 4.1.4.1 7-Chloro-*N*-[(4-phenyl-1-azabicyclo[2.2.1]heptan-2-yl)methyl]quinolin-4-amine 11a

Spectral data derived from the mixture of diastereomers (*dr* = 55/45).

^**1**^**H NMR** (400 MHz, CDCl_3_): δ 1.29 (1H, ddd, *J* = 11.7, 6.2, 1.4 Hz), 1.56 (1H, ddd, *J* = 11.9, 5.1, 3.6 Hz), 1.60–1.64 (1H, m), 1.72–1.78 (1H, m), 1.91–2.03 (3H, m), 2.19 (1H, ddd, *J* = 11.6, 10.6, 3.5 Hz), 2.66 (1H, dt, *J* = 9.8, 1.6 Hz), 2.79–2.87 (2H, m), 2.93–3.02 (5H, m), 3.13–3.32 (4H, m), 3.51–3.57 (1H, m), 3.72 (1H, hept, *J* = 5.4 Hz), 5.71 (1H, d, *J* = 4.1 Hz, NH_minor_), 5.97 (1H, d, *J* = 7.4 Hz, NH_major_), 6.37 (1H, d, *J* = 5.3 Hz), 6.41 (1H, d, *J* = 5.3 Hz), 7.24–7.39 (12H, m), 7.796 (1H, d, *J* = 9.0 Hz), 7.801 (1H, d, *J* = 8.9 Hz), 7.95 (1H, d, *J* = 2.1 Hz), 7.97 (1H, d, *J* = 2.1 Hz), 8.53 (1H, d, *J* = 5.3 Hz), 8.56 (1H, d, *J* = 5.3 Hz). ^**13**^**C NMR** (100 MHz, CDCl_3_): δ 37.6, 38.7, 42.3, 42.9, 44.5, 46.6, 47.1, 53.9, 55.0, 56.4, 59.8, 62.5, 64.87, 64.90, 99.1, 99.3, 117.1, 117.4, 121.4, 121.6, 125.2, 125.4, 126.6, 126.7, 126.8, 128.5, 128.76, 128.79, 134.8, 135.0, 141.8, 142.1, 149.2, 149.3, 149.4, 149.6, 152.1. **IR** (cm^−1^) ν_NH_ = 3294, *ν*_max_ = 2922, 2870, 1608, 1578, 1447, 1136, 806, 698. **MS** (70 eV): *m*/*z* (%) = 364/366 ([M+H]^+^, 100). **HRMS** (ESI) calcd for C_22_H_22_ClN_3_ [M+H]^+^ 364.1575, found 364.1577. Light yellow oil. Yield after automated column chromatography (C18, MeOH/H_2_O: 50/50 to 100/0) = 12%.

##### 4.1.4.2 *N*-[(4-Phenyl-1-azabicyclo[2.2.1]heptan-2-yl)methyl]quinolin-4-amine 11b

Spectral data derived from the mixture of diastereomers (*dr* = 60/40).

^**1**^**H NMR** (400 MHz, CDCl_3_): δ = 1.33 (1H, dd, *J* = 11.7, 5.4 Hz), 1.57 (1H, ddd, *J* = 11.9, 4.5, 4.5 Hz), 1.62–1.67 (1H, m), 1.72–1.79 (1H, m), 1.93–2.04 (3H, m), 2.22 (1H, ddd, *J* = 11.7, 11.2, 2.9 Hz), 2.67 (1H, d, *J* = 9.5 Hz), 2.81–2.89 (2H, m), 2.97–3.06 (5H, m), 3.16–3.35 (4H, m), 3.54–3.60 (1H, m), 3.74–3.82 (1H, m), 5.79 (1H, br s, NH_minor_), 6.00 (1H, d, *J* = 6.8 Hz, NH_major_), 6.40 (1H, d, *J* = 5.3 Hz), 6.43 (1H, d, *J* = 5.2 Hz), 7.24–7.37 (10H, m), 7.45 (2H, dd, *J* = 7.6, 7.5 Hz), 7.64 (2H, dd, *J* = 8.0, 7.5 Hz), 7.88 (1H, d, *J* = 7.6 Hz), 7.89 (1H, d, *J* = 7.6 Hz), 7.98 (1H, d, *J* = 8.0 Hz), 7.99 (1H, d, *J* = 8.0 Hz), 8.56 (1H, d, *J* = 5.3 Hz), 8.59 (1H, d, *J* = 5.2 Hz). ^**13**^**C NMR** (100 MHz, CDCl_3_): δ = 37.6, 38.6, 42.3, 43.0, 44.6, 46.5, 47.2, 53.8, 54.8, 56.4, 59.6, 62.4, 64.8, 64.9, 98.8, 99.0, 118.7, 119.0, 119.88, 119.94, 124.6, 124.7, 126.58, 126.63, 126.7, 126.8, 128.51, 128.55, 129.06, 129.12, 129.7, 129.8, 141.8, 142.0, 148.37, 148.42, 149.5, 149.7, 151.0. **IR** (cm^−1^) *ν*_max_ = 2951, 2172, 1580, 1572, 1128, 758, 725, 698. **MS** (70 eV): *m*/*z* (%) = 330 ([M+H]^+^, 100). **Mp** = 60 °C. Light yellow powder. Yield after automated column chromatography (C18, MeOH/H_2_O: 50/50 to 100/0) = 13%.

#### 4.1.5 Representative procedure for the synthesis of *N*-[(4-phenyl-1-azabicyclo[2.2.1]heptan-2-yl)methyl]-1-quinolinylmethanamines **12a-e**

For ease of calculations, it was assumed that the amount of crude starting material equals 1 equivalent. Crude (4-phenyl-1-azabicyclo[2.2.1]heptan-2-yl)methanamine **8** (94.2 mg, 0.47 mmol) was dissolved in 10 ml dry methanol and stirred under argon atmosphere. To this mixture, quinoline-4-carboxaldehyde **10a** (81.3 mg, 0.52 mmol) was added and subsequently stirred overnight at room temperature. After complete conversion, determined via LC-MS analysis, NaBH_4_ (26.7 mg, 0.71 mmol) was added portionwise and the reaction mixture was stirred for an additional 3 h at room temperature. When the reaction reached complete conversion, methanol was evaporated. The reaction mixture was resolved in 20 ml chloroform and 20 ml brine and extracted with chloroform (3 × 20 ml), after which the combined organic phases were dried over MgSO_4_, filtered and evaporated under reduced pressure. The resulting crude product was purified with automated column chromatography (C18, gradient MeOH/H_2_O 0/100 to 100/0). Finally, 20.5 mg *N*-[(4-phenyl-1-azabicyclo[2.2.1]heptan-2-yl)methyl]-1-(quinolin-4-yl)methanamine **12a** (0.060 mmol) was obtained in a purity of >95%, in a yield of 6% over two steps.

##### 4.1.5.1 *N*-[(4-Phenyl-1-azabicyclo[2.2.1]heptan-2-yl)methyl]-1-(quinolin-4-yl)methanamine 12a

Spectral data derived from the mixture of diastereomers (*dr* = 54/46).

^**1**^**H NMR** (400 MHz, CDCl_3_): δ 1.12 (1H, ddd, *J* = 11.4, 5.8, 2.0 Hz), 1.39 (1H, ddd, *J* = 11.7, 5.5, 3.6 Hz), 1.48–1.54 (1H, m), 1.64–1.71 (1H, m), 1.83–1.92 (3H, m), 2.07 (1H, ddd, *J* = 11.4, 10.7, 3.5 Hz), 2.32 (2 × 1H, br s, 2 × NH), 2.57–2.62 (2H, m), 2.70 (1H, dd, *J* = 11.8, 10.0 Hz), 2.75–2.81 (2H, m), 2.86–2.93 (6H, m), 3.01–3.07 (1H, m), 3.13 (1H, ddd, *J* = 11.8, 11.2, 5.9 Hz), 3.50–3.58 (1H, m), 4.26–4.37 (4H, m), 7.19–7.34 (10H, m), 7.51 (2H, d, *J* = 4.4 Hz), 7.55–7.59 (2H, m), 7.69–7.73 (2H, m), 8.09–8.14 (4H, m), 8.87 (2H, d, *J* = 4.4 Hz). ^**13**^**C NMR** (100 MHz, CDCl_3_): δ 37.5, 38.7, 42.6, 43.2, 46.8, 50.0, 50.2, 51.4, 53.5, 54.6, 54.9, 56.6, 60.1, 63.8, 65.0, 66.0, 119.7, 119.9, 123.3, 126.4, 126.47, 126.53, 126.7, 126.8, 127.0, 127.1, 128.39, 128.41, 129.0, 129.1, 130.18, 130.22, 142.3, 142.6, 145.5, 145.8, 148.2, 148.3, 150.37, 150.40. **IR** (cm^−1^) ν_NH_ = 3306, *ν*_max_ = 2949, 2884, 1593, 1570, 1508, 1497, 754, 698. **MS** (70 eV): *m*/*z* (%) = 344 ([M+H]^+^, 100). **HRMS** (ESI) calcd for C_23_H_25_N_3_ [M+H]^+^ 344.2121, found 344.2112. White-yellow oil. Yield after automated column chromatography (C18, gradient MeOH/H_2_O 0/100 to 100/0): 6%.

##### 4.1.5.2 *N*-[(4-Phenyl-1-azabicyclo[2.2.1]heptan-2-yl)methyl]-1-(quinolin-3-yl)methanamine 12b

Spectral data derived from the mixture of diastereomers (*dr* = 53/47).

^**1**^**H NMR** (400 MHz, CDCl_3_): δ 1.10 (1H, ddd, *J* = 11.6, 6.0, 1.6 Hz), 1.36 (1H, ddd, *J* = 11.7, 5.5, 3.6 Hz), 1.45–1.52 (1H, m), 1.63–1.69 (1H, m), 1.80–1.91 (3H, m), 2.05 (1H, ddd, *J* = 11.6, 10.6, 3.7 Hz), 2.13 (2 × 1H, br s, 2 × NH), 2.52 (1H, dd, *J* = 11.9, 4.3 Hz), 2.58–2.65 (2H, m), 2.73–2.94 (8H, m), 2.97–3.04 (1H, m), 3.12 (1H, ddd, *J* = 11.9, 11.1, 5.9 Hz), 3.46–3.54 (1H, m), 3.98–4.08 (4H, m), 7.19–7.33 (10H, m), 7.51–7.56 (2H, m), 7.66–7.71 (2H, m), 7.80 (2H, dd, *J* = 7.8, 3.6 Hz), 8.09–8.14 (4H, m), 8.92 (2H, dd, *J* = 5.0, 2.1 Hz). ^**13**^**C NMR** (100 MHz, CDCl_3_): δ 37.5, 38.8, 42.7, 43.2, 46.9, 51.0, 51.51, 51.55, 53.4, 54.5, 54.7, 56.6, 60.0, 63.8, 65.1, 66.1, 126.31, 126.33, 126.6, 126.68, 126.71, 126.8, 127.6, 128.03, 128.04, 128.4, 128.95, 129.01, 129.23, 129.25, 133.01, 133.08, 134.4, 134.5, 142.3, 142.7, 147.48, 147.53, 151.5, 151.6. **IR** (cm^−1^) ν_NH_ = 3285, *ν*_max_ = 2949, 2868, 1601, 1570, 1495, 1125, 756, 700. **MS** (70 eV): *m*/*z* (%) = 344 ([M+H]^+^, 100). **HRMS** (ESI) calcd for C_23_H_25_N_3_ [M+H]^+^ 344.2121, found 344.2126. Brown-yellow oil. Yield after automated column chromatography (C18, gradient MeOH/H_2_O 0/100 to 100/0): 6%.

##### 4.1.5.3 *N*-[(4-Phenyl-1-azabicyclo[2.2.1]heptan-2-yl)methyl]-1-(quinolin-2-yl)methanamine 12c

Spectral data derived from the mixture of diastereomers (*dr* = 55/45).

^**1**^**H NMR** (400 MHz, CDCl_3_): δ 1.13 (1H, ddd, *J* = 11.6, 6.0, 1.9 Hz), 1.40 (1H, ddd, *J* = 11.8, 5.1, 3.6 Hz), 1.45–1.52 (1H, m), 1.62–1.69 (1H, m), 1.80–1.91 (3H, m), 2.07 (1H, ddd, *J* = 11.6, 10.4, 3.7 Hz), 2.29 (2 × 1H, br s, 2 × NH), 2.53–2.61 (2H, m), 2.68–2.97 (8H, m), 3.02–3.10 (2H, m), 3.11–3.17 (1H, m), 3.47–3.57 (1H, m), 4.13–4.22 (4H, m), 7.19–7.33 (10H, m), 7.49–7.54 (4H, m), 7.66–7.72 (2H, m), 7.78–7.81 (2H, m), 8.07 (2H, d, *J* = 8.4 Hz), 8.12 (2H, d, *J* = 8.4 Hz). ^**13**^**C NMR** (100 MHz, CDCl_3_): δ 37.5, 38.8, 42.8, 43.2, 47.0, 47.8, 51.4, 53.5, 54.7, 55.96, 56.04, 56.6, 60.1, 64.0, 65.2, 66.1, 120.4, 120.5, 126.00, 126.04, 126.3, 126.7, 126.8, 127.3, 127.6, 128.4, 129.0, 129.37, 129.44, 136.4, 142.9, 147.7, 160.5, 160.6. **IR** (cm^−1^) ν_NH_ = 3323, *ν*_max_ = 2951, 2870, 1599, 1425, 1111, 829, 756, 698. **MS** (70 eV): *m*/*z* (%) = 344 ([M+H]^+^, 100). **HRMS** (ESI) calcd for C_23_H_25_N_3_ [M+H]^+^ 344.2121, found 344.2109. Brown-yellow oil. Yield after automated column chromatography (C18, gradient MeOH/H_2_O 0/100 to 100/0): 4%.

##### 4.1.5.4 2-{*N*-[(4-Phenyl-1-azabicyclo[2.2.1]heptan-2-yl)methyl]aminomethyl}quinolin-8-ol 12d

Spectral data derived from the mixture of diastereomers (*dr* = 54/46).

^**1**^**H NMR** (400 MHz, CDCl_3_): δ 1.10 (1H, dd, *J* = 11.4, 6.0 Hz), 1.34 (1H, ddd, *J* = 11.6, 5.5, 3.6 Hz), 1.46–1.52 (1H, m), 1.66–1.72 (1H, m), 1.81–1.93 (3H, m), 2.05 (1H, ddd, *J* = 11.4, 10.6, 3.5 Hz), 2.53–3.11 (12H, m), 3.17–3.24 (1H, m), 3.52–3.59 (1H, m), 4.05–4.22 (4H, m), 7.16–7.47 (18H, m), 8.08–8.11 (2H, m). ^**13**^**C NMR** (100 MHz, CDCl_3_): δ 37.5, 38.8, 42.6, 43.3, 46.8, 51.5, 53.1, 54.4, 54.6, 54.7, 55.3, 56.4, 60.1, 64.0, 65.0, 66.4, 110.5, 110.6, 117.5, 117.6, 120.9, 121.0, 126.3, 126.7, 126.8, 127.1, 127.2, 127.5, 127.6, 128.3, 128.4, 136.4, 136.5, 137.7, 142.3, 142.7, 152.4, 152.7, 157.7, 158.2. **IR** (cm^−1^) ν_NH_ = 3304, *ν*_max_ = 2932, 2870, 1599, 1505, 1466, 1242, 754, 698. **MS** (70 eV): *m*/*z* (%) = 360 ([M + H^+^], 100). **HRMS** (ESI) calcd for C_23_H_25_N_3_O [M+H]^+^ 360.2070, found 360.2057. Brown oil. Yield after automated column chromatography (C18, gradient MeOH/H_2_O 0/100 to 100/0): 3%.

##### 4.1.5.5 *N*-[(4-Phenyl-1-azabicyclo[2.2.1]heptan-2-yl)methyl]-1-(2-chloroquinolin-3-yl)methanamine 12e

Spectral data derived from the mixture of diastereomers (*dr* = 51/49).

^**1**^**H NMR** (400 MHz, CDCl_3_): δ 1.13 (1H, ddd, *J* = 11.5, 6.0, 1.7 Hz), 1.39 (1H, ddd, *J* = 11.6, 5.2, 3.7 Hz), 1.48–1.54 (1H, m), 1.65–1.71 (1H, m), 1.83–1.91 (3H, m), 2.08 (1H, ddd, *J* = 11.5, 10.5, 3.5 Hz), 2.29 (2 × 1H, br s, 2 × NH), 2.54–2.62 (2H, m), 2.66–2.71 (1H, m), 2.75–2.82 (2H, m), 2.85–2.98 (6H, m), 3.02–3.09 (1H, m), 3.15 (1H, ddd, *J* = 12.1, 11.2, 5.7 Hz), 3.51–3.59 (1H, m), 4.05–4.08 (4H, m), 7.20–7.33 (10H, m), 7.52–7.56 (2H, m), 7.66–7.71 (2H, m), 7.82 (2H, d, *J* = 8.0 Hz), 8.01 (2H, d, *J* = 8.4 Hz), 8.27 (1H, s), 8.28 (1H, s). ^**13**^**C NMR** (100 MHz, CDCl_3_): δ 37.5, 38.8, 42.6, 43.2, 46.8, 50.7, 50.9, 51.1, 53.5, 54.5, 54.6, 56.6, 60.0, 63.9, 65.1, 66.0, 126.4, 126.7, 126.8, 127.0, 127.1, 127.4, 128.2, 128.4, 129.9, 130.0, 131.8, 132.0, 137.0, 137.2, 142.3, 142.6, 146.7, 146.8, 150.5, 150.6. **IR** (cm^−1^) ν_NH_ = 3316, *ν*_max_ = 2936, 2870, 1593, 1491, 1327, 1032, 756, 700. **MS** (70 eV): *m*/*z* (%) = 378/380 ([M+H]^+^, 100). **HRMS** (ESI) calcd for C_23_H_24_ClN_3_ [M+H]^+^ 378.1732, found 378.1724. Brown-yellow oil. Yield after automated column chromatography (C18, gradient MeOH/H_2_O 0/100 to 100/0): 5%.

#### 4.1.6 Representative procedure for the synthesis of *N*-[(1-allyl-3,3-dimethylpiperidin-4-yl)methyl]quinolin-4-amines **16a-c**

The crude mixture of *cis*-1-allyl-4-aminomethyl-5-benzyloxy-3,3-dimethylpiperidine **14** and 1-allyl-4-aminomethyl-3,3-dimethylpiperidine **15** (101.8 mg, 0.50 mmol)^1^ was brought into a microwave vessel of 10 ml, and 4,7-dichloroquinoline **9a** (79.2 mg, 0.40 mmol) was added. The reaction mixture was stirred for 2 h at 130 °C under microwave irradiation. In case no complete conversion was reached, the product first had to be dissolved in methanol and evaporated again prior to further microwave irradiation, in order to avoid product degradation. After complete conversion, the reaction product was dissolved in 50 ml dichloromethane, and if necessary 5 ml methanol was used to dissolve all precipitates. 5 ml 2 N NaOH and 10 ml water were added, followed by an extraction with dichloromethane (2 × 20 ml). The combined organic phases were washed with water (10 ml), dried over MgSO_4_, filtrated and evaporated under reduced pressure. Purification by means of automated column chromatography (C18, gradient acetonitrile/H_2_O 30/70 to 100/0 with slow gradient of 1.4% per CV between 50 and 100% acetonitrile) afforded 3.2 mg *N*-[(*cis*-1-allyl-5-benzyloxy-3,3-dimethylpiperidin-4-yl)methyl]-7-chloroquinolin-4-amine **16a** (0.007 mmol) and 12.8 mg *N*-[(1-allyl-3,3-dimethylpiperidin-4-yl)methyl]-7-chloroquinolin-4-amine **16b** (0.037 mmol) in a purity of >95%, in a yield over two steps of 2% and 10% respectively. The purification conditions for *N*-[(1-allyl-3,3-dimethylpiperidin-4-yl)methyl]quinolin-4-amine **16c** is specified in the characterization.

##### 4.1.6.1 *N*-[(*cis*-1-Allyl-5-benzyloxy-3,3-dimethylpiperidin-4-yl)methyl]-7-chloroquinolin-4-amine 16a

^**1**^**H NMR** (400 MHz, CDCl_3_): δ 1.11 (3H, s), 1.18 (3H, s), 1.60–1.77 (1H, m), 1.96 (1H, d, *J* = 11.5 Hz), 2.13–2.32 (1H, m), 2.38 (1H, d, *J* = 11.5 Hz), 2.89–3.06 (1H, m), 3.03 (2H, d, *J* = 6.3 Hz), 3.42–3.48 (1H, m), 3.54–3.63 (1H, m), 3.76–3.85 (1H, m), 4.34 (1H, d, *J*_*AX*_ = 11.8 Hz), 4.79 (1H, d, *J*_*AX*_ = 11.8 Hz), 5.13–5.25 (2H, m), 5.84–5.95 (1H, m), 6.33 (1H, d, *J* = 5.4 Hz), 6.77 (1H, d, *J* = 9.0 Hz), 7.02 (1H, dd, *J* = 9.0, 1.8 Hz), 7.40–7.42 (5H, m), 7.87 (1H, d, *J* = 1.8 Hz), 8.45 (1H, d, *J* = 5.4 Hz). ^**13**^**C NMR** (100 MHz, CDCl_3_): δ 24.2, 28.4, 33.8, 39.8, 46.0, 54.0, 61.6, 71.0, 74.6, 98.3, 117.2, 117.6, 121.1, 124.9, 128.1, 128.3, 128.5, 128.8, 134.6, 135.3, 138.6, 142.5, 149.7, 151.9. **IR** (cm^−1^): *ν*_max_ = 2953, 1582, 1368, 914, 743. **MS** (70 eV): *m*/*z* (%) = 450/452 ([M+H]^+^, 100). **HRMS** (ESI) calcd for C_27_H_32_ClN_3_O [M+H]^+^ 450.2307, found 450.2324. Yellow oil. Yield after automated column chromatography (C18, gradient ACN/H_2_O 30/70 to 100/0 with slow gradient of 1.4% per CV between 50 and 100% acetonitrile): 2%.

Remark: The signal for C_quat_CH_2_N is not visible on ^13^C NMR.

##### 4.1.6.2 *N*-[(1-Allyl-3,3-dimethylpiperidin-4-yl)methyl]-7-chloroquinolin-4-amine 16b

^**1**^**H NMR** (400 MHz, CDCl_3_): δ 1.04 (3H, s), 1.05 (3H, s), 1.43–1.51 (1H, m), 1.52–1.63 (1H, m), 1.69 (1H, d, *J* = 11.3 Hz), 1.73–1.79 (1H, m), 1.88 (1H, ddd, *J* = 11.6, 11.6, 2.8 Hz), 2.50 (1H, dd, *J* = 11.3, 1.5 Hz), 2.85 (1H, dd, *J* = 13.5, 6.8 Hz), 2.90–3.05 (3H, m), 3.50–3.57 (1H, m), 4.98 (1H, br s, NH), 5.10–5.21 (2H, m), 5.78–5.89 (1H, m), 6.42 (1H, d, *J* = 5.3 Hz), 7.36 (1H, dd, *J* = 8.9, 1.9 Hz), 7.64 (1H, d, *J* = 8.9 Hz), 7.96 (1H, d, *J* = 1.9 Hz), 8.54 (1H, d, *J* = 5.3 Hz). ^**13**^**C NMR** (100 MHz, CDCl_3_): δ 20.3, 27.4, 27.5, 33.4, 44.34, 44.35, 54.5, 61.9, 67.4, 99.0, 117.19, 117.23, 120.7, 125.3, 129.0, 134.9, 135.6, 149.1, 149.8, 152.0. **IR** (cm^−1^): *ν*_max_ = 2951, 2868, 1611, 1452, 1368, 1136, 918. **MS** (70 eV): *m*/*z* (%) = 344/346 ([M+H]^+^, 100). **HRMS** (ESI) calcd for C_20_H_26_ClN_3_ [M+H]^+^ 344.1888, found 344.1891. **Mp** = 154 °C. White-yellow powder. Yield after automated column chromatography (C18, gradient ACN/H_2_O 30/70 to 100/0 with slow gradient of 1.4% per CV between 50 and 100% acetonitrile): 10%.

##### 4.1.6.3 *N*-[(1-Allyl-3,3-dimethylpiperidin-4-yl)methyl]quinolin-4-amine 16c

^**1**^**H NMR** (400 MHz, CDCl_3_): δ 1.04 (3H, s), 1.06 (3H, s), 1.44–1.52 (1H, m), 1.53–1.63 (1H, m), 1.69 (1H, d, *J* = 11.3 Hz), 1.77 (1H, dd, *J* = 12.9, 2.3 Hz), 1.88 (1H, ddd, *J* = 11.6, 11.5, 2.3 Hz), 2.50 (1H, d, *J* = 11.3 Hz), 2.84 (1H, dd, *J* = 13.4, 6.6 Hz), 2.93 (1H, br d, *J* = 11.5 Hz), 2.97–3.04 (2H, m), 3.55 (1H, dt, *J* = 12.7, 4.3 Hz), 4.99 (1H, br t, *J* = 4.3 Hz, NH), 5.11–5.19 (2H, m), 5.79–5.89 (1H, m), 6.43 (1H, d, *J* = 5.3 Hz), 7.42 (1H, dd, *J* = 7.9, 7.6 Hz), 7.63 (1H, dd, *J* = 8.1, 7.6 Hz), 7.71 (1H, d, *J* = 7.9 Hz), 7.98 (1H, d, *J* = 8.1 Hz), 8.56 (1H, d, *J* = 5.3 Hz). ^**13**^**C NMR** (100 MHz, CDCl_3_): δ 20.3, 27.4, 27.5, 33.4, 44.31, 44.34, 54.5, 61.8, 67.5, 98.7, 117.1, 118.8, 119.0, 124.6, 129.0, 130.2, 135.7, 148.5, 149.7, 151.1. **IR** (cm^−1^): ν_NH_ = 3325, *ν*_max_ = 2934, 1574, 1545, 1436, 1375, 764. **MS** (70 eV): *m*/*z* (%) = 310 ([M+H]^+^, 100), 156 (30). **Mp** = 134 °C. White-yellow powder. Yield after automated column chromatography (C18, gradient ACN/H_2_O 40/60 to 100/0 with slow gradient of 1.4% per CV): 8%.

#### 4.1.7 Representative procedure for the synthesis of *N*-[(1-allyl-3,3-dimethylpiperidin-4-yl)methyl]-1-quinolinylmethanamines **17a-h**

The crude mixture of *cis*-1-allyl-4-aminomethyl-5-benzyloxy-3,3-dimethylpiperidine **14** and 1-allyl-4-aminomethyl-3,3-dimethylpiperidine **15** (50.9 mg, 0.25 mmol),^2^,^3^ was dissolved in 10 ml dry methanol. Subsequently, 8-hydroxyquinoline-2-carboxaldehyde **10d** (47.6 mg, 0.275 mmol) was added under argon atmosphere and stirred for 15 h at room temperature. After confirmation of complete conversion by LC-MS analysis, NaBH_4_ (14.2 mg, 0.375 mmol) was added portionwise and stirring was continued for 3 h at room temperature. In case no complete conversion was reached after 3 h, an extra 0.5 equivalents of NaBH_4_ (4.7 mg, 0.125 mmol) were added. Methanol was evaporated, and the crude product was resolved in 10 ml dichloromethane, to which 10 ml brine was added. After a first extraction, the aqueous phase was extracted three times more with 10 ml dichloromethane. The combined organic phases were dried over MgSO_4_, filtrated and evaporated under reduced pressure. The obtained mixture of two *N*-[(piperidin-4-yl)methyl]-1-quinolinylmethanamines was purified by means of automated column chromatography (C18, gradient MeOH/H_2_O 30/70 to 100/0 with slow gradient of 0.625% per CV between 70 and 100% methanol), which resulted in 9.3 mg 2-{*N*-[(1-allyl-3,3-dimethylpiperidin-4-yl)methyl]aminomethyl}quinolin-8-ol **17e** (0.028 mmol) in a purity of >95%, and in a yield of 12% over two steps. The purification conditions for the other *N*-[(1-allyl-3,3-dimethylpiperidin-4-yl)methyl]-1-quinolinylmethanamines **17a-d,f-h** are specified in the characterization.

##### 4.1.7.1 *N*-[(*cis*-1-Allyl-5-benzyloxy-3,3-dimethylpiperidin-4-yl)methyl]-1-(quinolin-4-yl)methanamine 17a

^**1**^**H NMR** (400 MHz, CDCl_3_): δ 0.94 (3H, s), 1.09 (3H, s), 1.42–1.49 (1H, m), 1.84 (1H, d, *J* = 11.1 Hz), 1.98–2.10 (1H, m), 2.36 (1H, d, *J* = 11.1 Hz), 2.75 (1H, dd, *J* = 11.4, 3.4 Hz), 2.94–3.01 (1H, m), 2.98 (2H, dd, *J* = 6.3, 1.0 Hz), 3.05 (1H, dd, *J* = 11.4, 9.8 Hz), 3.79–3.83 (1H, m), 4.13 (1H, d, *J*_*AB*_ = 14.9 Hz), 4.19 (1H, d, *J*_*AB*_ = 14.9 Hz), 4.35 (1H, d, *J*_*AX*_ = 12.2 Hz), 4.68 (1H, d, *J*_*AX*_ = 12.2 Hz), 5.10–5.22 (2H, m), 5.82–5.93 (1H, m), 7.15–7.20 (1H, m), 7.23–7.27 (2H, m), 7.29–7.33 (3H, m), 7.49 (1H, ddd, *J* = 8.4, 7.0, 1.2 Hz), 7.68 (1H, ddd, *J* = 8.5, 7.0, 1.2 Hz), 7.99 (1H, dd, *J* = 8.4, 1.2 Hz), 8.10 (1H, dd, *J* = 8.5, 1.2 Hz), 8.73 (1H, d, *J* = 4.4 Hz). ^**13**^**C NMR** (100 MHz, CDCl_3_): δ 23.7, 28.5, 33.5, 45.8, 48.4, 50.4, 50.9, 55.0, 61.7, 70.9, 73.8, 117.3, 119.5, 123.2, 126.4, 127.0, 127.38, 127.41, 128.3, 129.0, 130.2, 135.6, 139.0, 145.4, 148.1, 150.4. **IR** (cm^−1^): *ν*_max_ = 2951, 2868, 2749, 1595, 1462, 1357, 1113, 1069, 756. **MS** (70 eV): *m*/*z* (%) = 430 ([M+H]^+^, 100). **HRMS** (ESI) calcd for C_28_H_35_N_3_O [M+H]^+^ 430.2853, found 430.2855. Yellow oil. Yield after automated column chromatography (C18, gradient MeOH/H_2_O 30/70 to 100/0 with slow gradient of 1.25% per CV between 70 and 100% MeOH): 16%.

Remark: One of the C_arom, quat_ signals is missing in ^13^C NMR.

##### 4.1.7.2 *N*-[(1-Allyl-3,3-dimethylpiperidin-4-yl)methyl]-1-(quinolin-4-yl)methanamine 17b

^**1**^**H NMR** (400 MHz, CDCl_3_): δ 0.905 (3H, s), 0.909 (3H,s), 1.20–1.28 (1H, m), 1.37–1.58 (1H, m), 1.63 (1H, d, *J* = 11.2 Hz), 1.80–1.91 (2H, m), 2.38–2.46 (2H, m), 2.82 (1H, dd, *J* = 13.6, 6.7 Hz), 2.89–2.95 (2H, m), 3.00 (1H, dd, *J* = 13.6, 5.7 Hz), 4.24 (1H, d, *J*_*AB*_ = 12.8 Hz), 4.29 (1H, d, *J*_*AB*_ = 12.8 Hz), 5.08–5.19 (2H, m), 5.78–5.89 (1H, m), 7.46 (1H, d, *J* = 4.4 Hz), 7.57 (1H, ddd, *J* = 8.4, 6.9, 1.5 Hz), 7.71 (1H, ddd, *J* = 8.2, 6.9, 1.4 Hz), 8.08 (1H, d, *J* = 8.4 Hz), 8.13 (1H, d, *J* = 8.2 Hz), 8.87 (1H, d, *J* = 4.4 Hz). ^**13**^**C NMR** (100 MHz, CDCl_3_): δ 20.4, 27.4, 27.6, 33.2, 45.5, 50.5, 50.9, 54.8, 62.0, 67.7, 117.0, 119.6, 123.3, 126.4, 127.1, 129.1, 130.2, 135.9, 145.9, 148.1, 150.4. **IR** (cm^−1^): *ν*_max_ = 2947, 2743, 1510, 1462, 916, 754. **MS** (70 eV): *m*/*z* (%) = 324 ([M+H]^+^, 100). **HRMS** (ESI) calcd for C_21_H_29_N_3_ [M+H]^+^ 324.2434, found 324.2434. Yellow oil. Yield after automated column chromatography (C18, gradient MeOH/H_2_O 30/70 to 100/0 with slow gradient of 1.25% per CV between 70 and 100% MeOH): 11%.

##### 4.1.7.3 *N*-[(*cis*-1-Allyl-5-benzyloxy-3,3-dimethylpiperidin-4-yl)methyl]-1-(quinolin-2-yl)methanamine 17c

Purity > 85% (based on ^1^H NMR analysis).

^**1**^**H NMR** (400 MHz, CDCl_3_): δ 0.91 (3H, s), 1.06 (3H, s), 1.41–1.50 (1H, m), 1.81 (1H, d, *J* = 11.2 Hz), 1.96–2.06 (1H, m), 2.32–2.45 (1H, m), 2.72 (1H, dd, *J* = 11.6, 3.4 Hz), 2.77–3.07 (4H, m), 3.82–3.86 (1H, m), 3.99 (1H, d, *J*_*AB*_ = 14.6 Hz), 4.06 (1H, d, *J*_*AB*_ = 14.6 Hz), 4.43 (1H, d, *J*_*AB*_ = 12.2 Hz), 4.68 (1H, d, *J*_*AB*_ = 12.2 Hz), 5.07–5.21 (2H, m), 5.79–5.92 (1H, m), 7.15–7.20 (1H, m), 7.23–7.28 (2H, m), 7.31–7.35 (2H, m), 7.38 (1H, d, *J* = 8.4 Hz), 7.49 (1H, ddd, *J* = 8.0, 7.0, 1.0 Hz), 7.67 (1H, ddd, *J* = 8.3, 7.0, 1.1 Hz), 7.76 (1H, dd, *J* = 8.0, 1.1 Hz), 8.00 (1H, d, *J* = 8.3 Hz), 8.02 (1H, d, *J* = 8.4 Hz). ^**13**^**C NMR** (100 MHz, CDCl_3_): δ 23.8, 28.4, 33.4, 45.4, 48.5, 50.6, 55.3, 56.2, 61.8, 71.0, 73.8, 117.1, 120.3, 120.5, 125.9, 127.2, 127.3, 127.4, 127.5, 128.2, 129.0, 129.3, 135.8, 136.3, 139.3, 147.7. **IR** (cm^−1^): *ν*_max_ = 2949, 2868, 1508, 1358, 1112. **MS** (70 eV): *m*/*z* (%) = 430 ([M+H]^+^, 100). Yellow oil. Yield after automated column chromatography (C18, gradient MeOH/H_2_O 30/70 to 100/0 with slow gradient of 0.625% per CV between 70 and 100% MeOH): 5%.

##### 4.1.7.4 *N*-[(1-Allyl-3,3-dimethylpiperidin-4-yl)methyl]-1-(quinolin-2-yl)methanamine 17d

^**1**^**H NMR** (400 MHz, CDCl_3_): δ 0.88 (3H, s), 0.89 (3H, s), 1.23–1.31 (1H, m), 1.41–1.53 (1H, m), 1.62 (1H, d, *J* = 11.2 Hz), 1.84–1.92 (2H, m), 2.38 (1H, dd, *J* = 11.4, 9.9 Hz), 2.43 (1H, dd, *J* = 11.2, 1.7 Hz), 2.81 (1H, dd, *J* = 13.7, 6.8 Hz), 2.86–2.94 (2H, m), 3.00 (1H, dd, *J* = 13.7, 5.8 Hz), 4.06 (1H, d, *J*_*AB*_ = 14.5 Hz), 4.12 (1H, d, *J*_*AB*_ = 14.5 Hz), 5.08–5.19 (2H, m), 5.78–5.89 (1H, m), 7.46 (1H, d, *J* = 8.4 Hz), 7.51 (1H, ddd, *J* = 8.1, 7.0, 1.1 Hz), 7.69 (1H, ddd, *J* = 8.4, 7.0, 1.1 Hz), 7.80 (1H, dd, *J* = 8.1, 1.1 Hz), 8.05 (1H, d, *J* = 8.4 Hz), 8.11 (1H, d, *J* = 8.4 Hz). ^**13**^**C NMR** (100 MHz, CDCl_3_): δ 20.4, 27.4, 27.5, 33.2, 45.4, 50.6, 54.9, 56.2, 62.1, 67.7, 116.9, 120.5, 126.0, 127.3, 127.6, 129.0, 129.4, 136.0, 136.4, 147.8, 160.6. **IR** (cm^–^^1^): *ν*_max_ = 2947, 2772, 2745, 1601, 1503, 1425, 1126, 916. **MS** (70 eV): *m*/*z* (%) = 324 ([M+H]^+^, 100). **HRMS** (ESI) calcd for C_21_H_29_N_3_ [M+H]^+^ 324.2434, found 324.2430. Yellow oil. Yield after automated column chromatography (C18, gradient MeOH/H_2_O 30/70 to 100/0 with slow gradient of 0.625% per CV between 70 and 100% MeOH): 26%.

##### 4.1.7.5 2-{*N*-[(1-Allyl-3,3-dimethylpiperidin-4-yl)methyl]aminomethyl}quinolin-8-ol 17e

^**1**^**H NMR** (400 MHz, CDCl_3_): δ 0.88 (3H, s), 0.89 (3H, s), 1.23–1.32 (1H, m), 1.42–1.55 (1H, m), 1.64 (1H, d, *J* = 11.2 Hz), 1.84–1.92 (2H, m), 2.35–2.47 (2H, m), 2.83 (1H, dd, *J* = 13.6, 6.8 Hz), 2.86–2.96 (2H, m), 3.01 (1H, dd, *J* = 13.6, 5.6 Hz), 4.08 (1H, d, *J*_*AB*_ = 15.1 Hz), 4.12 (1H, d, *J*_*AB*_ = 15.1 Hz), 5.08–5.19 (2H, m), 5.79–5.89 (1H, m), 7.16 (1H, d, *J* = 7.4 Hz), 7.31 (1H, d, *J* = 7.9 Hz), 7.39–7.44 (1H, m), 7.46 (1H, d, *J* = 8.4 Hz), 8.11 (1H, d, *J* = 8.4 Hz). ^**13**^**C NMR** (100 MHz, CDCl_3_): δ 20.4, 27.3, 27.5, 33.2, 45.4, 50.4, 54.8, 55.6, 62.0, 67.6, 110.1, 117.1, 117.7, 121.2, 127.1, 127.5, 135.8, 136.6, 137.5, 151.9, 158.1. **IR** (cm^−1^): *ν*_max_ = 2949, 2791, 2745, 1504, 1329, 1246, 833, 750. **MS** (70 eV): *m*/*z* (%) = 340 ([M+H]^+^, 100). **HRMS** (ESI) calcd for C_21_H_29_N_3_O [M+H]^+^ 340.2383, found 340.2381. Yellow oil. Yield after automated column chromatography (C18, gradient MeOH/H_2_O 30/70 to 100/0 with slow gradient of 0.625% per CV between 70 and 100% MeOH): 12%.

##### 4.1.7.6 *N*-[(*cis*-1-Allyl-5-benzyloxy-3,3-dimethylpiperidin-4-yl)methyl]-1-(2-chloroquinolin-3-yl)methanamine 17f

^**1**^**H NMR** (400 MHz, CDCl_3_): δ 0.93 (3H, s), 1.08 (3H, s), 1.43–1.51 (1H, m), 1.81–1.87 (1H, m), 1.96–2.15 (1H, m), 2.31–2.47 (1H, m), 2.68 (1H, dd, *J* = 11.6, 3.4 Hz), 2.94–3.05 (4H, m), 3.79–3.83 (1H, m), 3.87 (1H, d, *J*_*AB*_ = 15.3 Hz), 3.92 (1H, d, *J*_*AB*_ = 15.3 Hz), 4.40 (1H, d, *J* = 12.2 Hz), 4.70 (1H, d, *J* = 12.2 Hz), 5.09–5.23 (2H, m), 5.82–5.93 (1H, m), 7.10–7.15 (1H, m), 7.21–7.25 (2H, m), 7.31–7.35 (2H, m), 7.50–7.54 (1H, m), 7.66–7.71 (2H, m), 7.98–8.00 (1H, m), 8.01 (1H, s). ^**13**^**C NMR** (100 MHz, CDCl_3_): δ 23.7, 28.5, 33.5, 45.2, 48.4, 50.9, 51.1, 55.1, 61.8, 70.9, 73.5, 117.2, 127.0, 127.35, 127.41, 127.5, 127.8, 128.19, 128.24, 129.8, 132.1, 135.7, 136.9, 139.1, 146.7, 150.6. **IR** (cm^−1^): *ν*_max_ = 2951, 2868, 1460, 1327, 1030, 914. **MS** (70 eV): *m*/*z* (%) = 464/466 ([M+H]^+^, 100). Yellow oil. Yield after automated column chromatography (C18, gradient MeOH/H_2_O 30/70 to 100/0 with slow gradient of 0.625% per CV between 70 and 100% MeOH): 15%.

##### 4.1.7.7 *N*-[(1-Allyl-3,3-dimethylpiperidin-4-yl)methyl]-1-(2-chloroquinolin-3-yl)methanamine 17g

^**1**^**H NMR** (400 MHz, CDCl_3_): δ 0.89 (3H, s), 0.90 (3H, s), 1.21–1.30 (1H, m), 1.42–1.54 (1H, m), 1.64 (1H, d, *J* = 11.2 Hz), 1.82–1.93 (2H, m), 2.37 (1H, dd, *J* = 10.3, 10.1 Hz), 2.44 (1H, dd, *J* = 11.2, 1.6 Hz), 2.83 (1H, dd, *J* = 13.9, 7.6 Hz), 2.85–2.96 (2H, m), 3.01 (1H, dd, *J* = 13.9, 5.4 Hz), 3.98 (1H, d, *J*_*AB*_ = 15.2 Hz), 4.04 (1H, d, *J*_*AB*_ = 15.2 Hz), 5.08–5.19 (2H, m), 5.78–5.89 (1H, m), 7.56 (1H, ddd, *J* = 8.0, 7.0, 1.1 Hz), 7.71 (1H, ddd, *J* = 8.4, 7.0, 1.4 Hz), 7.82 (1H, d, *J* = 8.0 Hz), 8.01 (1H, d, *J* = 8.4 Hz), 8.20 (1H, s). ^**13**^**C NMR** (100 MHz, CDCl_3_): δ 20.4, 27.4, 27.5, 33.2, 45.5, 50.3, 51.2, 54.8, 62.0, 67.7, 117.0, 127.1, 127.36, 127.44, 128.2, 130.0, 132.1, 135.9, 137.2, 146.8, 150.6. **IR** (cm^−1^): *ν*_max_ = 2945, 2778, 1329, 1134, 1034, 914, 752. **MS** (70 eV): *m*/*z* (%): 358/360 ([M+H]^+^, 100), 446 (30). **HRMS** (ESI) calcd for C_21_H_28_ClN_3_ [M+H]^+^ 358.2045, found 358.2049. **Mp** = 50 °C. White-yellow powder. Yield after automated column chromatography (C18, gradient MeOH/H_2_O 30/70 to 100/0 with slow gradient of 0.625% per CV between 70 and 100% MeOH): 9%.

##### 4.1.7.8 *N*-[(*cis*-1-Allyl-5-methoxy-3,3-dimethylpiperidin-4-yl)methyl]-1-(quinolin-4-yl)methanamine 17h

^**1**^**H NMR** (400 MHz, CDCl_3_): δ 0.92 (3H, s), 1.01 (3H, s), 1.38–1.42 (1H, m), 1.78 (1H, d, *J* = 9.6 Hz), 1.97–2.03 (1H, m), 2.34 (1H, d, *J* = 9.6 Hz), 2.78 (1H, dd, *J* = 11.3, 4.1 Hz), 2.91–3.05 (4H, m), 3.31 (3H, s), 3.58 (1H, q, *J* = 3.6 Hz), 4.26 (1H, d, *J*_*AB*_ = 14.9 Hz), 4.28 (1H, d, *J*_*AB*_ = 14.9 Hz), 5.11–5.20 (2H, m), 5.84–5.94 (1H, m), 7.47 (1H, d, *J* = 4.4 Hz), 7.56 (1H, ddd, *J* = 8.7, 7.0, 1.0 Hz), 7.71 (1H, ddd, *J* = 8.4, 7.0, 1.1 Hz), 8.10 (1H, dd, *J* = 8.7, 1.1 Hz), 8.13 (1H, dd, *J* = 8.4, 1.0 Hz), 8.87 (1H, d, *J* = 4.4 Hz). ^**13**^**C NMR** (100 MHz, CDCl_3_): δ 23.6, 28.5, 33.4, 45.8, 48.4, 50.6, 54.7, 57.3, 61.9, 66.3, 76.1, 117.4, 119.8, 123.3, 126.4, 127.1, 129.1, 130.2, 135.5, 146.0, 148.2, 150.4. **IR** (cm^−1^): *ν*_max_ = 2928, 2868, 1593, 1508, 1462, 1364, 1111, 1082, 754. **MS** (70 eV): *m*/*z* (%) = 354 ([M+H]^+^, 100). **HRMS** (ESI) calcd for C_22_H_31_N_3_O [M+H]^+^ 354.2540, found 354.2538. Yellow oil. Yield after automated column chromatography (C18, gradient ACN/H_2_O 30/70 to 100/0): 6%.

### 4.2 Biological evaluation

#### 4.2.1 *In vitro* antiplasmodium assay

The test samples were prepared to a 20 mg/ml stock solution in 100% DMSO. Stock solutions were stored at −20 °C. Further dilutions were prepared in complete medium on the day of the experiment. Chloroquine (CQ) was used as the reference drug. A full dose-response was performed to determine the concentration inhibiting 50% of parasite growth (IC_50_ value). Test samples were tested at a starting concentration of 10 μg/ml (or 1 μg/ml), which was then serially diluted 2-fold in complete medium to give 10 concentrations, with the lowest concentration being 20 ng/ml (or 2 ng/ml). The same dilution technique was used for all samples. The highest concentration of solvent to which the parasites were exposed to had no measurable effect on the parasite viability (data not shown). The 50% inhibitory concentration (IC_50_) values were obtained from full dose-response curves, using a non-linear dose-response curve fitting analysis via GraphPad Prism v.4 software.

#### 4.2.2 *In vitro* assay for the evaluation of cytotoxic activity

The same stock solutions prepared for antiplasmodium testing were used for cytotoxicity testing. Test compounds were stored at −20 °C until use. Dilutions were prepared on the day of the experiment. Emetine was used as the reference drug in all experiments. The initial concentration of emetine was 100 μg/ml, which was serially diluted in complete medium with 10-fold dilutions to give 6 concentrations, the lowest being 0.001 μg/ml. The same dilution technique was applied to all the test samples. The highest concentration of solvent to which the cells were exposed to had no measurable effect on the cell viability (data not shown). The 50% inhibitory concentration (IC_50_) values were obtained from full dose-response curves, using a non-linear dose-response curve fitting analysis via GraphPad Prism v.4 software.

## Declaration of competing interest

The authors declare that they have no known competing financial interests or personal relationships that could have appeared to influence the work reported in this paper.

## Footnotes

1 The ratio of mixture **14**/**15** was 1/4, so an average MW of 203.5 g/mol was used for calculations.

2 The ratio of mixture **14**/**15** was 1/4, so an average MW of 203.5 g/mol was used for calculations.

3 For derivative **17h**, a mixture in a ratio of 1/2 was used, with an average MW of 192.3 g/mol.
