# Supplementary material for: Synthesis and biological evaluation of novel quinoline-piperidine scaffolds as antiplasmodium agents
Source: Eur J Med Chem. 2020 Jul 15;198:112330. doi: 10.1016/j.ejmech.2020.112330 (PMC7294232; doi:10.1016/j.ejmech.2020.112330)
Supplement: Multimedia component 1 [file mmc1.doc]

**Supporting Information**

**Synthesis and biological evaluation of novel quinoline-piperidine scaffolds as antiplasmodium agents**

Tim Van de Walle,^a^ Maya Boone,^a^ Julie Van Puyvelde,^a^ Jill Combrinck,^b, c^ Peter J. Smith,^b^ Kelly Chibale,^d^ Sven Mangelinckx,^a^ and Matthias D’hooghe*^a^

^a^ SynBioC Research Group, Department of Green Chemistry and Technology, Faculty of Bioscience Engineering, Ghent University, Coupure Links 653, B-9000 Ghent, Belgium
^b^ Division of Clinical Pharmacology, Department of Medicine, Medical School, University of Cape Town, K45, OMB, Groote Schuur Hospital, Observatory, 7925, South Africa
^c^ Wellcome Centre for Infectious Diseases Research in Africa, Institute of Infectious Disease and Molecular Medicine, South Africa
^d^ South African Medical Research Council Drug Discovery and Development Research Unit, Department of Chemistry and Institute of Infectious Disease & Molecular Medicine, University of Cape Town, Rondebosch 7701, South Africa

* Corresponding author. E-mail: [Matthias.Dhooghe@UGent.be](mailto:Matthias.Dhooghe@UGent.be)

Table of Contents

[1. Synthesis of 2-[*N*-(4-methoxybenzyl)aminomethyl]-4-phenyl-1-azabicyclo[2.2.1]heptane **7** 2](#__RefHeading___Toc32912494)

[2. Synthesis of *cis*-1-allyl-5-benzyloxy-3,3-dimethylpiperidine-4-carbontrile **13** and *cis*-1-allyl-5-methoxy-3,3-dimethylpiperidine-4-carbonitrile **S12** 4](#__RefHeading___Toc32912495)

[3. ^1^H and ^13^C NMR spectra of all newly synthesized compounds 6](#__RefHeading___Toc32912496)

[4. References 42](#__RefHeading___Toc32912497)

# Synthesis of 2-[*N*-(4-methoxybenzyl)aminomethyl]-4-phenyl-1-azabicyclo[2.2.1]heptane 7

To a stirred solution of 4-methoxybenzaldehyde **S1** (6.08 ml, 50 mmol) in 50 ml THF, allylamine (3.93 ml, 52.5 mmol) and MgSO_4_ (6.32 g, 52.5 mmol) were added at room temperature. The resulting mixture was stirred for one hour at reflux temperature. Filtration of the cooled reaction mixture and removal of the solvent under reduced pressure afforded 8.67 g *N*-[(4-methoxyphenyl)methylidene]-*N*-allylamine **S2** (49.5 mmol) in a purity of > 95% (based on ^1^H NMR analysis) [1].

To a stirred solution of *N*-[(4-methoxyphenyl)methylidene]-*N*-allylamine **S2** (8.67 g, 49.5 mmol) in 40 ml dry dichloromethane, a solution of bromine (2.53 ml, 49.5 mmol) in 10 ml dry dichloromethane was added slowly over 30 minutes at room temperature. After complete addition, the reaction mixture was stirred for an additional 30 minutes at room temperature. Removal of the solvent under reduced pressure yielded 16.42 g *N*-[(4-methoxyphenyl)methylidene]-2,3-dibromopropylamine **S3** (49.0 mmol) in a purity of >95% (based on ^1^H NMR analysis) [2].

Sodium borohydride (2.22 g, 58.8 mmol) was added in small portions to a solution of *N*-[(4-methoxyphenyl)methylidene]-2,3-dibromopropylamine **S3** (16.42 g, 49.0 mmol) in 50 ml methanol at 0 °C. The reaction mixture was subsequently heated to reflux temperature and stirred for two hours. After completion of the reaction, it was poured into water (50 ml) and extracted with ethyl acetate (3 × 50 ml). The combined organic phases were dried over MgSO_4_, filtrated and evaporated under reduced pressure, which yielded 12.43 g 2-bromomethyl-1-(4-methoxybenzyl)aziridine **S4** (48.5 mmol) in a purity of >95% (based on ^1^H NMR analysis) [1].

A solution of phenylacetonitrile (5.05 ml, 44 mmol) in 75 ml dry THF was cooled down to -84 °C, after which 2.0 M LDA in THF (22ml, 44 mmol) was added and stirred for 30 minutes at -84 °C. Then, a solution of 2-bromomethyl-1-(4-methoxybenzyl)aziridine **S4** (10.25 g, 40 mmol) in 40 ml dry THF was added via a syringe at -84 °C, after which the reaction mixture was heated to reflux temperature and stirred for 4.5 hours. Afterwards, the reaction mixture was poured into water (50 ml), extracted with diethyl ether (3 × 50 ml), dried over MgSO_4_, filtrated and evaporated under reduced pressure. Purification by means of column chromatography (silica, PE/EtOAc 3/1) afforded 7.72 g 2-(2-cyano-2-phenylethyl)-1-(4-methoxybenzyl)-aziridine **S5** (26.4 mmol) as a mixture of diastereomers (*dr* = 54/46) in a purity of >95% (based on ^1^H NMR analysis) [3].

To an ice-cooled solution of 2-(2-cyano-2-phenylethyl)-1-(4-methoxybenzyl)-aziridine **S5** (8.87 g, 25 mmol) in 40 ml dry THF, 2.0 M LDA in THF (15 ml, 30 mmol) was added and stirred for one hour at 0 °C. Then, 1-bromo-2-chloroethane (2.29 ml, 27.5 mmol) was added at 0 °C, after which the reaction mixture was heated to reflux temperature and stirred for an additional hour. When the reaction reached complete conversion, the reaction mixture was poured into a saturated NH_4_Cl solution (50 ml) and was extracted with diethyl ether (3 × 50 ml). The combined organic fractions were dried over MgSO_4_, filtrated and evaporated under reduced pressure. After purification by means of column chromatography (silica, PE/EtOAc 3/1), 7.28 g 2-(4-chloro-2-cyano-2-phenylbutyl)-1-(4-methoxybenzyl)aziridine **S6** (20.5 mmol) was obtained as a mixture of diastereomers (*dr* = 52/48) in a purity of >95% (based on ^1^H NMR analysis).

**2-(4-Chloro-2-cyano-2-phenylbutyl)-1-(4-methoxybenzyl)aziridine S6**

Spectra derived from the mixture of diastereomers (*dr* = 52/48).

**^1^H NMR** (400 MHz, CDCl_3_): δ 1.32 (1H, d, *J* = 6.4 Hz), 1.38 (1H, d, *J* = 3.4 Hz), 1.39-1.45 (2H, m), 1.65-1.69 (1H, m), 1.71 (1H, d, *J* = 3.1 Hz), 2.01 (1H, dd, *J* = 13.9, 5.8 Hz), 2.02-2.04 (2H, m), 2.22 (1H, dd, *J* = 13.9, 5.4 Hz), 2.30 (1H, ddd, *J* = 13.9, 11.4, 5.2 Hz), 2.36 (1H, ddd, *J* = 13.9, 11.4, 5.1 Hz), 2.43 (1H, ddd, *J* = 13.9, 11.2, 5.4 Hz), 2.50 (1H, ddd, *J* = 13.9, 11.2, 5.3 Hz), 2.87 (1H, d, *J* = 12.9 Hz), 3.12 (1H, ddd, *J* = 11.2, 11.0, 5.2 Hz), 3.15 (1H, d, *J* = 12.9 Hz), 3.20 (1H, ddd, *J* = 11.2, 11.1, 5.1 Hz), 3.21 (1H, d, *J* = 12.6 Hz), 3.43 (1H, ddd, *J* = 11.4, 11.0, 5.4 Hz), 3.50 (1H, d, *J* = 12.6 Hz), 3.57 (1H, ddd, *J* = 11.4, 11.1, 5.3 Hz), 3.78 (3H, s), 3.80 (3H, s), 6.82 (2H, d, *J* = 8.6 Hz), 6.88 (2H, d, *J* = 8.6 Hz), 7.05 (2H, d, *J* = 8.6 Hz), 7.24 (2H, d, *J* = 8.6 Hz), 7.32-7.46 (10H, m). **^13^C NMR** (100 MHz, CDCl_3_): δ 32.9, 33.5, 34.9, 35.2, 39.3, 39.4, 42.1, 43.1, 44.1, 44.6, 46.0, 46.1, 55.3, 63.5, 64.0, 113.8, 114.0, 121.0, 121.3, 125.7, 126.0, 128.39, 128.44, 129.3, 129.4, 129.8, 130.6, 130.7, 136.3, 137.1, 158.8, 158.9. **MS** (70 eV): m/z (%) = 355 ([M+H]^+^, 100). Brown oil.

To a solution of 2-(4-chloro-2-cyano-2-phenylbutyl)-1-(4-methoxybenzyl)aziridine **S6** (7.01 g, 20 mmol, *dr* = 52/48) in 75 ml dry THF was added 1.0 M LiAlH_4_ in THF (40 ml, 40 mmol) at 0 °C, and the resulting mixture was stirred at reflux temperature for two hours. Afterwards, the reaction mixture was quenched carefully by adding 20 ml brine dropwise, after which it was filtrated over celite and evaporated under reduce pressure to remove the THF present. The aqueous residue was extracted with diethyl ether (3 × 50 ml), after which the combined organic fractions were washed with 50 ml brine. Drying over MgSO_4_, filtration and evaporation of the solvent under reduced pressure afforded 5.55 g 2-[*N*-(4-methoxybenzyl)aminomethyl]-4-phenyl-1-azabicyclo[2.2.1]heptane **7** (17.2 mmol) as a mixture of diastereomers (*dr* = 55/45) in a purity of > 95% (based on ^1^H NMR analysis).

**2-[*N*-(4-methoxybenzyl)aminomethyl]-4-phenyl-1-azabicyclo[2.2.1]heptane 7**

Spectra derived from the mixture of diastereomers (*dr* = 55/45).

**^1^H NMR** (400 MHz, CDCl_3_): δ 1.06-1.11 (1H, m), 1.33-1.38 (1H, m), 1.46-1.49 (1H, m), 1.62-1.68 (1H, m), 1.79-1.88 (3H, m), 2.03 (1H, ddd, *J* = 11.4, 10.6, 3.6 Hz), 2.45 (1H, dd, *J* = 11.6, 4.8 Hz), 2.54-2.59 (2H, m), 2.73-2.76 (4H, m), 2.82-2.89 (4H, m), 2.94-2.98 (1H, m), 3.10 (1H, td, *J* = 11.4, 5.2 Hz), 3.40-3.48 (1H, m), 3.76 (1H, d, *J* = 13.2 Hz), 3.77 (2H, s), 3.796 (3H, s), 3.805 (3H, s), 3.83 (1H, d, *J* = 13.2 Hz), 6.82-6.92 (4H, m), 7.20-7.33 (14H, m). **^13^C NMR** (100 MHz, CDCl_3_): δ 37.5, 38.7, 42.7, 43.2, 46.8, 50.7, 53.35, 53.37, 53.39, 54.3, 54.6, 55.3, 56.5, 60.0, 63.8, 65.0, 66.1, 113.78, 113.79, 114.6, 120.3, 126.3, 126.7, 126.8, 128.35, 128.37, 129.3, 129.4, 132.38, 132.40, 142.4, 142.8, 158.6, 158.7. **MS** (70 eV): m/z (%) = 323 ([M+H]^+^, 100). Light yellow oil.

The spectral data of the intermediate compounds **S2**, **S3**, **S4** and **S5** obtained during the synthetic pathway described above, are identical to the cited literature.

# Synthesis of *cis*-1-allyl-5-benzyloxy-3,3-dimethylpiperidine-4-carbonitrile 13 and *cis*-1-allyl-5-methoxy-3,3-dimethylpiperidine-4-carbonitrile S12

The synthesis of *cis*-1-allyl-5-benzyloxy-3,3-dimethylpiperidine-4-carbonitrile **13** will be described as a representative example.

A mixture of PCC (4.3 g, 20 mmol) and silica (4.3 g) was crushed in a mortar, after which it was dissolved in 40 ml dry dichloromethane. 3-Bromo-2,2-dimethylpropan-1-ol **S7** (1.23 ml, 10 mmol) was added and the reaction mixture was stirred at room temperature and under argon atmosphere for one hour. After completion of the reaction, 20 ml diethyl ether was added and the solution was filtered over celite. Removal of the solvent under reduced pressure, without evaporating the product itself (Bp (1 atm): 162 °C) resulted in 1.6 g 3-bromo-2,2-dimethylpropanal **S8** (9.7 mmol) in a purity of >95% (based on ^1^H NMR) [4].

To a mixture of 3-bromo-2,2-dimethylpropanal **S8** (1.6 g, 9.7 mmol) in 20 ml dry dichloromethane, MgSO_4_ (1.75 g, 14.55 mmol) and allylamine (0.76 ml, 10.19 mmol) were added, and the mixture was stirred at reflux temperature and under argon atmosphere for one hour. After filtration of MgSO_4_ and removal of the solvent under reduced pressure, 1.78 g *N*-allyl-3-bromo-2,2-dimethylpropan-1-imine **S9** (8.73 mmol) was obtained in a purity of >95% (based on ^1^H NMR) [5,6].

*N*-Allyl-3-bromo-2,2-dimethylpropan-1-imine **S9** (1.78 g, 8.73 mmol) was dissolved in 75 ml dry toluene and stirred at room temperature under argon atmosphere, after which Et_3_N (3.65 ml, 26.19 mmol) was added and the reaction mixture was heated to reflux temperature. A solution of benzyloxyacetyl chloride (1.79 ml, 11.35 mmol) in 25 ml dry toluene was added dropwise to this mixture, after which the final reaction mixture was stirred for an additional 30 minutes at reflux temperature, followed by stirring overnight at room temperature. After completion of the reaction, the reaction mixture was poured into 50 ml water and extracted with diethyl ether (3 × 30 ml). The combined organic fractions were dried over MgSO_4_, filtrated and evaporated under reduced pressure. After purification with column chromatography (silica, PE/EtOAc 6/1), 2.74 g *cis*-1-allyl-3-benzyloxy-4-[(2-bromo-1,1-dimethyl)ethyl]azetidin-2-one **S10a** (7.77 mmol) was obtained in a purity of >95% (based on ^1^H NMR) [7].

A solution of AlCl_3_ (2.67 g, 20 mmol) in 25 ml dry diethyl ether under argon atmosphere was cooled to 0 °C, to which 1.0 M LiAlH_4_ in THF (20 ml, 20 mmol) was added. The resulting solution was stirred at 0 °C for one hour, after which *cis*-1-allyl-3-benzyloxy-4-[(2-bromo-1,1-dimethyl)ethyl]azetidin-2-one **S10a** (1.76 g, 5 mmol) in 15 ml dry diethyl ether was added dropwise. The reaction mixture was heated to reflux temperature and stirred for an additional hour. Then, the reaction mixture was cooled down again to 0 °C and quenched carefully by adding 10 ml brine dropwise, and stirred overnight at room temperature. After decantation of the solvent, the aqueous residu was extracted with diethyl ether (5 × 10 ml) and dichloromethane (5 × 10 ml). The combined organic fractions were dried over MgSO_4_, filtrated and the solvent was evaporated under reduced pressure, which resulted in 1.30 g *cis*-1-allyl-3-benzyloxy-4-[(2-bromo-1,1-dimethyl)ethyl]azetidine **S11a** (3.85 mmol) in a purity of >95% (based on ^1^H NMR) [8].

***cis*-1-Allyl-4-[(2-bromo-1,1-dimethyl)ethyl]-3-methoxyazetidine S11b**

**^1^H NMR** (400 MHz, CDCl_3_): δ 1.11 (3H, s), 1.13 (3H, s), 2.84 (1H, dd, *J* = 9.0, 6.3 Hz), 2.89 (1H, dd, *J* = 13.3, 7.2 Hz), 3.20 (3H, s), 3.22 (1H, m), 3.34-3.43 (2H, m), 3.51 (1H, d, *J* = 9.8 Hz), 3.77 (1H, d, *J* = 9.8 Hz), 4.08 (1H, ddd, *J* = 6.5, 6.3, 2.6 Hz), .5.07-5.19 (2H, m), 5.75-5.85 (1H, m).

To a solution of *cis*-1-allyl-3-benzyloxy-4-[(2-bromo-1,1-dimethyl)ethyl]azetidine **S11a** (0.34 g, 1 mmol) in 20 ml DMSO, KCN (0.65 g, 10 mmol) was added, and the reaction mixture was stirred for one hour at reflux temperature. After complete conversion, the reaction mixture was poured into 20 ml water and extracted with diethyl ether (3 × 40 ml), after which the organic phases were washed with water (3 × 20 ml). After drying of the combined organic phases over MgSO_4_, filtration and evaporation of the solvent, 0.23 g *cis*-1-allyl-5-benzyloxy-3,3-dimethylpiperidine-4-carbonitrile **13** (0.81 mmol) was obtained in a purity of >95% (based on ^1^H NMR) [8].

***cis*-1-Allyl-5-methoxy-3,3-dimethylpiperidine-4-carbonitrile S12**

**^1^H NMR** (400 MHz, CDCl_3_): δ 1.13 (3H, s), 1.17 (3H, s), 2.12-2.15 (1H, m), 2.19-2.34 (2H, m), 2.81 (1H, d, *J* = 4.0 Hz), 2.97-3.07 (3H, m), 3.42 (3H, s), 3.61 (1H, dt, *J* = 9.5, 4.0 Hz), 5.13-5.21 (2H, m), 5.75-5.85 (1H, m).

The spectral data of the intermediate compounds **S8**, **S9**, **S10a-b**, **S11a** and end compound **13** obtained during the synthetic pathway described above, are identical to the cited literature.

# ^1^H and ^13^C NMR spectra of all newly synthesized compounds

**11a**


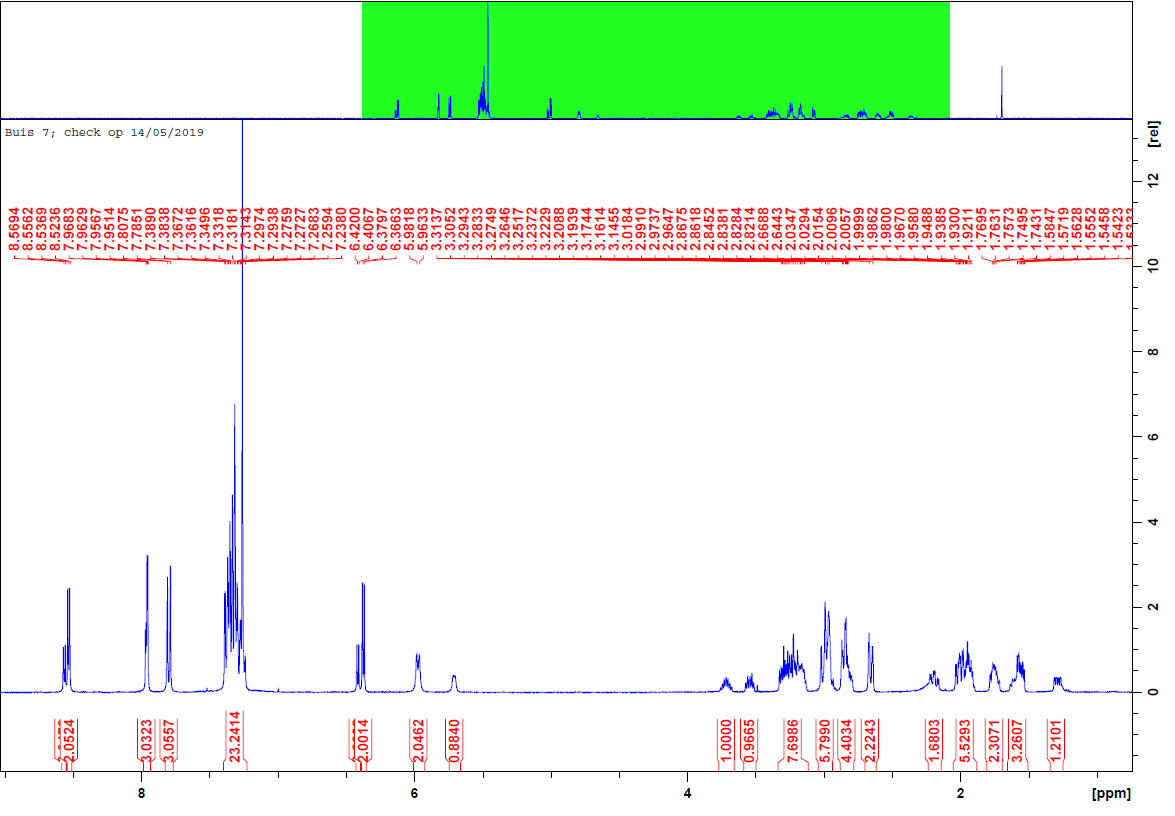


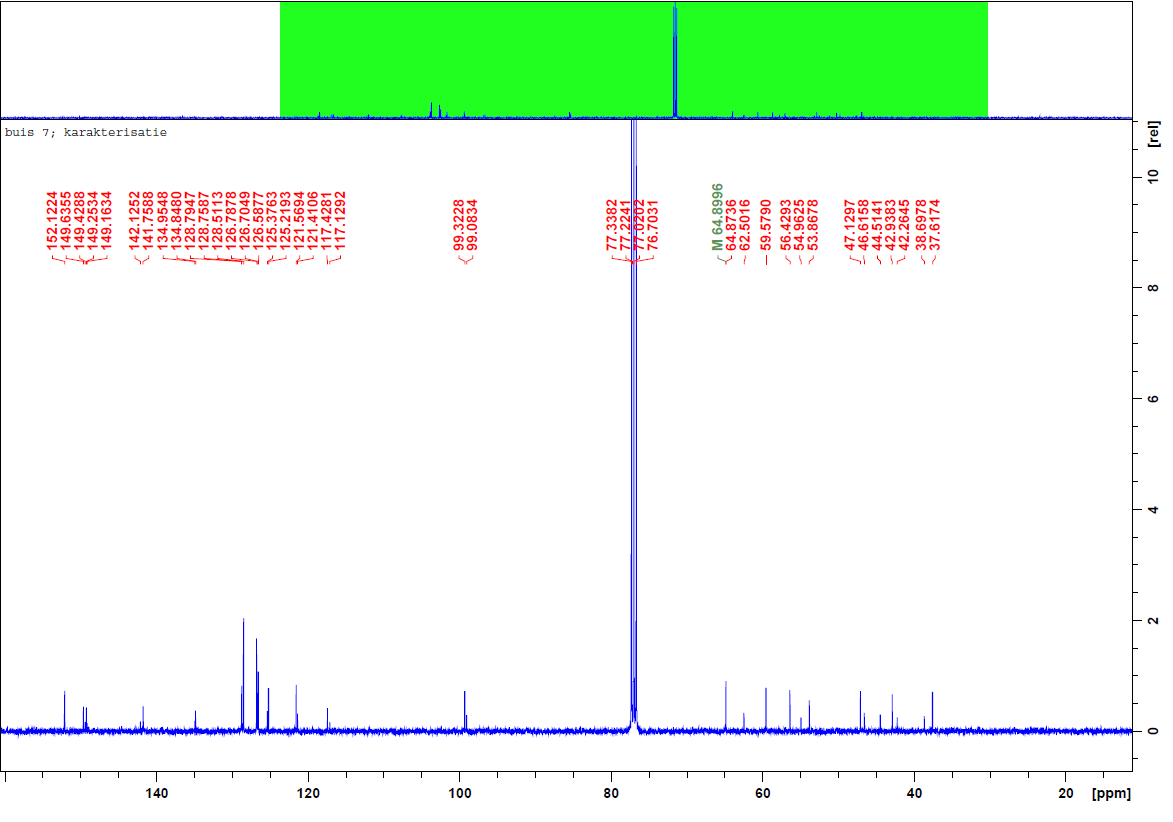


**11b**

**
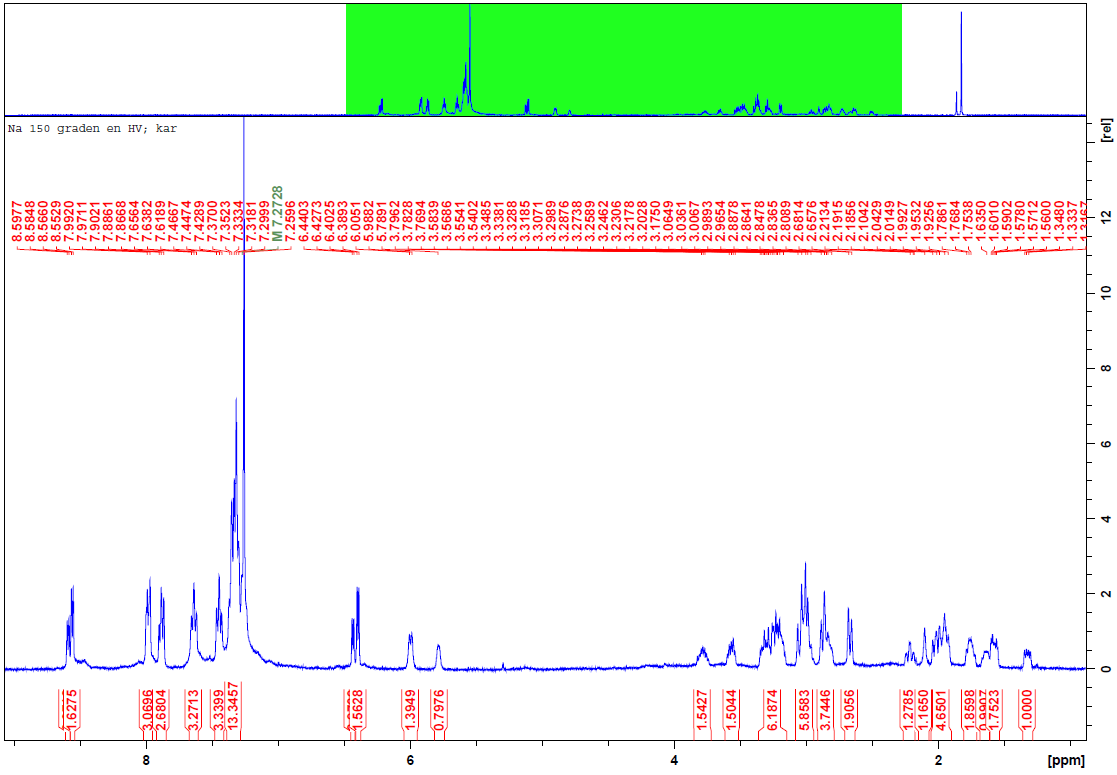
**

**
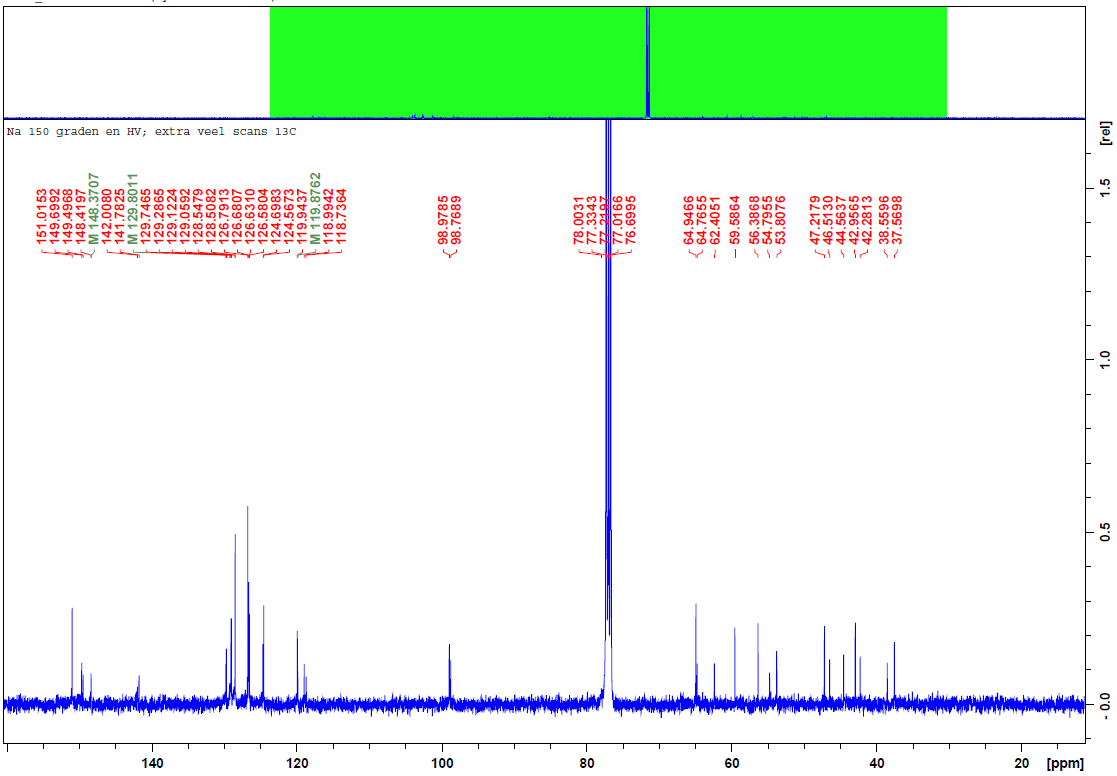
**

**12a**

**
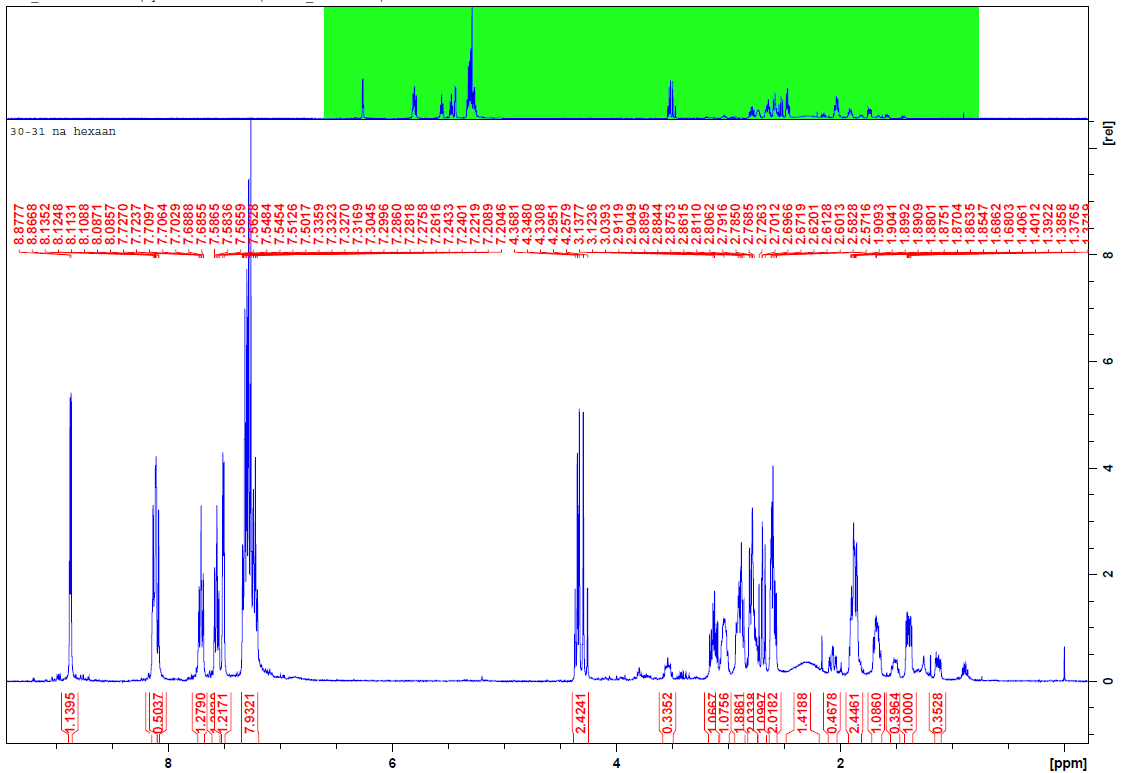
**

**
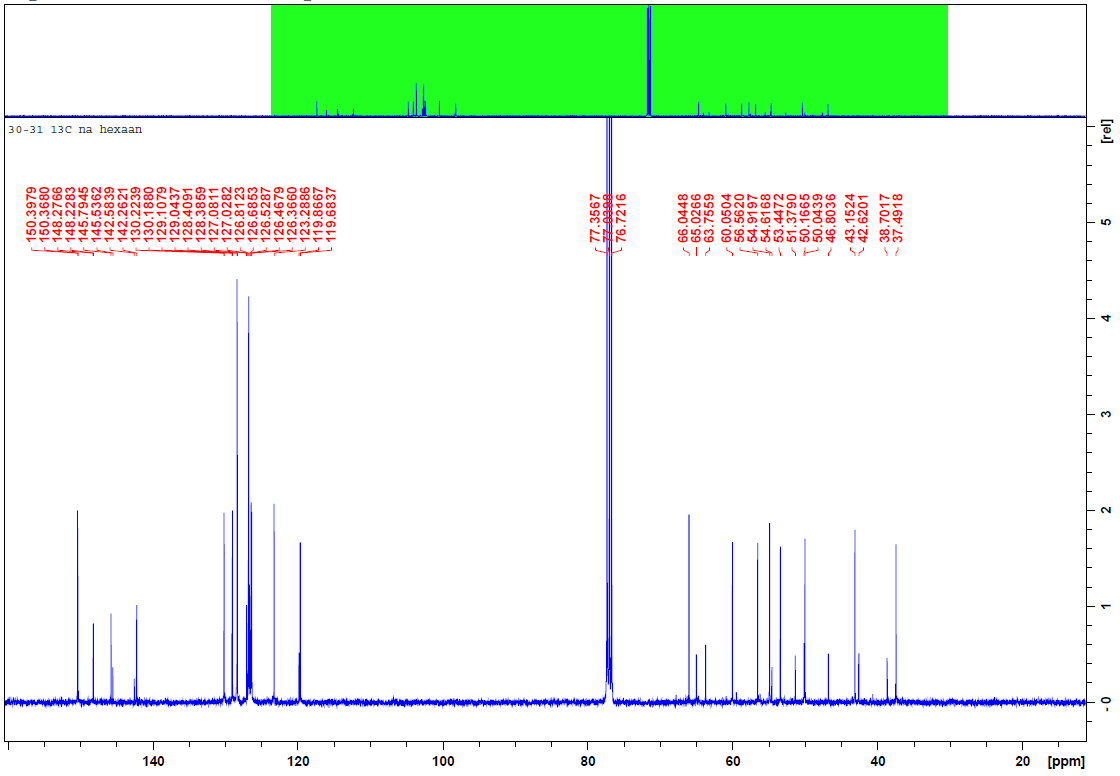
**

**12b**

**
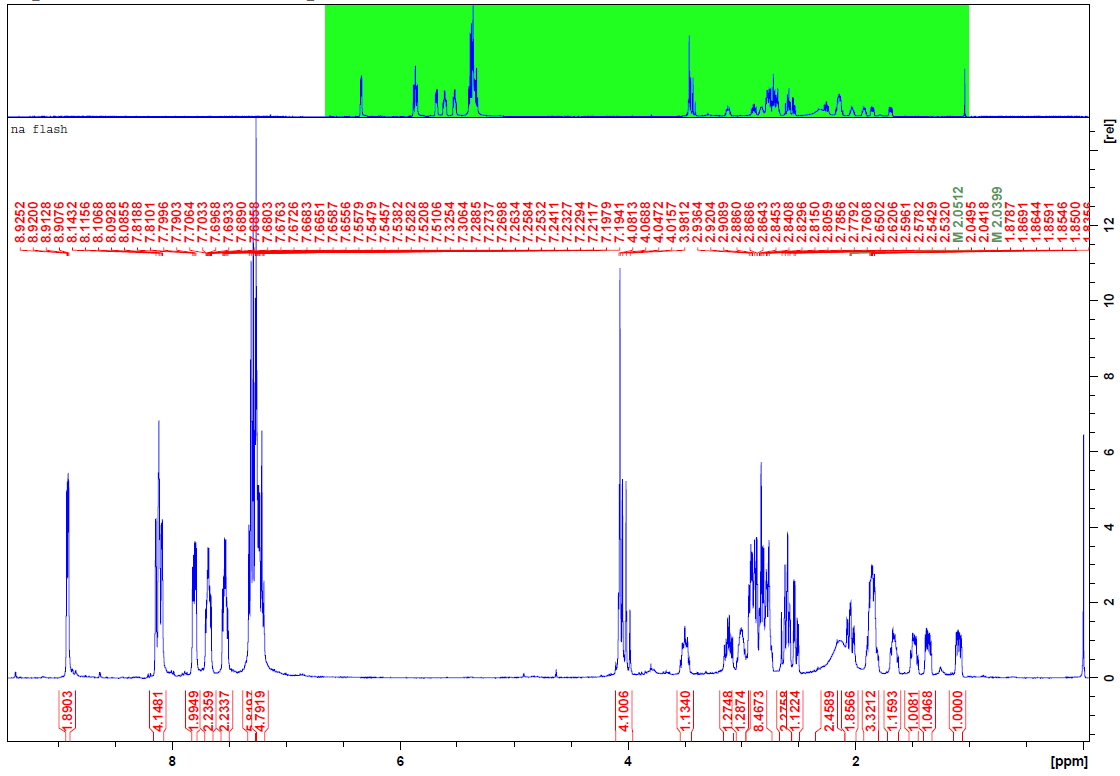
**

**
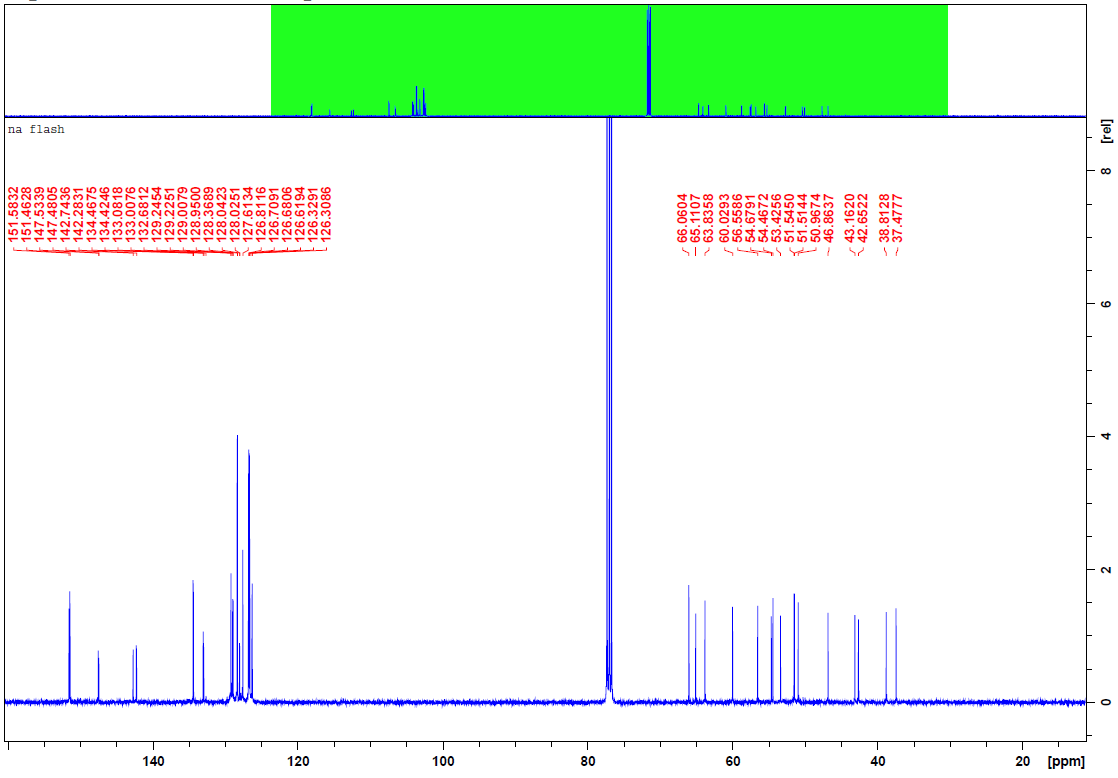
**

**12c**

**
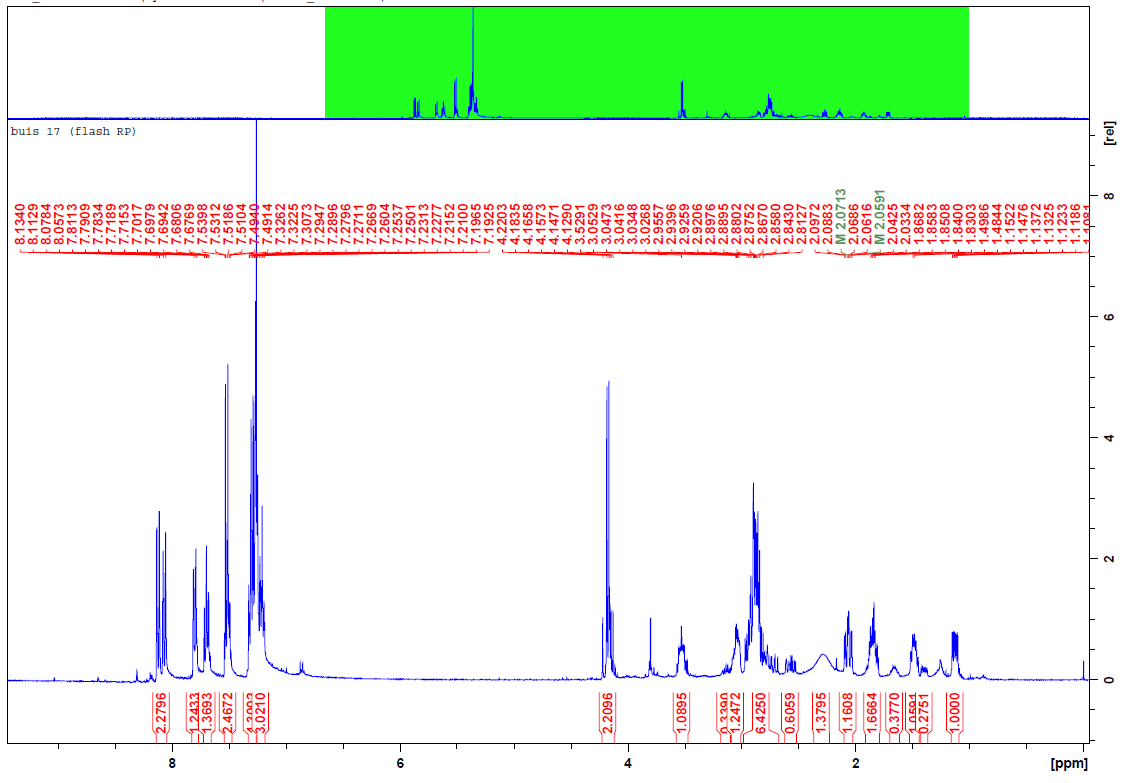
**

**
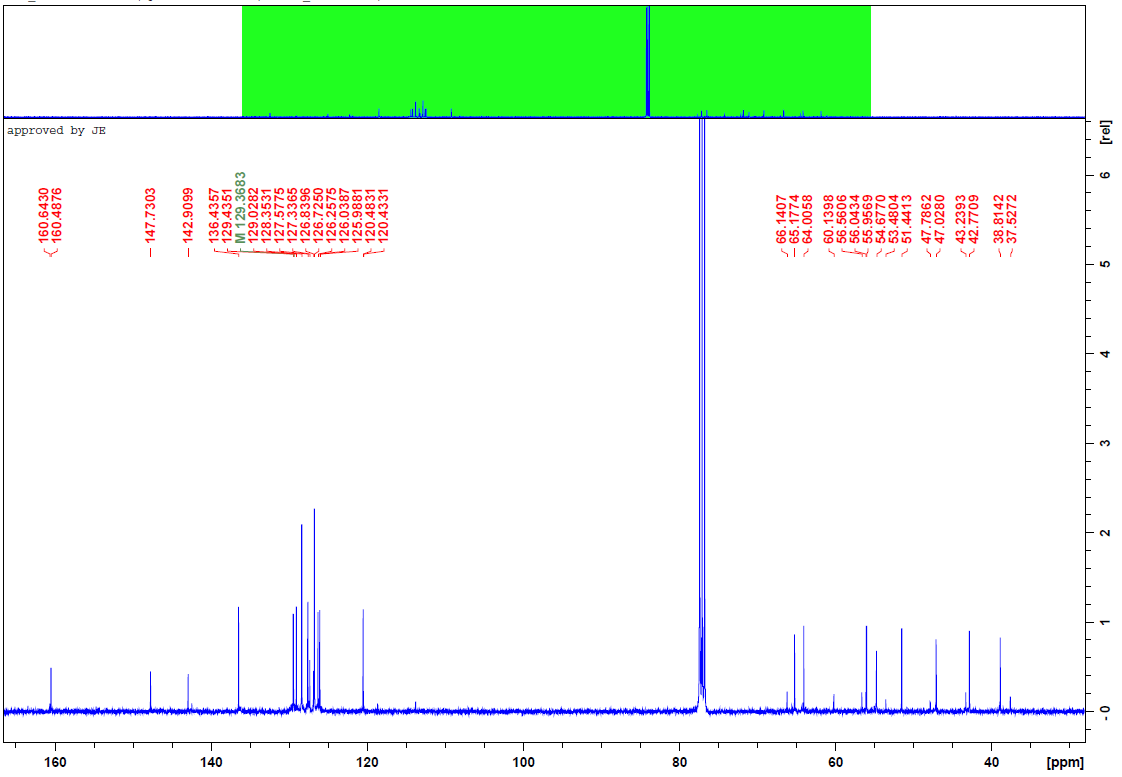
**

**12d**

**
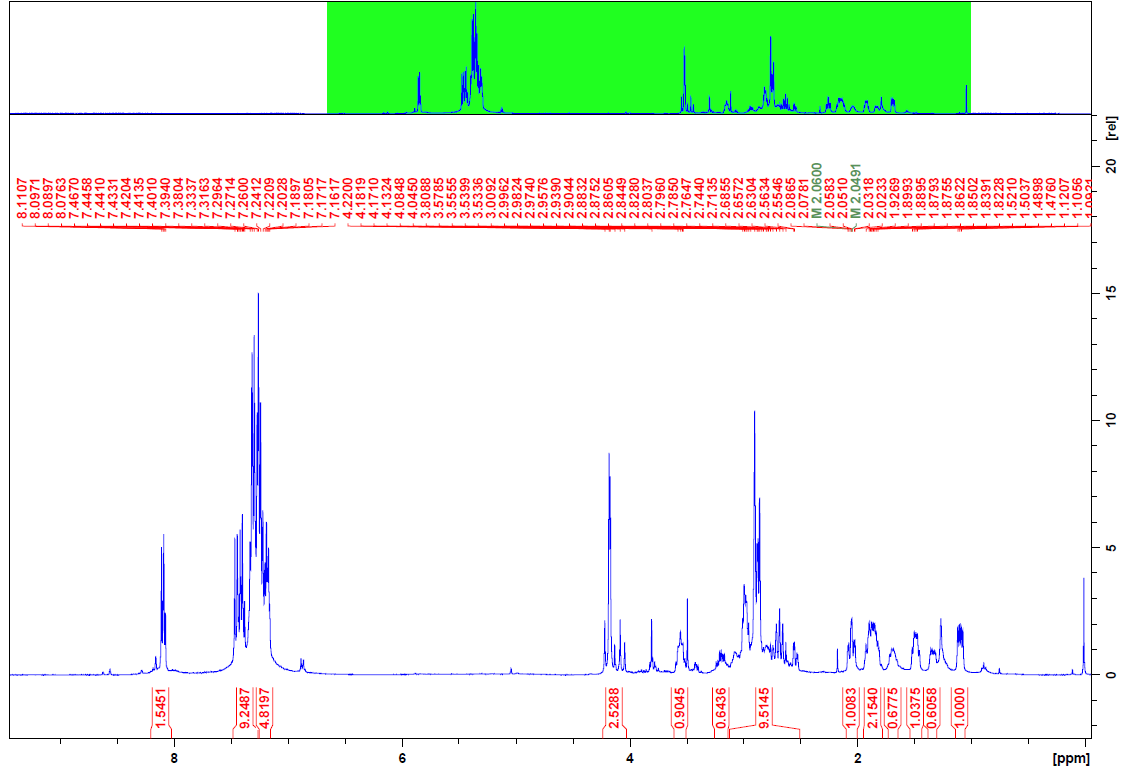
**

**
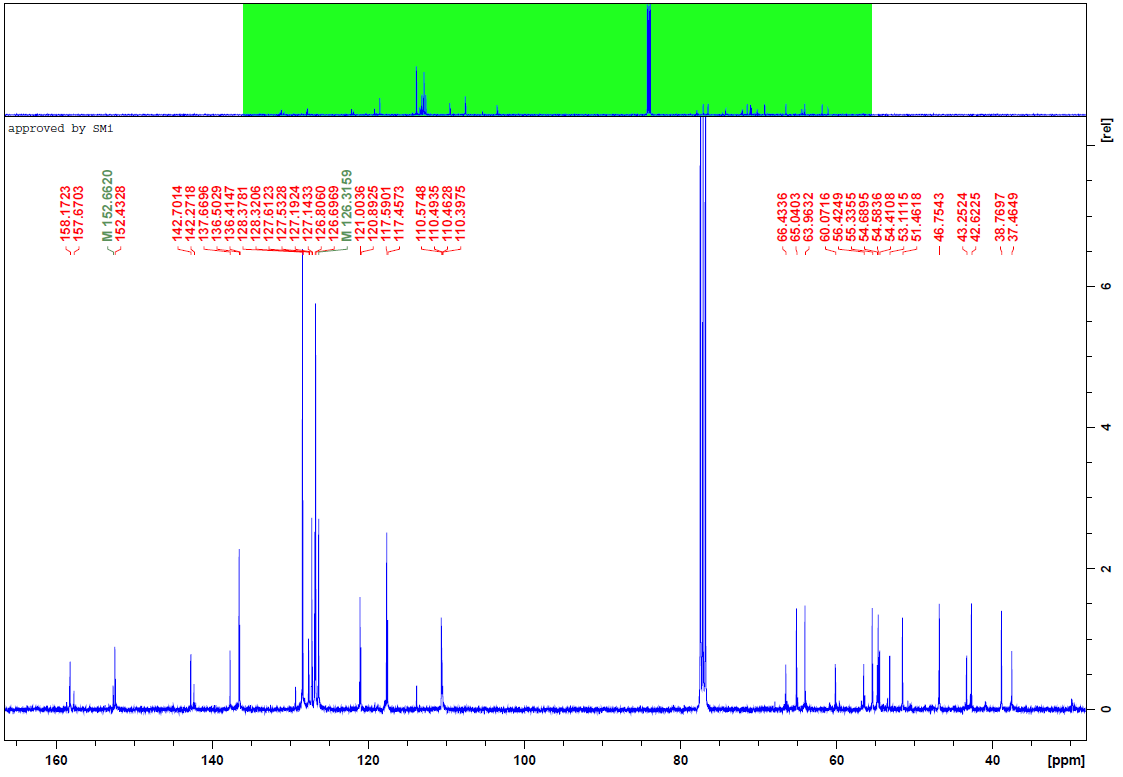
**

**12e**

**
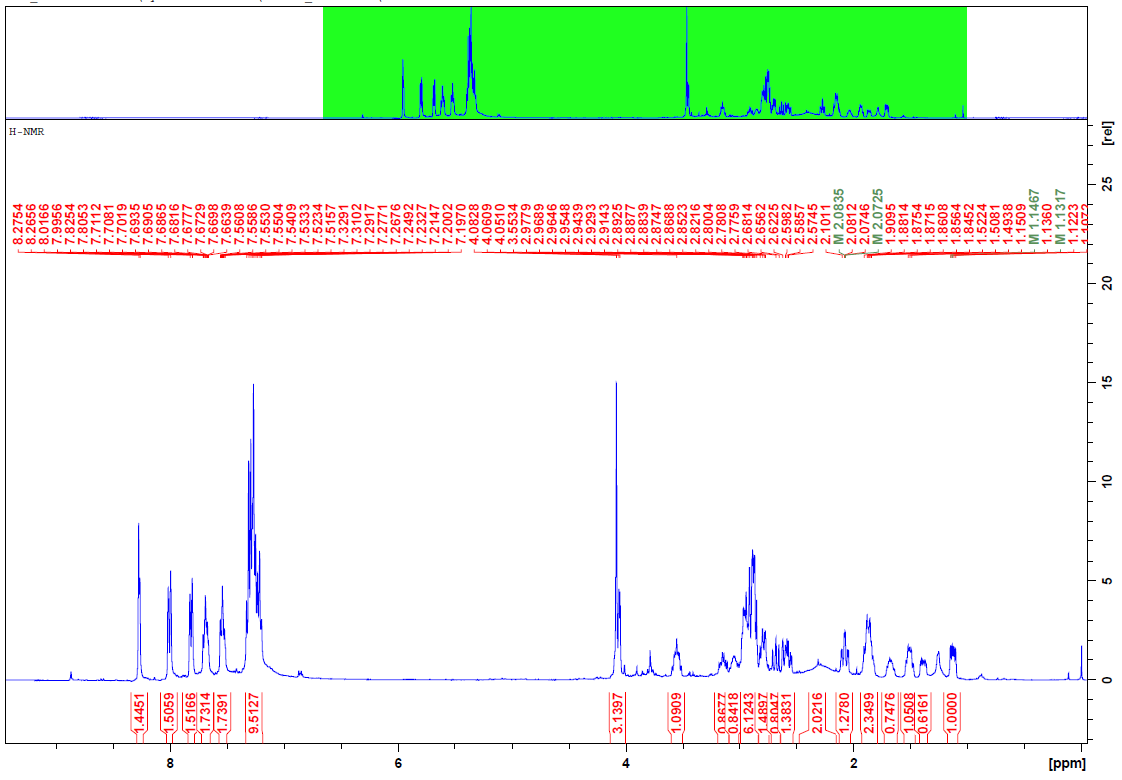
**

**
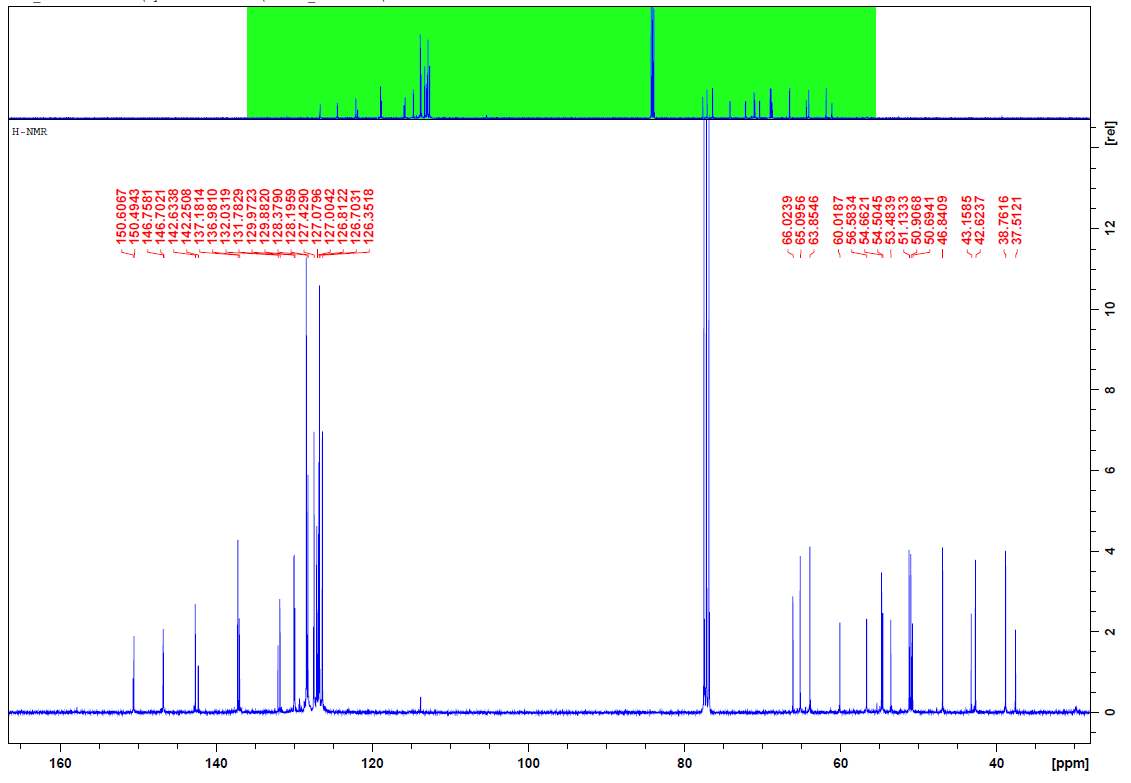
**

**16a**

**
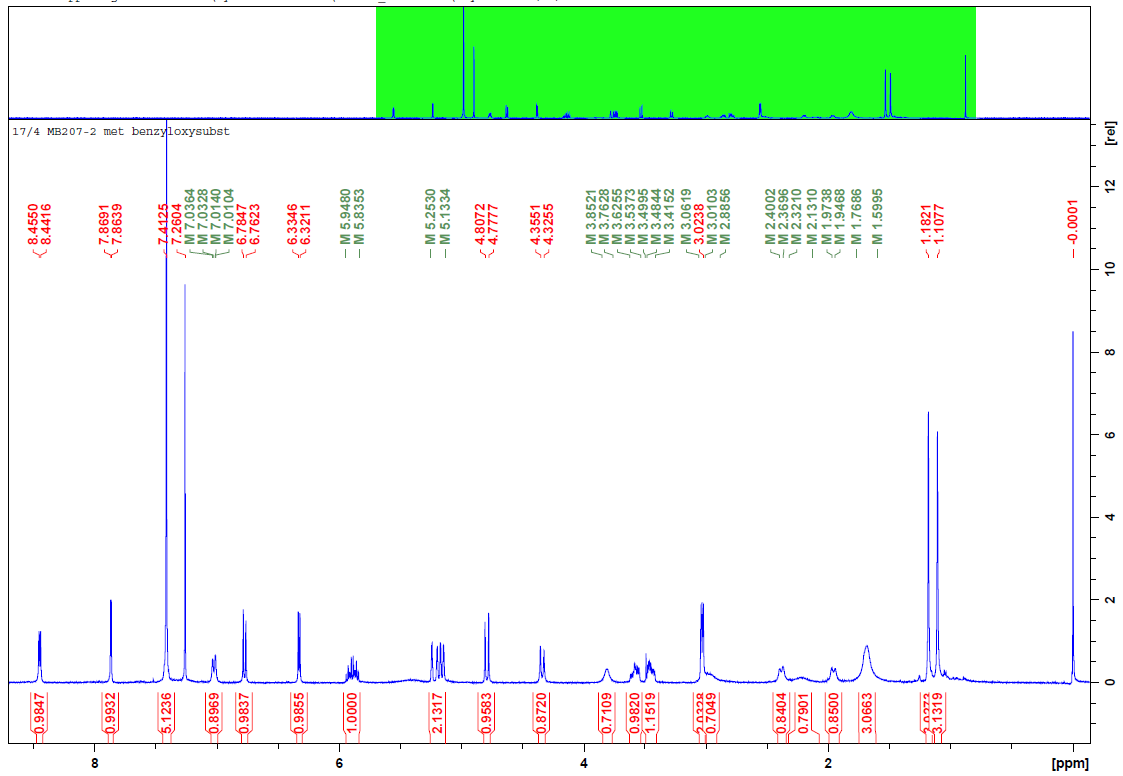
**


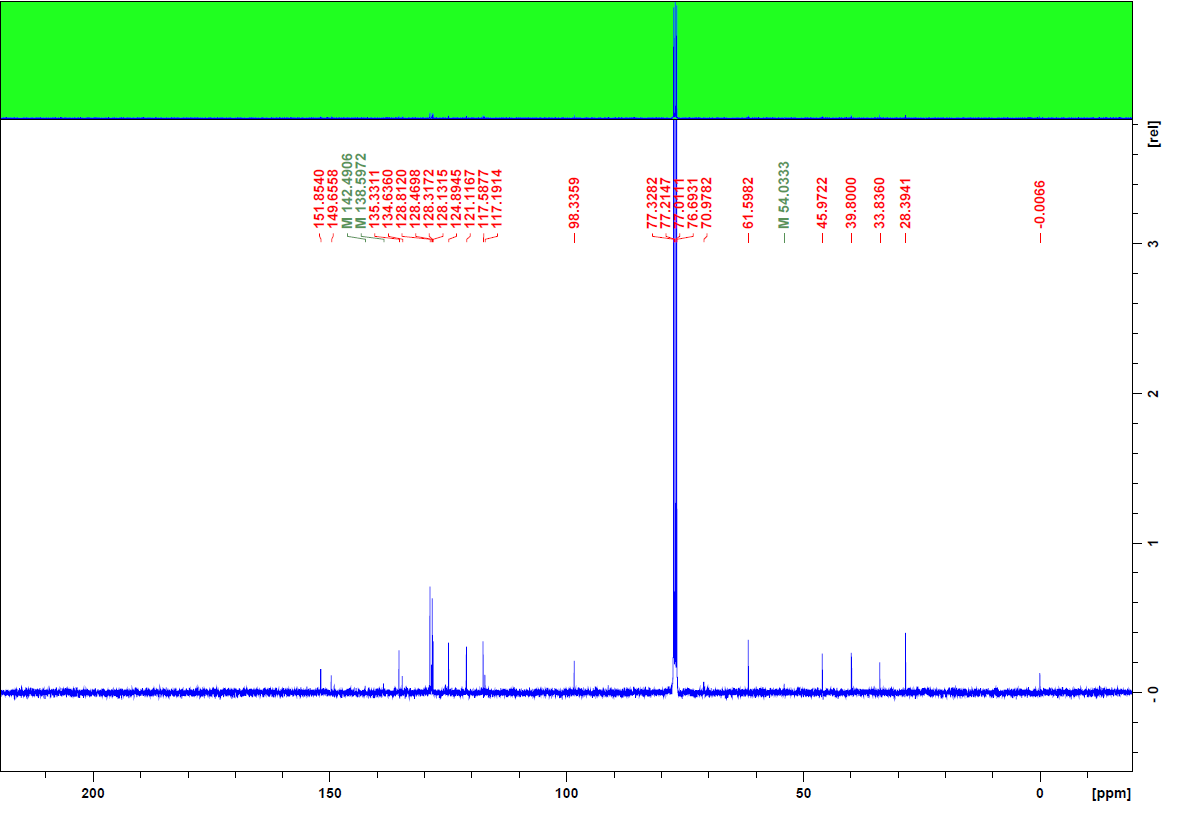


**16b**

**
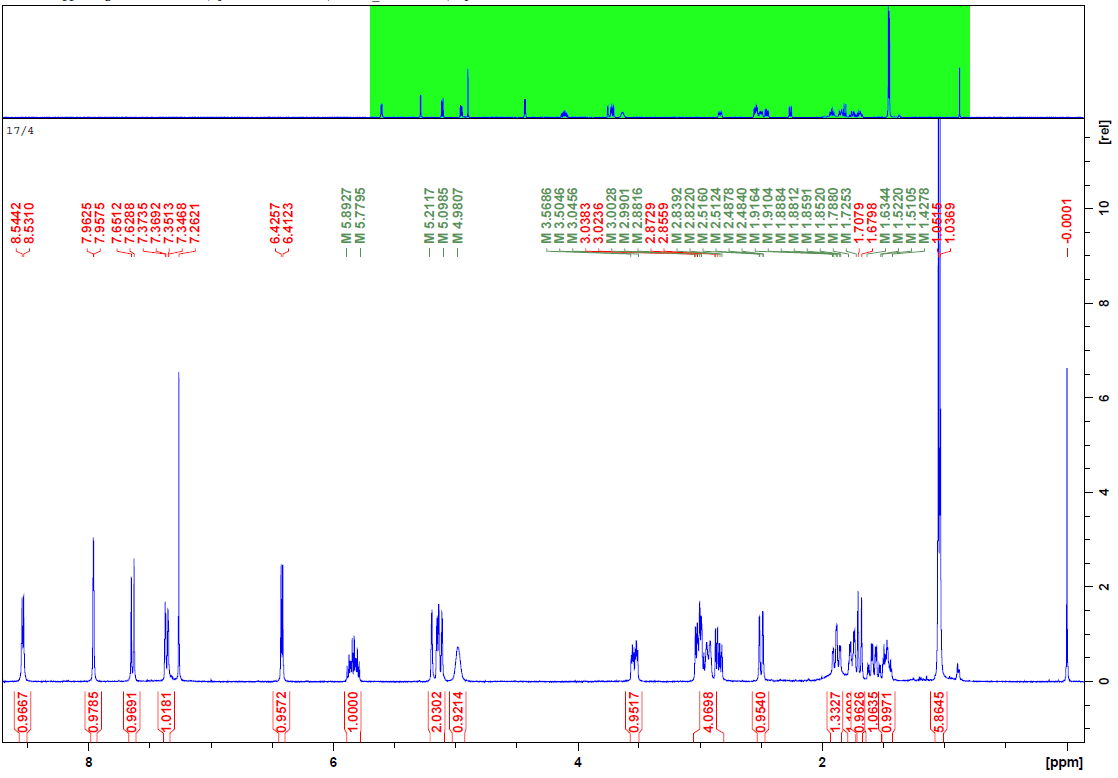
**

**
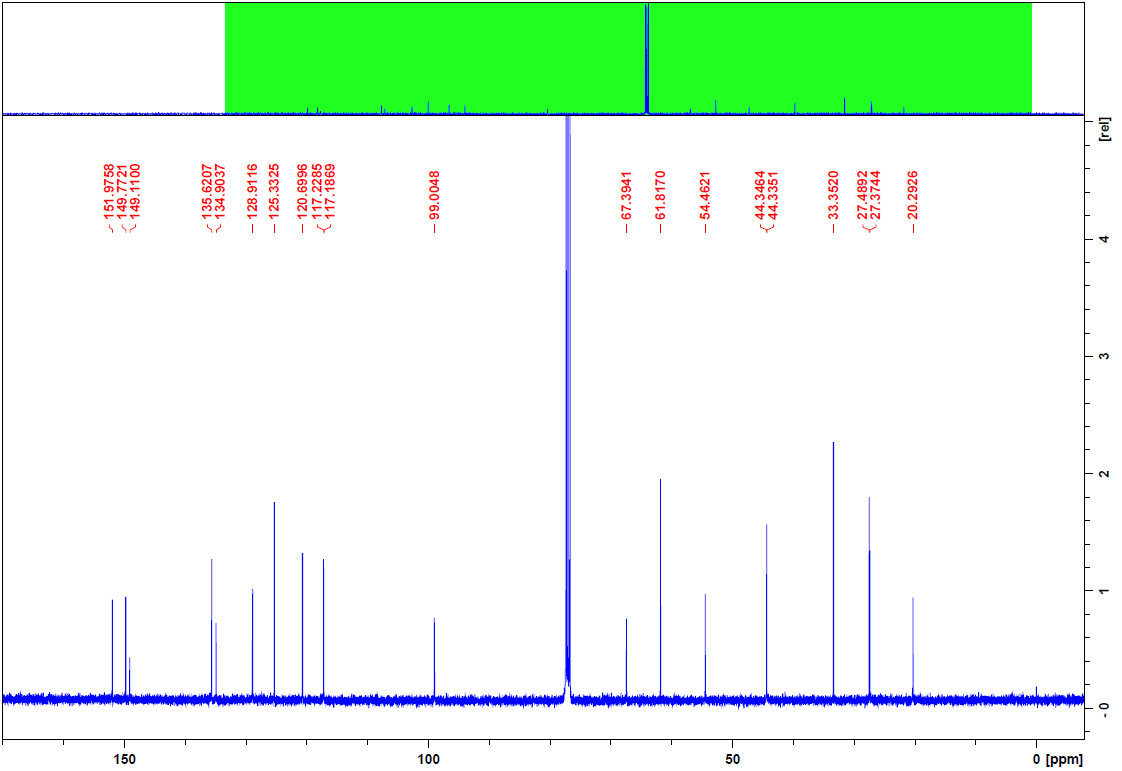
**

**16c**

**
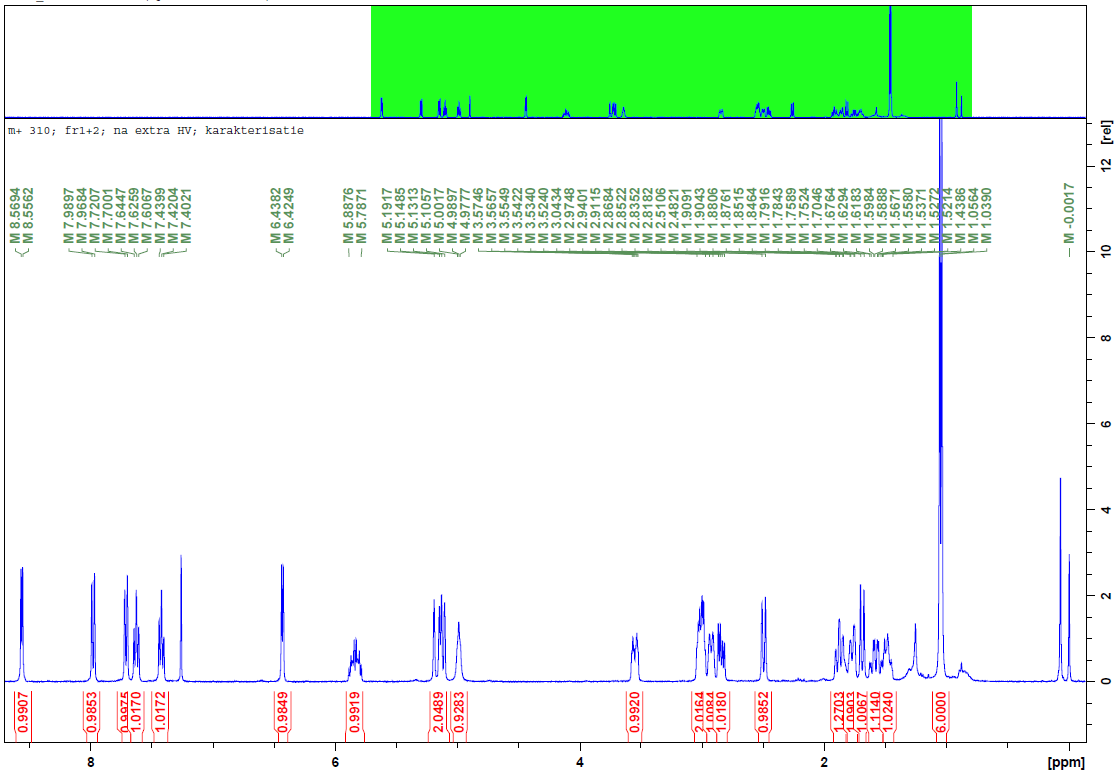
**

**
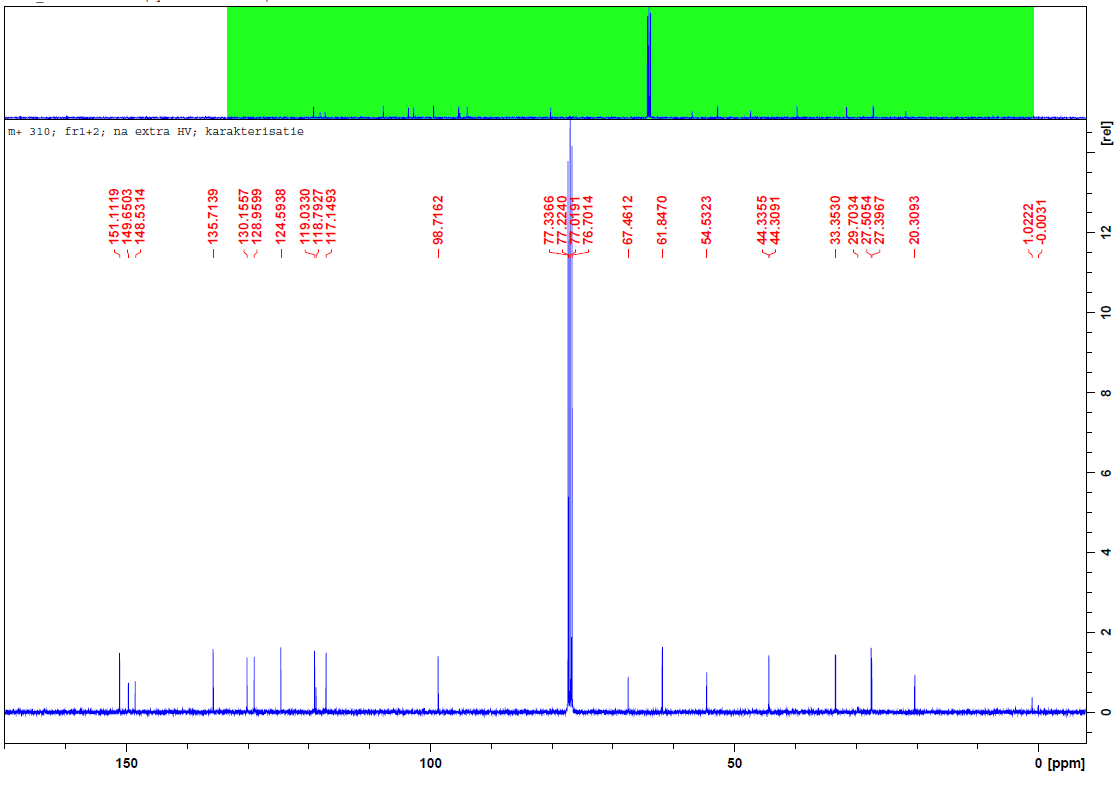
**

**17a**

**
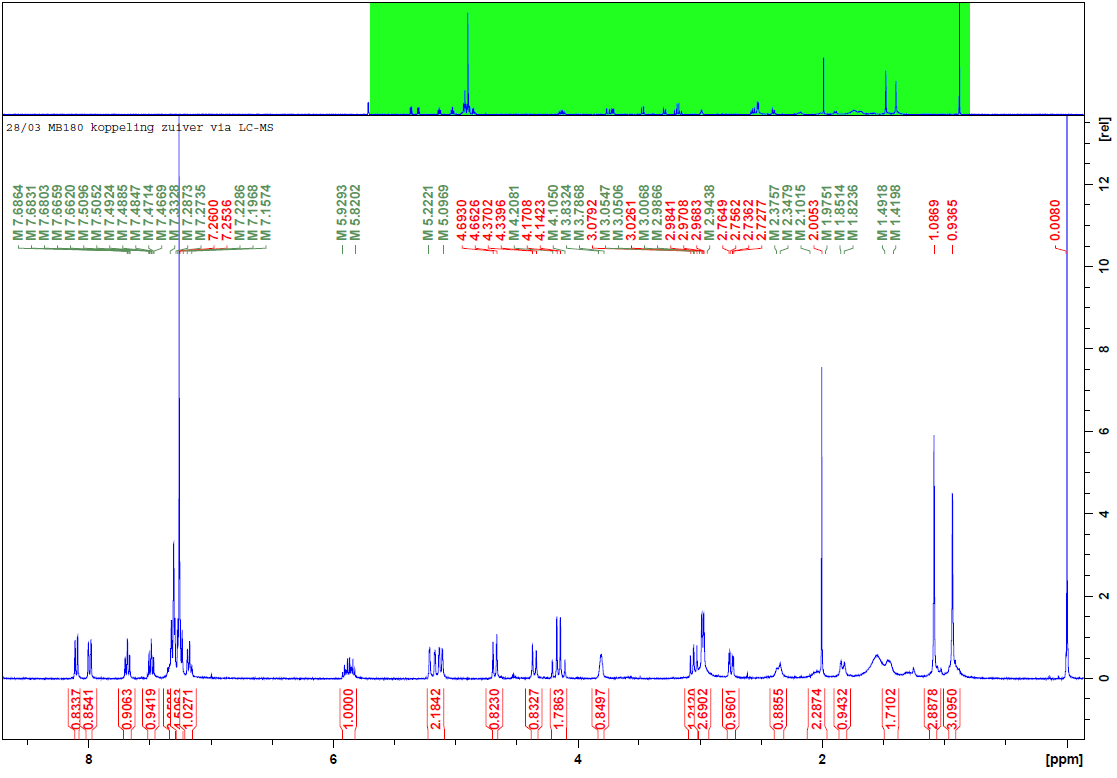
**


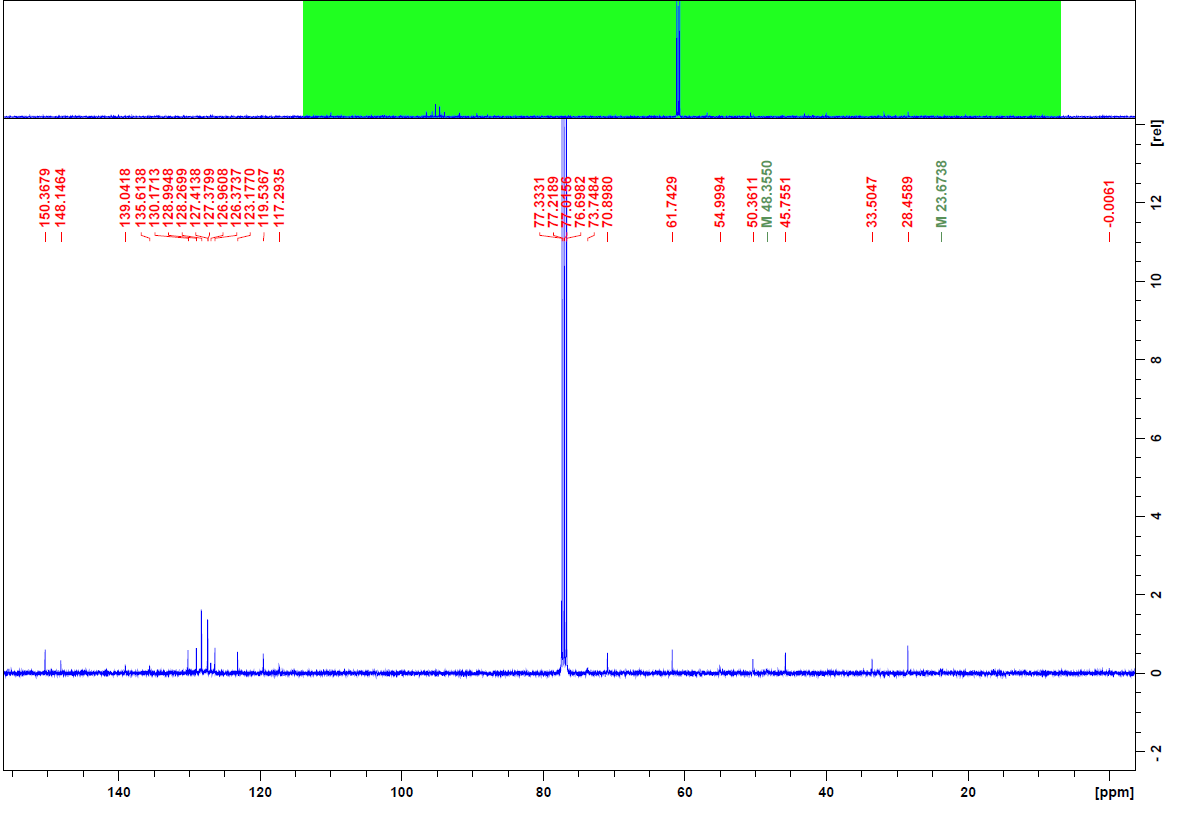


**17b**


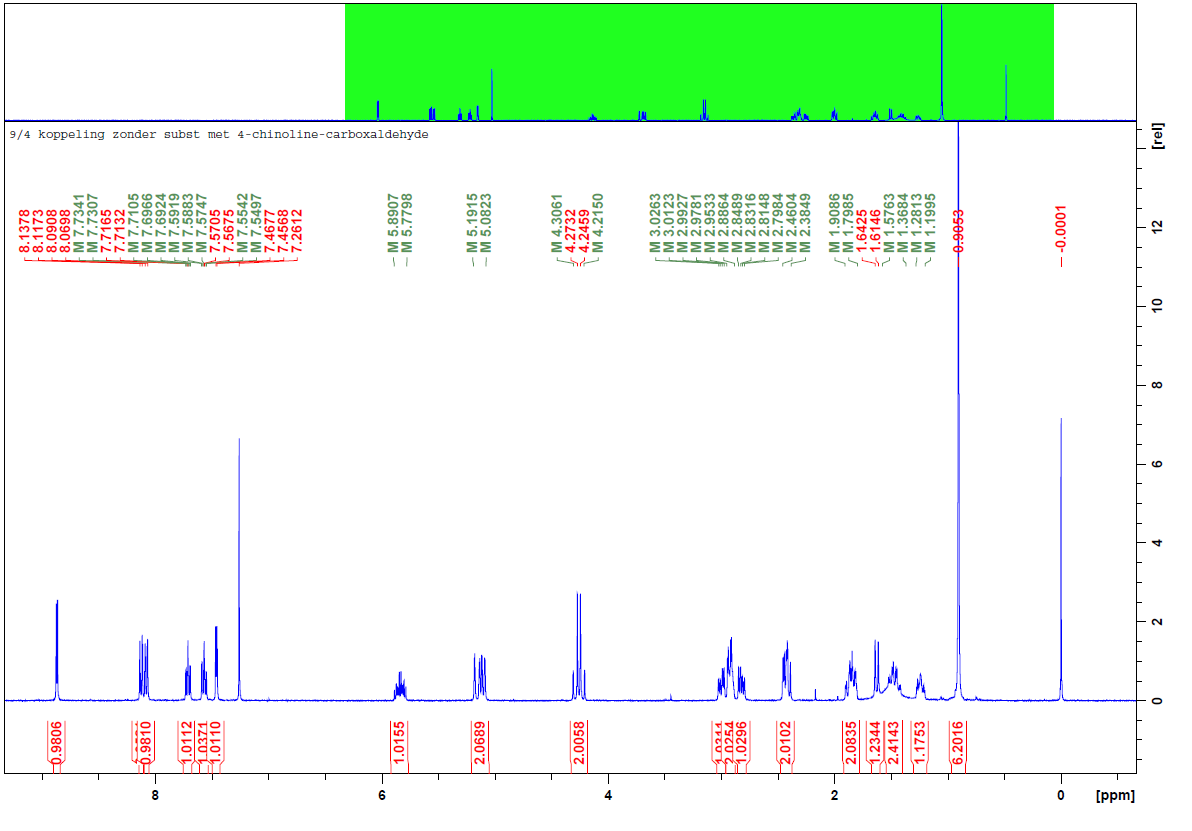


**
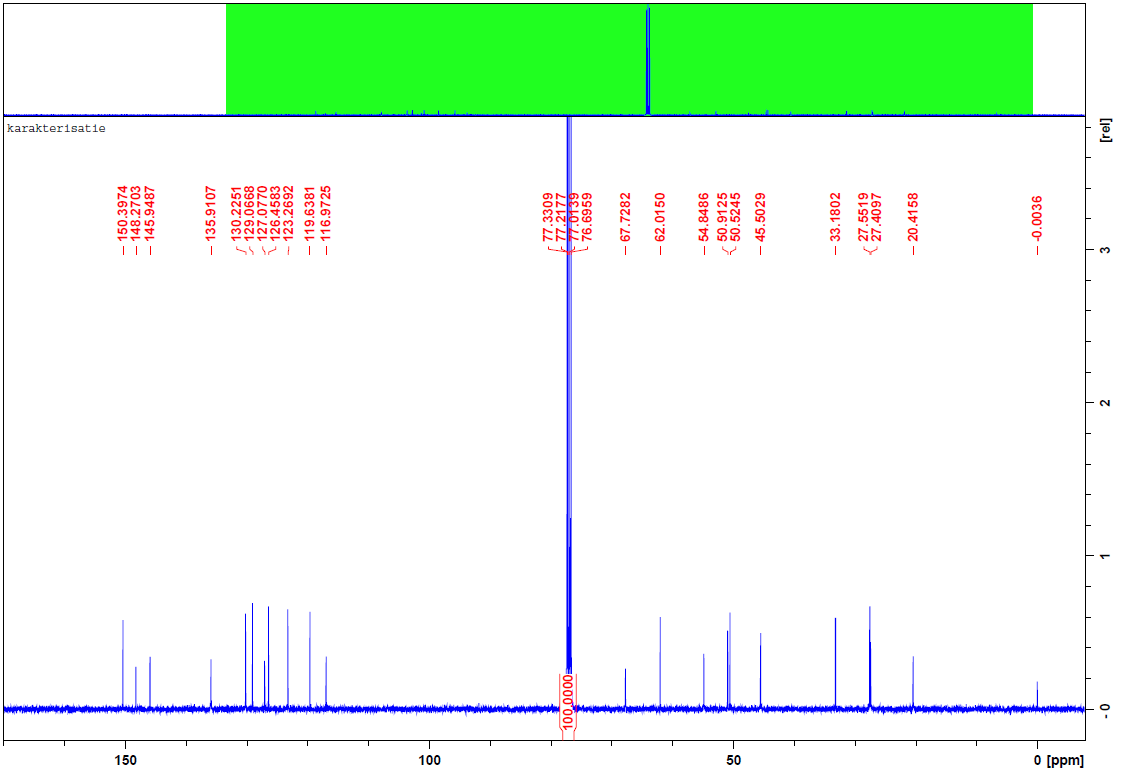
**

**17c**

**
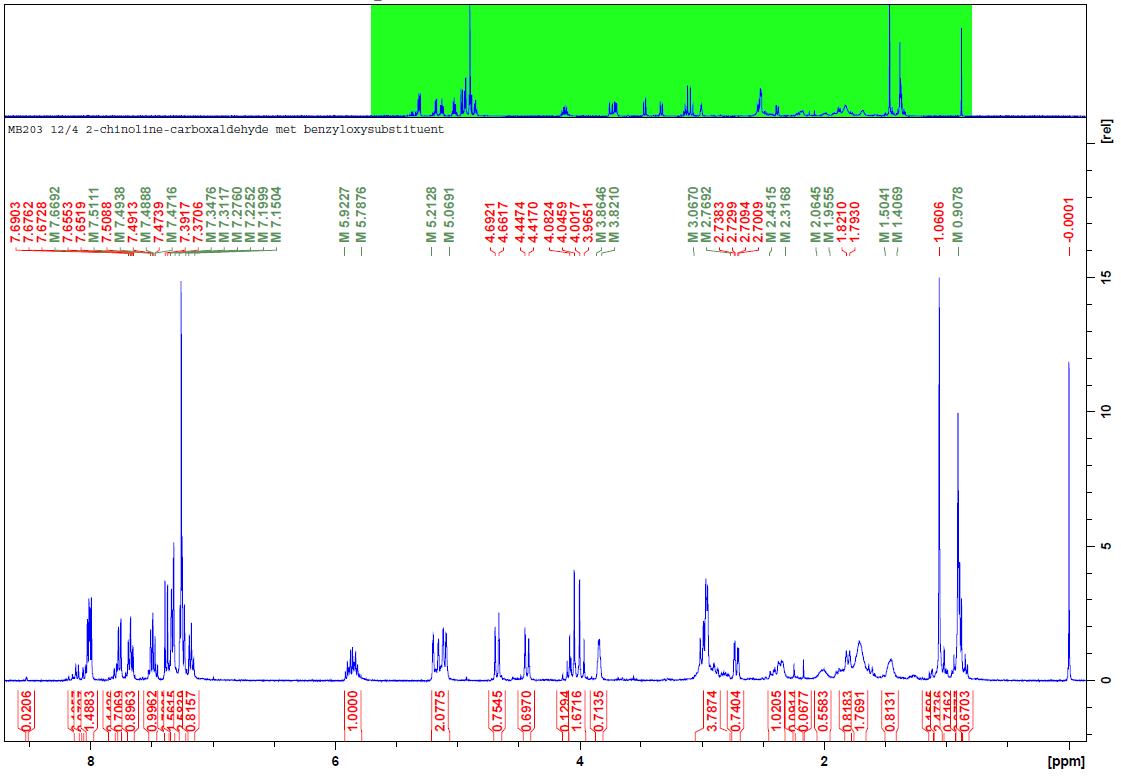
**

**
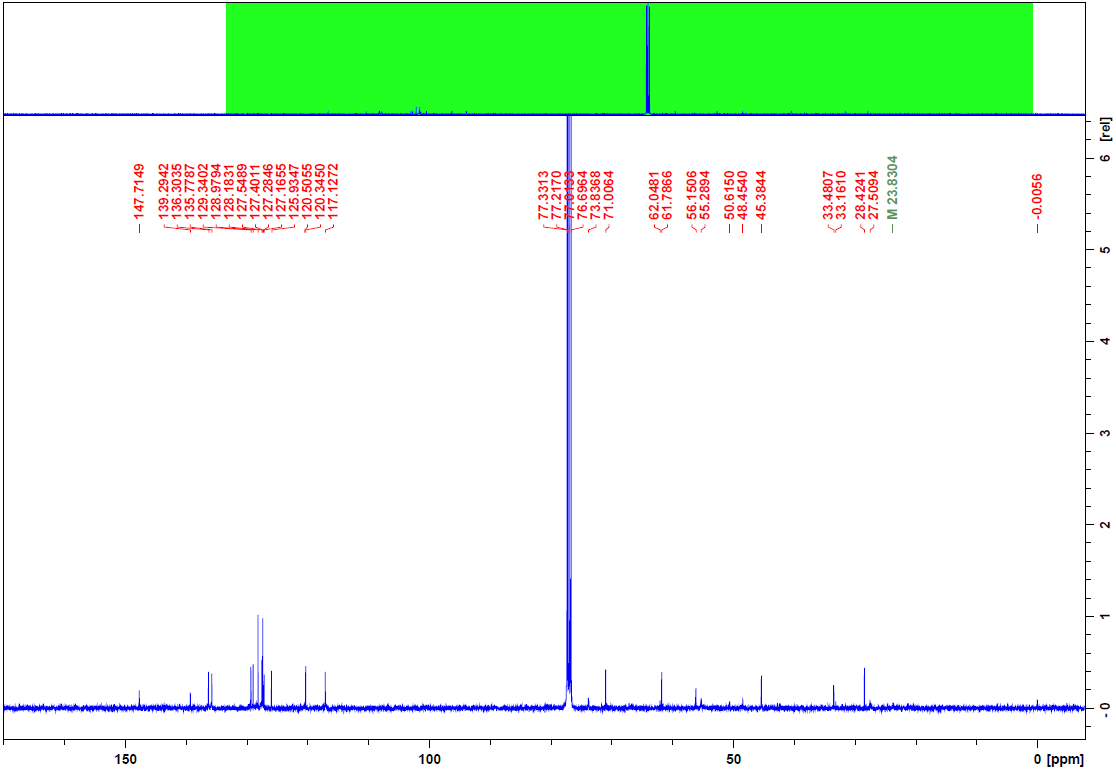
**

**17d**

**
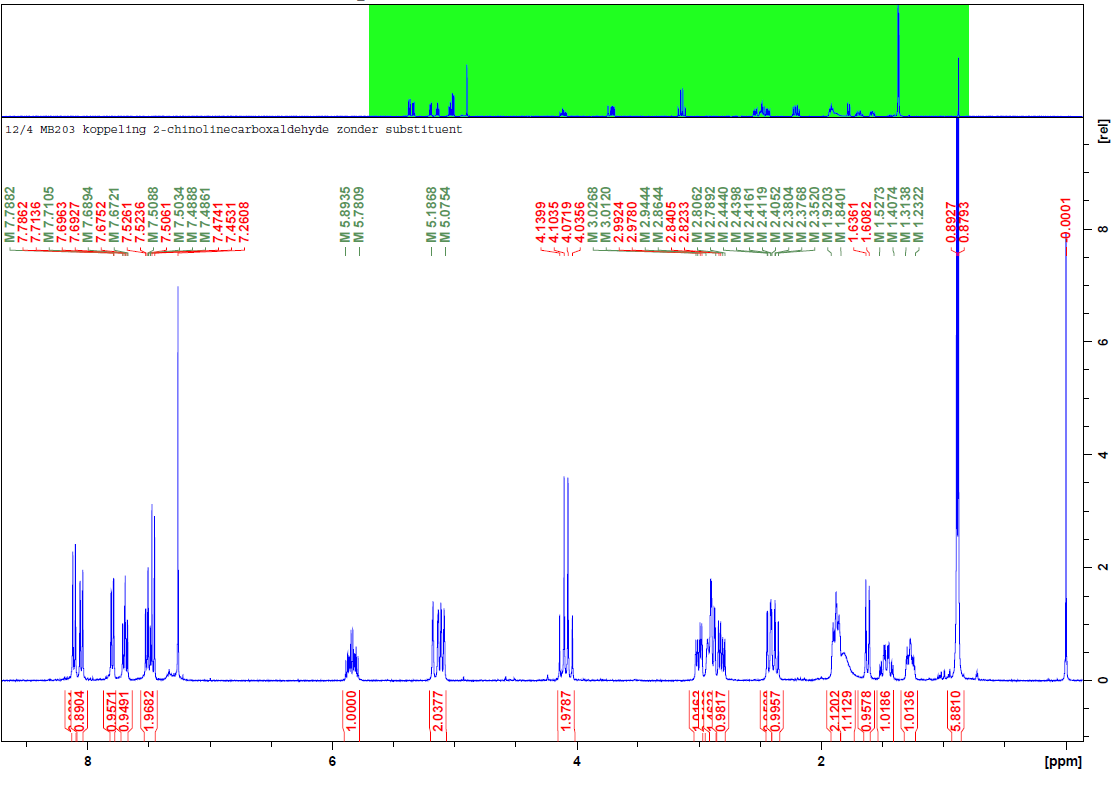
**

**
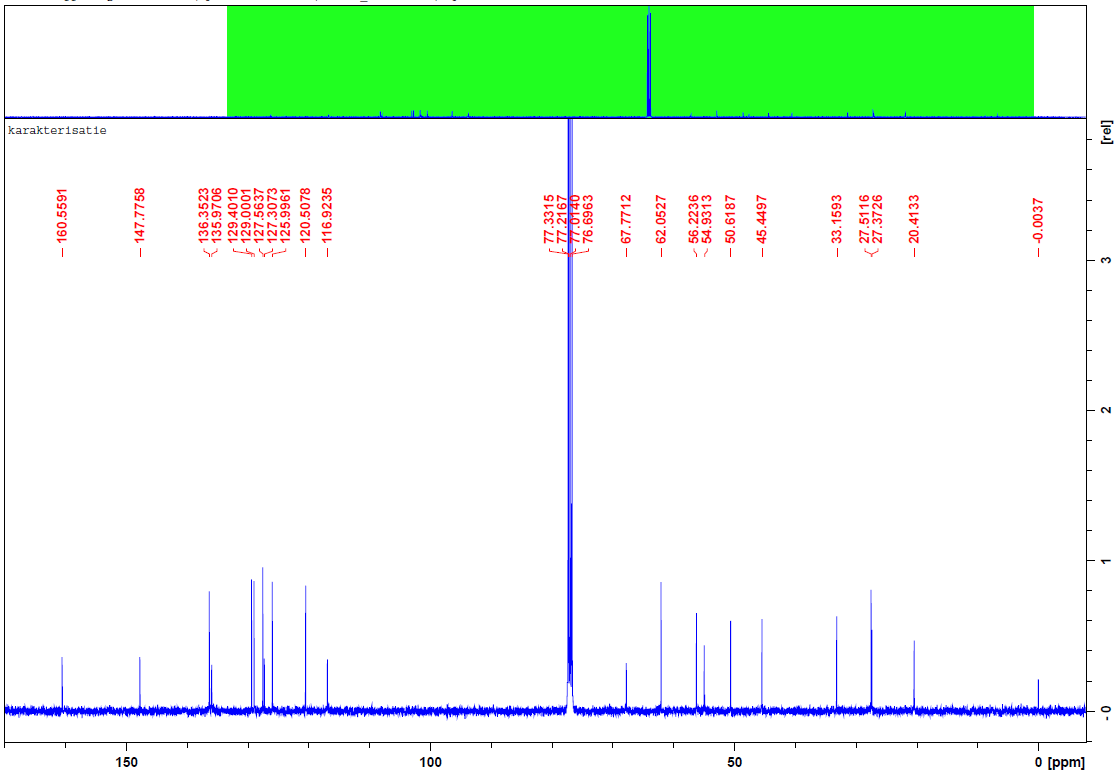
**

**17e**

**
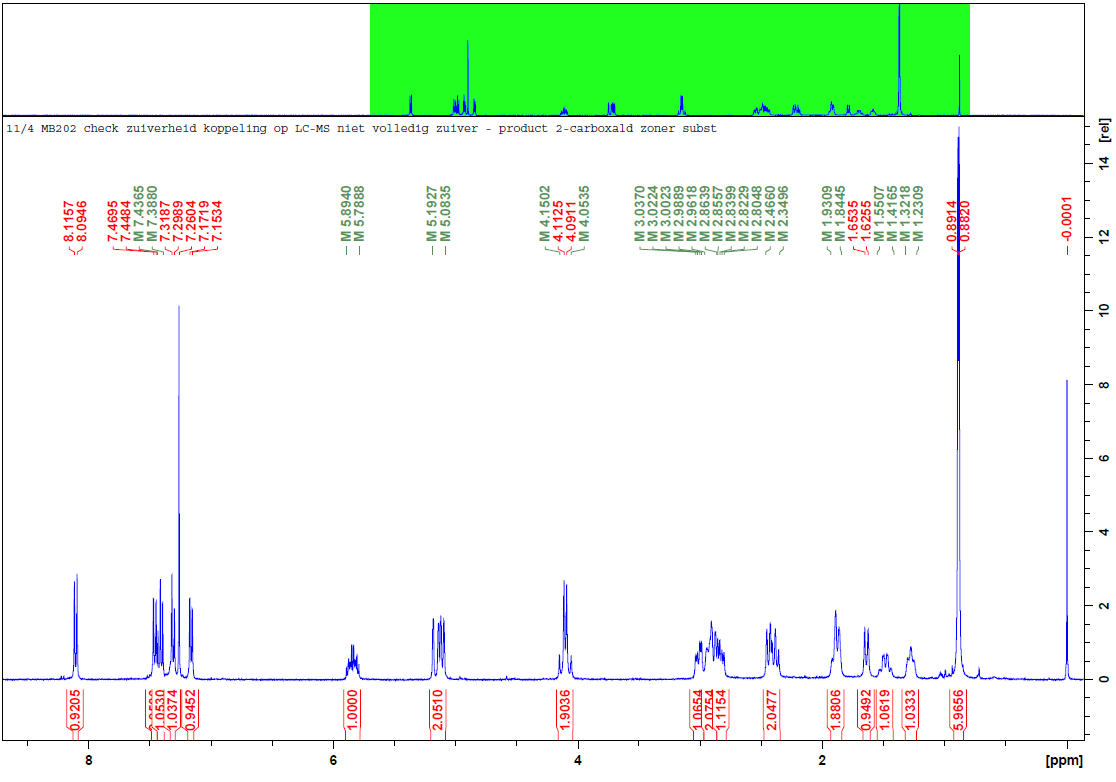
**

**
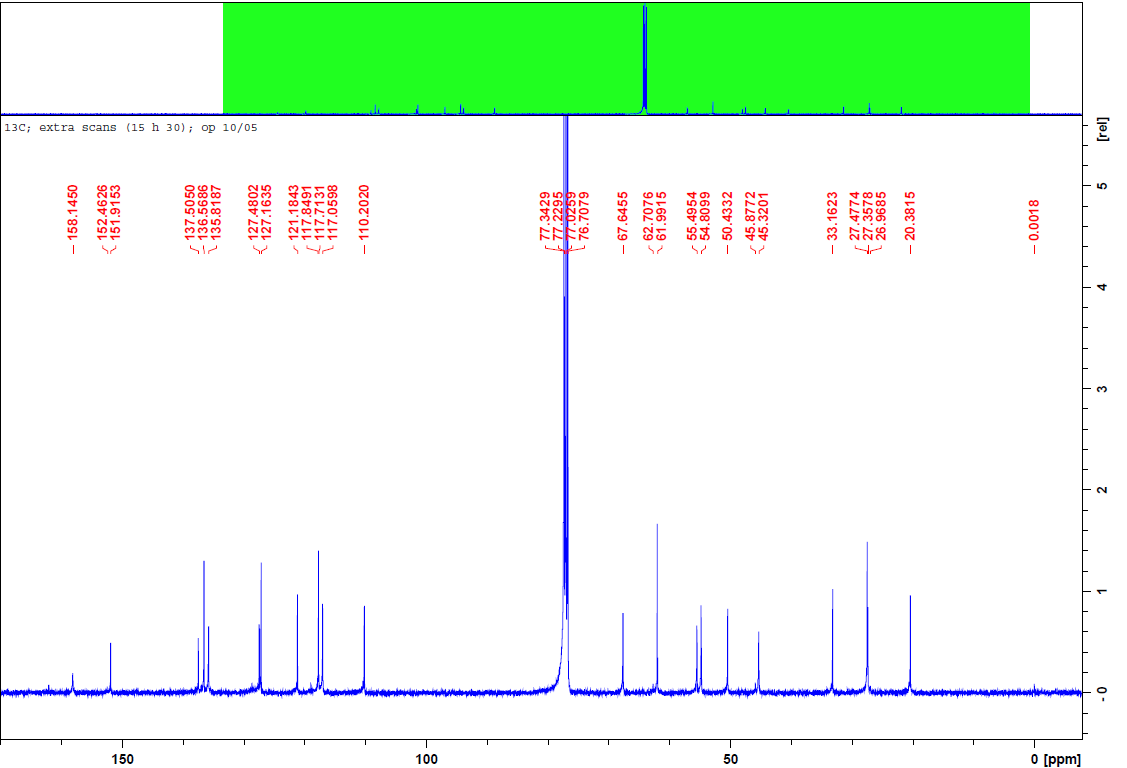
**

**17f**

**
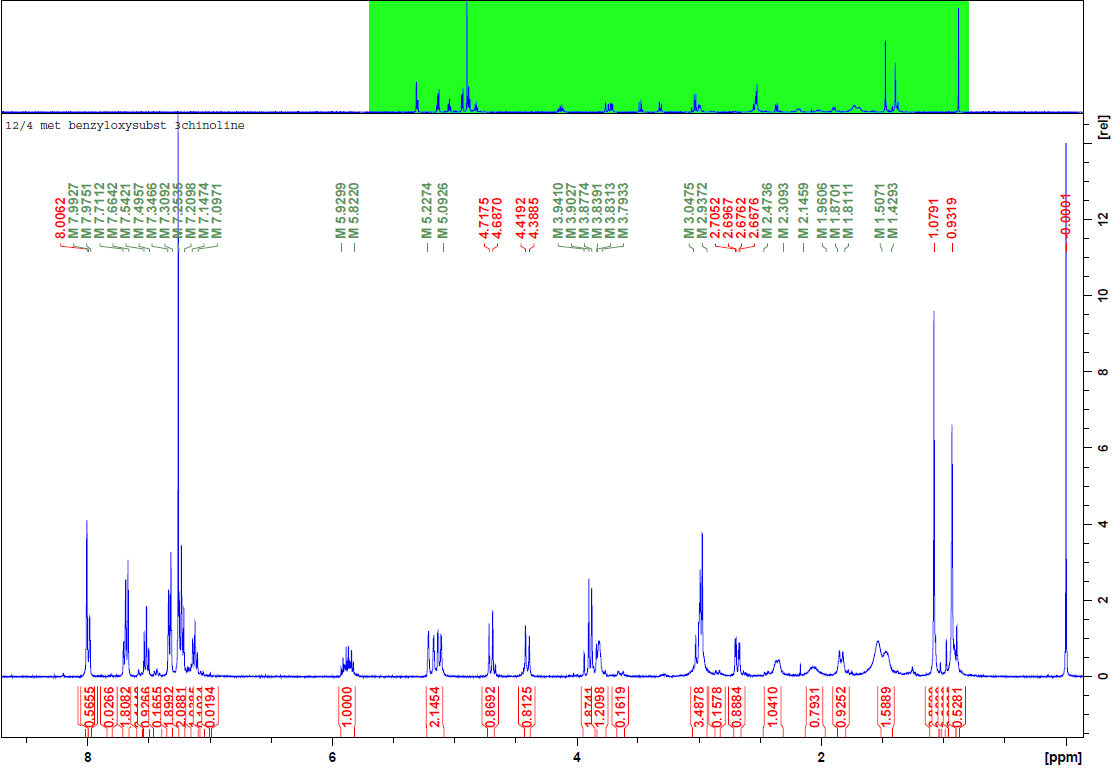
**

**
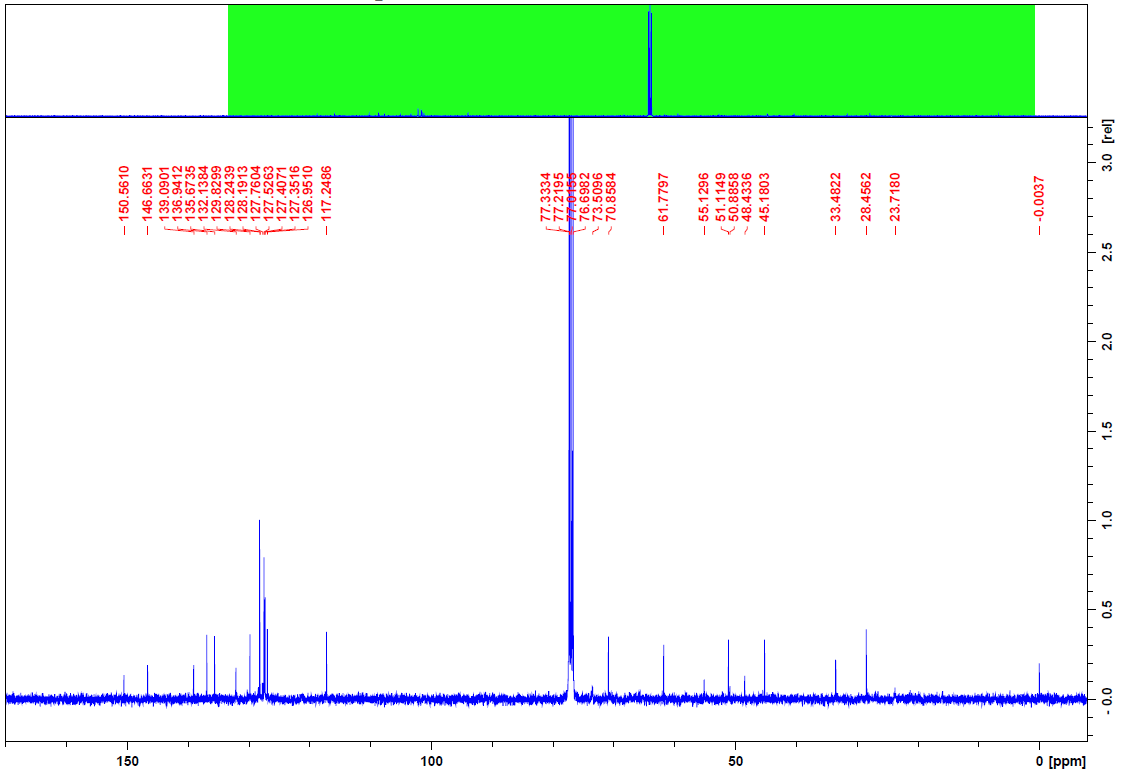
**

**17g**

**
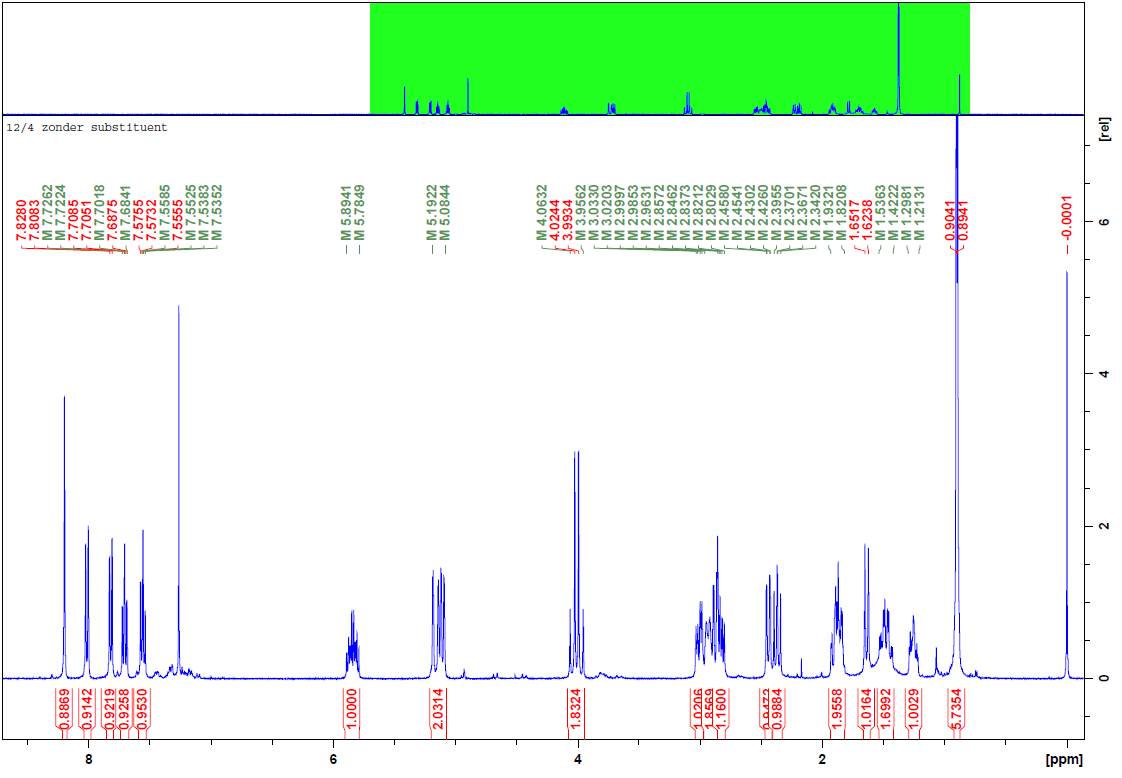
**

**
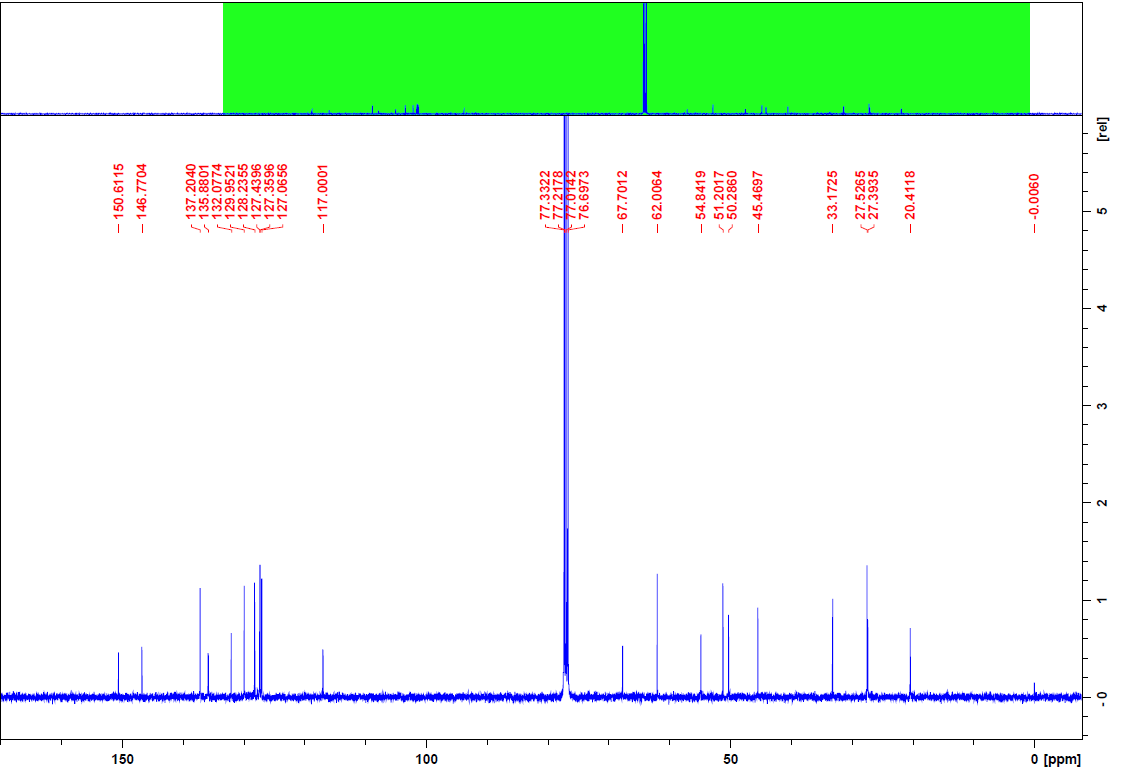
**

**17h**


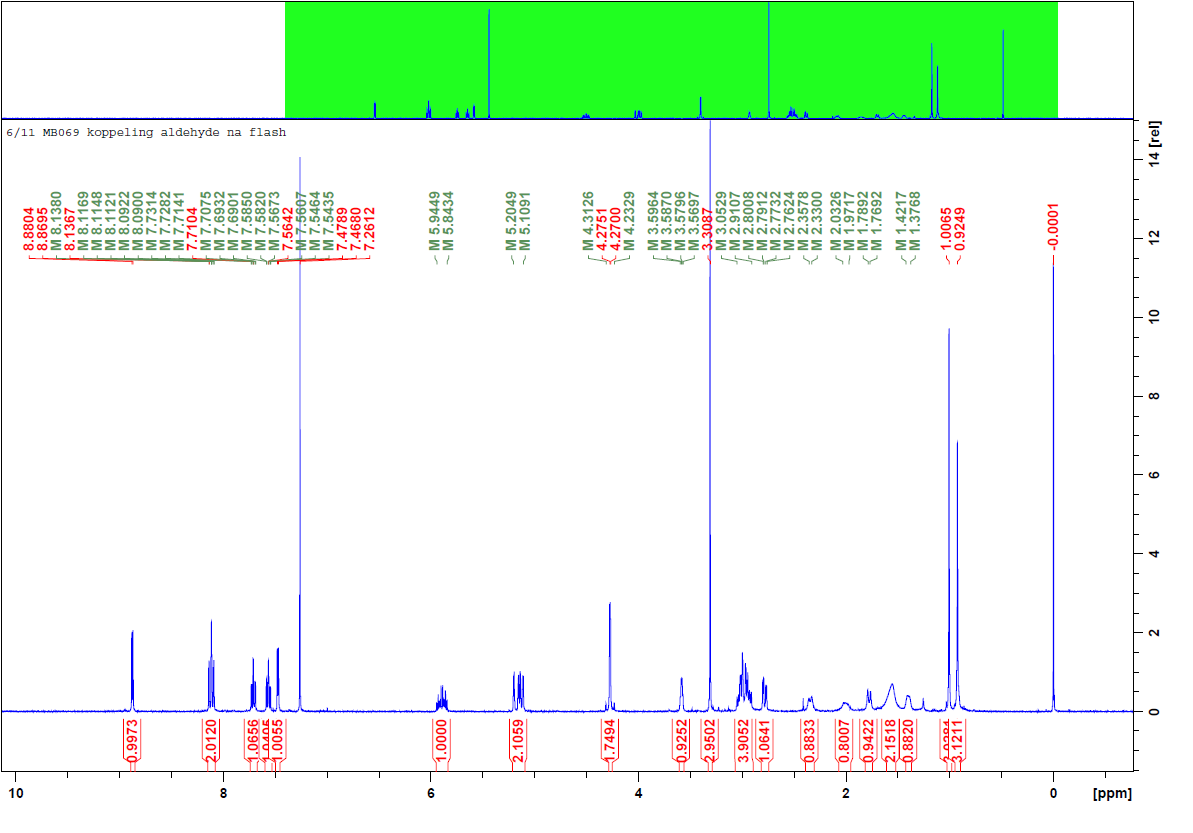


**
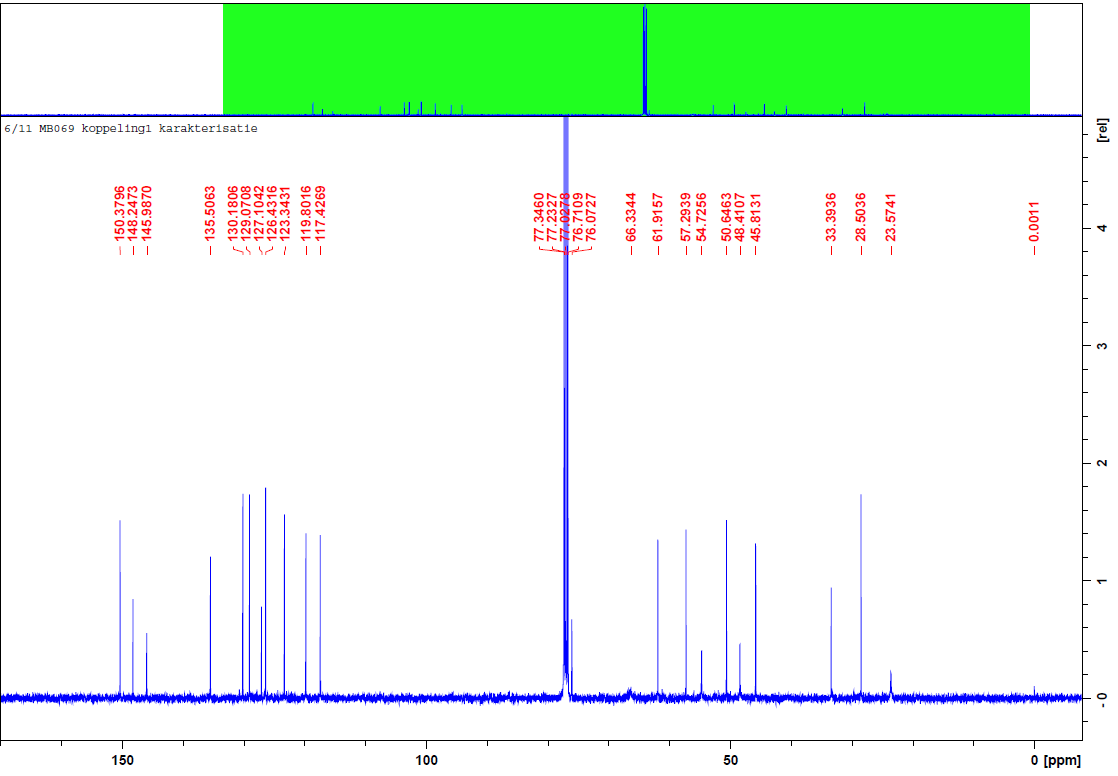
**

# References

[1] K.A. Tehrani, T. NguyenVan, M. Karikomi, M. Rottiers, N. De Kimpe, Electron transfer induced ring opening of 2-(bromomethyl)aziridines by magnesium in methanol, Tetrahedron. 58 (2002) 7145–7152. https://doi.org/10.1016/S0040-4020(02)00728-7.

[2] M. D’hooghe, A. Waterinckx, N. De Kimpe, A novel entry toward 2-imino-1,3-thiazolidines and 2-imino-1,3-thiazolines by ring transformation of 2-(thiocyanomethyl)aziridines, J. Org. Chem. 70 (2005) 227–232. https://doi.org/10.1021/jo048486f.

[3] K. Vervisch, M. D’hooghe, K.W. Törnroos, N. De Kimpe, A new approach towards 1-phenyl and 1-benzyl substituted 2-(aminomethyl)cyclopropanecarboxamides as novel derivatives of the antidepressant Milnacipran, Org. Biomol. Chem. 7 (2009) 3271–3279. https://doi.org/10.1039/b904611a.

[4] E.J. Corey, J.W. Suggs, Pyridinium chlorochromate. An efficient reagent for oxidation of primary and secondary alcohols to carbonyl compounds, Tetrahedron Lett. 16 (1975) 2647–2650. https://doi.org/10.1016/S0040-4039(00)75204-X.

[5] P. Sulmon, N. De Kimpe, R. Verhé, L. De Buyck, N. Schamp, Synthesis of ß-Chloroimines, Synthesis (Stuttg). 3 (1986) 192–195. https://doi.org/10.1055/s-1986-31508.

[6] P. Sulmon, N. De Kimpe, N. Schamp, B. Tinant, J.-P. Declercq, Synthesis of azetidines from β-chloro imines, Tetrahedron. 44 (1988) 3653–3670. https://doi.org/10.1016/S0040-4020(01)85996-2.

[7] Y. Dejaegher, N. De Kimpe, Rearrangement of 4-(1-haloalkyl)- and 4-(2-haloalkyl)-2-azetidinones into methyl ω-alkylaminopentenoates via transient aziridines and azetidines, J. Org. Chem. 69 (2004) 5974–5985. https://doi.org/10.1021/jo040161b.

[8] W. Van Brabandt, R. Van Landeghem, N. De Kimpe, Ring transformation of 2-(haloalkyl)azetidines into 3,4-disubstituted pyrrolidines and piperidines, Org. Lett. 8 (2006) 1105–1108. https://doi.org/10.1021/ol0530676.
